# Transcriptional responses indicate maintenance of photosynthetic proteins as key to the exceptional chilling tolerance of C_4_ photosynthesis in *Miscanthus* × *giganteus*

**DOI:** 10.1093/jxb/eru209

**Published:** 2014-06-22

**Authors:** Ashley K. Spence, Jay Boddu, Dafu Wang, Brandon James, Kankshita Swaminathan, Stephen P. Moose, Stephen P. Long

**Affiliations:** ^1^Proctor and Gamble, 8700 South Mason-Montgomery Road Mason, OH 45040, USA; ^2^Department of Crop Sciences, University of Illinois, 389 Edward R. Madigan Laboratory, 1201W Gregory Drive, Urbana, IL 61801, USA; ^3^Monsanto Company, Chesterfield Village Research Center, 700 Chesterfield Parkway North, Chesterfield, MO 63017, USA; ^4^Energy Biosciences Institute, University of Illinois, 1200 Institute for Genomic Biology, 1206W. Gregory Drive, Urbana, IL 61801, USA

**Keywords:** C_4_ photosynthesis, chilling, chlorophyll a/b-binding protein, cold, D1 protein, LHCII, low temperature, maize, *Miscanthus*, transcription.

## Abstract

*Miscanthus* × *giganteus* is exceptional among C_4_ plants in its ability to acclimate to chilling (≤14 °C) and maintain a high photosynthetic capacity, in sharp contrast to maize, leading to very high productivity even in cool temperate climates. To identify the mechanisms that underlie this acclimation, RNA was isolated from *M* × *giganteus* leaves in chilling and nonchilling conditions and hybridized to microarrays developed for its close relative *Zea mays*. Among 21 000 array probes that yielded robust signals, 723 showed significant expression change under chilling. Approximately half of these were for annotated genes. Thirty genes associated with chloroplast membrane function were all upregulated. Increases in transcripts for the *lhcb5* (chlorophyll a/b-binding protein CP26), *ndhF* (NADH dehydrogenase F, chloroplast), *atpA* (ATP synthase alpha subunit), *psbA* (D1), *petA* (cytochrome f), and *lhcb4* (chlorophyll a/b-binding protein CP29), relative to housekeeping genes in *M.* × *giganteus*, were confirmed by quantitative reverse-transcription PCR. In contrast, *psbo1*, *lhcb5*, *psbA*, and *lhcb4* were all significantly decreased in *Z. mays* after 14 days of chilling. Western blot analysis of the D1 protein and LHCII type II chlorophyll a/b-binding protein also showed significant increases in *M.* × *giganteus* during chilling and significant decreases in *Z. mays*. Compared to other C_4_ species, *M.* × *giganteus* grown in chilling conditions appears to counteract the loss of photosynthetic proteins and proteins protecting photosystem II typically observed in other species by increasing mRNA levels for their synthesis.

## Summary

C_4_ photosynthesis is impaired typically by chilling, but not in *Miscanthus* × *giganteus*. Global transcript profiling reveals that expression of many key photosynthetic genes is maintained or upregulated during chilling in *M.* × *giganteus*, in contrast to maize, suggesting a basis for chilling-tolerant C_4_ photosynthesis.

## Introduction

*Miscanthus* × *giganteus* (Greef & Deuter ex Hodkinson & Renvoize; Greef and Deuter, 1993; Hodkinson and Renvoize, 2001), a rhizomatous perennial grass and bioenergy crop, appears unique among C_4_ species in its productivity and maintenance of photosynthetic capacity during chilling (≤14 °C) (Beale and Long, 1995; Beale *et al.*, 1996; Naidu and Long, 2004; Long and Spence, 2013). In the closely related C_4_ crop *Zea mays* L., photosynthetic capacity declines rapidly in response to chilling (Long *et al.*, 1983; Naidu *et al.*, 2003; Wang *et al.*, 2008*a*). This limits the growth of *Z. mays* in the US corn belt to the chilling-free period of the year, despite intensive breeding efforts to improve chilling tolerance (Rodriguez *et al.*, 2010). In a side-by-side comparison, *M.* × *giganteus* proved 59% more productive than *Z. mays* within the US corn belt even though both crops converted intercepted solar radiation into biomass with equal efficiency (Dohleman and Long, 2009). The higher productivity of *M.* × *giganteus* results from its ability to produce photosynthetically active leaves and maintain them earlier and to retain photosynthetically competent leaves later in the growing season—times when chilling temperatures damage photosynthesis in *Z. mays* (Dohleman and Long, 2009; Long and Spence, 2013).

Chilling during daylight reduces the maximum quantum yield of photosynthetic CO_2_ assimilation (*ΦCO*_2max_) in *Z. mays* due to a combination of chilling-dependent photoinhibition and impaired synthesis of key proteins of the PSII and the light-harvesting complex (LHC) (Nie and Baker, 1991; Long *et al.*, 1994; Fryer *et al.*, 1995). *M.* × *giganteus* shows little depression in *ΦCO*_2max_ and photosynthetic rate during chilling (Beale *et al.*, 1996; Naidu and Long, 2004; Farage *et al.*, 2006; Wang *et al.*, 2008*a*; Purdy *et al.*, 2013). When grown side-by-side in controlled environments at 14 °C, *ΦCO*_2max_ was decreased significantly, relative to 25 °C, by c.50% in *Z. mays*, but was not significantly decreased in *M. × giganteus*. In the same study, photosystem II (PSII) maximum dark-adapted efficiency (F_v_/F_m_) and operating efficiency (Φ_PSII_) in saturating light were reduced c.20 and 80%, respectively, in *Z. mays* yet were hardly affected in *M. × giganteus* (Naidu and Long, 1994 *).* Previous work has also compared the photosynthetic capacity of *M.* × *giganteus* under chilling conditions to a population of the C_4_ sedge *Cyperus longus* native to southern England at 52° N —an exceptionally high latitude for any C_4_ species (Farage *et al.*, 2006; Long and Spence, 2013). Growth of *M.* × *giganteus* at chilling temperatures ≥8 °C did not alter the number of carbon dioxide molecules fixed per electron transferred through PSII (A/J_PSII_). In contrast, A/J_PSII_ declined significantly in *C. longus*, suggesting that even among C_4_ species native to cool climates, *M* × *giganteus* is exceptional in its ability to maintain high photosynthetic efficiency (Farage *et al.*, 2006). This maintenance of photosynthetic efficiency in *M.* × *giganteus* may in part result from avoidance of chilling-dependent photoinhibition through the dissipation of excess absorbed light energy, termed nonphotochemical quenching (NPQ). Zeaxanthin, a photoprotective xanthophyll involved in inducible NPQ, increased 20-fold in *M.* × *giganteus* grown at 8 °C (Farage *et al.*, 2006). The maintenance of photosynthetic CO_2_ assimilation by *M.* × *giganteus* would further dissipate absorbed excitation energy. Both factors are pivotal in the avoidance of chilling-dependent photoinhibition, which in *Z. mays* is characterized by the accumulation of nonfunctional PSII reaction centres (Fryer *et al.*, 1995).

M. × giganteus grown at 14 °C reaches nearly the same light-saturated rates of CO_2_ assimilation (A_sat_) as plants grown at 25 °C, when both are measured at 25 °C (Naidu and Long., 2004; Wang *et al.*, 2008*a*). Conversely, *Z. mays* leaves from plants grown at 14 °C display a light-saturated leaf CO_2_ uptake rate (A_sat_) <10% of that of plants grown at 25 °C, when both are measured at 25 °C (Naidu and Long, 2004). This ability of *M. × giganteus* to maintain *A_sat_* under prolonged chilling conditions has been correlated with its ability to maintain levels and activities of pyruvate phosphate dikinase (PPDK) and Rubisco when grown at 14 °C that equal or exceed levels for plants at 25 °C (Wang *et al.*, 2008a,b). These two enzymes have been shown to have high metabolic control coefficients for light-saturated C_4_ photosynthesis (Furbank *et al.*, 1997). In contrast, the concentrations and activities of these two enzymes decline markedly in *Z. mays* when transferred to 14 °C (Naidu *et al.*, 2003; Wang *et al.*, 2008*a*). While these differences likely account for maintenance of A_sat_ during chilling acclimation, they do not explain the parallel maintenance of a high maximum quantum yield of leaf *ΦCO*_2max_ (Naidu and Long, 1994). The initial slope of the response of leaf CO_2_ assimilation (A) to photon flux (i.e. *ΦCO*_2max_) provides an important measure of capacity for light-limited photosynthesis. In *Z. mays*, a decline in *ΦCO*_2max_ is attributed to loss of efficiency of PSII, which is associated with impairment in the synthesis of key PSII and LHC proteins (Nie and Baker, 1991; Fryer *et al.*, 1995; Caffarri *et al.*, 2005 *).*

While the comparative physiological aspects of chilling tolerance of photosynthesis in *M*. × *giganteus* relative to *Z. mays* have been studied extensively, the molecular basis of this difference is largely unexplored. Analysis of gene expression in chilling-intolerant *Z. mays* during acclimation to 14 °C showed significant downregulation in transcripts encoding chlorophyll a/b-binding proteins, Rubisco, and PPDK (Trzcinska-Danielewicz *et al.*, 2009; Wang *et al.*, 2008*a*,*b*). The current study asked whether the demonstrated physiological tolerance of *M.* × *giganteus* to chilling compared to *Z. mays* corresponds to maintenance or upregulation of a wider range of genes encoding key aspects of the photosynthetic apparatus, using the chilling treatments of previous physiological studies (Wang *et al.*, 2008*a*,*b*). Particular attention is given to those proteins shown to become damaged or deficient in *Z. mays* during acclimation to chilling associated with the decline in *ΦCO*_2max_.

## Materials and methods

### Biological materials

Plant propagation, chilling treatment, and leaf sample collection were performed in two independent experiments for *M.* × *giganteus*. The leaf samples from the first experiment were used in the microarray analysis and quantitative reverse-transcription PCR (qPCR) validation, and the leaf samples from the second experiment, which included a side-by-side comparison with *Z. mays*, were used for the comparisons of transcripts by qPCR and amounts of specific proteins.

Experiments followed the treatment protocol of Wang *et al.* (2008*a*,*b*). Briefly, plants were first grown in warm conditions of 25 °C days and then transferred to 14 °C for 14 days. Prior studies have shown that this is sufficient time for completion of acclimation of photosynthesis in mature leaves of both species. In the first experiment, *M.* × *giganteus* rhizomes were propagated in soil-less potting media (Sunshine Mix LC1^2^, Sun Gro Horticulture, Bellevue, WA, USA) in 1.2-l pots following the procedure of Naidu and Long (2004). Eight individual plants were grown in two identical controlled-environment growth chambers (Conviron E15, Controlled Environments, Winnipeg, Manitoba, Canada) under 500 μmol photons m^–2^ s^–1^ incident light provided by an equal mixture of high-pressure mercury and sodium lamps, in a 14/10 light/dark cycle at either 14/12 °C (chilling) or 25/20 °C (control). Both chambers were maintained at 70% relative humidity. The eight plants were initially grown in the control chamber for 2 weeks, and then half of these plants, selected by fully randomized design, were transferred to the chilling conditions. After 2 weeks of chilling, and 1 month of total growth, leaf samples were taken 2–3h after illumination began (i.e. mid-morning) from the midpoint of the youngest fully expanded leaf (Naidu and Long, 2004; Wang *et al.*, 2008*b*). Full expansion was defined by emergence of the ligule. Shoots had approximately four leaves each at the time of sample collection. All leaf samples were immediately plunged into liquid nitrogen and then transferred to storage at –80 °C until RNA extraction for microarray and qPCR analysis.

The conditions for the second experiment were identical to the first apart from the following addition. *Z. mays* cv. B73 seeds were germinated in 1.2 liter pots in soil-less potting media following the procedure of Naidu and Long (2004) and grown alongside *M.* × *giganteus* following the sampling and conditions described above.

### 4×44K Agilent oligonucleotide two-colour cDNA maize microarray

#### RNA extraction

Total RNA was extracted from the leaf samples. Samples were homogenized in 1ml TRIZOL Reagent (Invitrogen, Carlsbad, CA, USA) using a glass-Teflon homogenizer. The homogenized samples were then incubated for 5min at room temperature to permit the complete dissociation of the nucleoprotein complexes. Each 1ml sample had 0.2ml chloroform added, was incubated at room temperature for 150 s, and was centrifuged at 12 000 *g* for 15min at 4 °C. The RNA in the resulting supernatant was precipitated with 0.5ml isopropyl alcohol and was isolated by centrifugation at 12 000 *g* for 10min at 4 °C. The RNA pellet was dried and redissolved in 50-μl RNase-free water for 10min at 55 °C and then further purified with the RNeasy Kit (Qiagen, USA). Final RNA quantity was determined spectrophotometrically at λ 260nm (NanoDrop ND1000, NanoDrop Technologies, Wilmington, DE, USA). The purity of RNA was assessed by the 260/280 absorbance ratio. Only those RNA samples with ratios of ≥2.0, indicating no significant presence of contaminants, were used.

#### Microarray fluorescent cDNA synthesis and hybridization

cDNA was synthesized from total RNA using a Agilent Low RNA Input Linear Amplification Kit, with spike-in controls (5184-3523), following the manufacturer’s instructions (Agilent Technologies, Santa Clara, CA, USA). Two of the four control samples and two of the four treatment samples were labelled with Cy3 dye and the other samples were labelled with Cy5 dye, to account for any possible dye-bias that may occur during hybridization or fluorescence detection during scanning of the slides. Agilent 4×44K oligonucleotide two-colour cDNA Maize microarray slides were used. Hybridization and washing was performed following the Agilent Two-Colour Microarray-Based Gene Expression Analysis protocol (Agilent Technologies). A total of four 4×44k slides (i.e. four technical replicates) were used for each of the four biological replicates. Hybridized slides were scanned with a GenePix4000B microarray scanner (Axon Instruments, Concord, ON, Canada) and features were extracted and analysed using GenePix Pro 6.1 (Axon Instruments). Each array was inspected individually and low-quality spots (i.e. those with fluorescence levels that were not significantly distinguishable from the background fluorescence in the red-to-green channel intensity ratio histograms of the array) were manually flagged and eliminated from further analysis.

#### Microarray statistical analysis

All calculations were performed using the R computing environment (Venables *et al.*, 2013). Normalization and statistical analysis was done using the R package Limma provided by the Bioconductor repository (http://bioconductor.org; Wettenhall and Smyth, 2004). No background correction was performed, a common practice for spotted arrays with low background (Smyth *et al.*, 2003, 2005). The global loess function was employed for within-array normalization and the scale method was applied for between-array normalization (Smyth and Speed, 2003). Test-statistics were determined using eBayes fit and topTable functions (Smyth, 2004). Use of the duplicate correlation function accounted for duplicated probe spots on the Agilent array and for technical replication (Smyth *et al.*, 2005). The lmFit function was applied to fit the data to a linear model using quantitative nonnegative weights for flagged spots, and a design matrix was used to compare expression values in the control vs. treatment arrays. Genes that were differentially expressed at 14 °C versus 25 °C were defined on the basis of a significant *P*-value, adjusted for multiple testing, using FDR (adj. *P*-value 0.00001) and a log_2_ fold-change cut off of 0.70 or –0.70. These criteria were validated by the ability to also detect significant expression differences by qPCR, as defined previously (Wurmbach *et al.*, 2003).

### Gene annotation and MapMan application

Sequences for the FASTA 60-mer probes on the Agilent 4×44K oligonucleotide two-colour cDNA Maize microarray slides were provided by Dr. Virginia Walbot at Stanford University. A gene annotation file for the Agilent 60-mer probe sequences was generated by BLASTN of probe sequences to the *Z. mays* B73 reference genome sequence, build 2 (Schnable *et al.*, 2009). For genes found to change significantly between control and treatment, but lacking any published annotation at the time of the experiment, annotation was attempted by performing a tblastn BLAST query (http://www.ncbi.nlm.nih.gov/) on the corresponding 60-mer probe FASTA sequence.

To visualize the differentially expressed genes, the MapMan software tool was used (Usadel *et al.*, 2009). The MapMan software utilizes the Affymetrix platform to generate hierarchical categories of gene function and pathway placement. In order to use this software, to visualize results obtained here on the Agilent microarray, a ‘mapping file’ was created based upon the pre-existing *Z. mays* ontology/mapping file with Affymetrix-assigned probe identifiers. The Agilent annotations were cross-referenced with the *Z. mays* mapping file and gene annotations matching those in the *Z. mays* mapping file BIN or sub-BIN for functional categories. The Affymetrix identifier was replaced with the Agilent identifier. The Agilent identifier corresponding to the assigned BIN/category was then used to create an experiment file containing the corresponding down- or upregulated log_2_ fold-change value. This experiment file and the new mapping file was then uploaded into the MapMan Image Annotator module (Usadel *et al.*, 2009).

### Quantitative reverse-transcription PCR

Quantitative reverse-transcription PCR was used to validate the up- and downregulation of transcripts of particular interest that were found to be differentially expressed in the microarray experiment. Genes for validation with known *Z. mays* sequences (http://www.ncbi.nlm.nih.gov/) were compared against available *M.* × *giganteus* genome survey sequences obtained by 454 sequencing (Swaminathan *et al.*, 2010) and transcripts assembled from Illumina short-read sequencing (Barling *et al.*, 2013). Sequences with more than 98% identity were candidates for qPCR validation. Six transcripts were chosen: *ndhF* (NADH dehydrogenase F, chloroplast), *atpA* (ATP synthase alpha subunit), *lhcb5* (chlorophyll a/b-binding protein CP26), *psbo1* (oxygen-evolving enhancer protein 1), *aps* (ATP synthase), and *tps* (terpene synthase). The primers for the transcripts were designed based on *M.* × *giganteus* sequences (Table 1). Four candidate reference genes for qPCR were chosen because they are common housekeeping genes that have stable expression levels between the control and treatment samples in the microarray experiment: *tuba6* (alpha-tubulin 6), *tubb3* and *tubb6* (beta-tubulin 3 and 6), and *tubg1* (gamma-tubulin 1).

**Table 1.** Primer sequences for qPCR validation of microarray results and comparison with *Z. mays*

Transcript	Forward (5′–3′)	Reverse (5′–3′)	Expected product size (bp)
*tuba6*	AAGACGCAGCTAACAACTTTGCCC	CACCACCAACAGCATTGAACACCA	140
*tubb3*	ATGGACGAGATGGAGTTCACCGAA	TGATGTACCACAGCAGCGAAGACT	174
*tubb5*	CTTTGTTTCACCTGCACCGCTTGA	AGGAAGGAACCATCACAGGAGCAA	98
*tubb6*	TTCTGACCTTCAGTTGGAGCGTGT	TGCCCAAACACAAAGTTGTCAGGG	164
*tubg1*	ATGCACCTGAGTGGCGAAGTCCT	ATCGGGATGGAGTTCTGGAAGCA	81
*act*	TGAGGCCACGTACAACTCCATCAT	CCTTTCAGGTGGCGCAATCACTTT	180
*ndhF*	ACCCACTCCCATTTCGGCTCTTAT	GAGCAAGAGCTAAAGTGGCTCCTA	166
*atpA*	ATGGCGGATTCACCCGCTACATTA	TGGCGATAAGCTTGTGCCTGTTTG	136
*lhcb4*	AGTAGAAGATGAGCATGGCGAGCA	AGAACTTCGCCAACTTCACCG	180
*lhcb5*	TCGCCATGTTCTCCATGTTCGGAT	TAGGCCCAGGCGTTGTTGTTGA	114
*psbo1*	ACCTACGACGAGATCCAGAGCAA	GCACAGCTTCTTCAGCTGGTACTT	134
*aps*	TGGATGGAGCAGAAGAACAGGGTT	TGCGGTATTTGCCTTTCAGCTTCG	120
*tps*	ATGCGAGCATCAGACAGTTCAGGA	CAGCTACCATTTGCACGGCAGAAA	88
*psbA*	ACTTAGTTTCCGTCTGGGTATGCG	TAAGGATGTTGTGCTCTGCCTGGA	185
*petA*	CGCACATCTATTTCAAACGCATA	TACAATTCGTCCAGTTGCTTCCCG	81

SuperScript-III RT (Invitrogen) was used to synthesize cDNA from the same *M.* × *giganteus* samples used for the microarray experiment. SYBR Green Master Mix (Applied Biosystems, Foster City, CA, USA) was used for qPCR and samples were amplified on a high-throughput microwell-plate-based thermo-cycler (LightCycler 480 System, Hoffmann-La Roche, Indianapolis, IN, USA). Results were normalized against *tubb6* and analysed using the ΔΔCT method (Schmittgen and Livak, 2008). *tubb6* was chosen for standardization as it exhibited the lowest variance across all samples included in the qPCR experiment. One-tailed Student’s t-test was used to determine a significant increase in transcript level at α≤0.05 due to chilling for each gene.

The second experiment comparing *M.* × *giganteus* to *Z. mays* grown side by side in control and chilling conditions used the same protocol. Three additional transcripts were added with known involvement in chilling response in *Z. mays*: *psbA* (D1), *petA* (cytochrome f), and *lhcb4* (chlorophyll a/b-binding protein CP29) (Trzcinska-Danielewicz *et al.*, 2009; Table 1). *Act* was used as the reference gene for normalization of qPCR results because it shows stable expression in both species and has been previously shown to have stable expression during chilling in *Z. mays* (Trzcinska-Danielewicz *et al.*, 2009). Results were normalized against *Act* and analysed using the ΔΔCT method (Schmittgen and Livak, 2008). Student’s t-test was used to determine significant differences at α≤0.05 between treatments for each species and gene.

### Western blot analysis

Total leaf protein was extracted from leaf discs of area 1.27cm^2^ punched from the centre of the lamina of each biological replicate (*n*=4) of each species. Proteins were extracted by grinding frozen samples in individual tubes with stainless steel grinding balls (GBSS 156-5000-01, OPS Diagnostics, Lebanon, NJ, USA) twice for 30 s at 300 strokes min^–1^ (2000 Geno/Grinder, SPEX SamplePrep, Metuchen, NJ, USA). The samples were placed on dry ice and 500 μl extraction buffer added to each tube, as described by Wang *et al.* (2008*b*). Briefly, the extraction buffer contained 50mM HEPES (pH 8.0) 0.05% (v/v) Triton-X 100, 1mM EDTA (pH 8.0), 10mM MgCl_2_, 5.96mM dithiothreitol, 1% (w/v) casein, 1% (w/v) polyvinylpyrrolidone, and one protease inhibitor cocktail tablet per 10ml of extraction buffer (cOmplete ULTRA Tablets, Mini, EDTA-free tablets, Roche Applied Science, Indianapolis, IN, USA). The ground tissue and buffer was homogenized and centrifuged for 1min at 15 000 *g* and then the resulting supernatant was stored at –80 °C until Western blot analysis.

Total leaf protein, on an equal leaf area basis, was loaded onto a 10-lane SDS-PAGE 10% Tris-HCL gel, such that each lane represented the same area of leaf (Mini-PROTEAN TGX 456–1033, Bio-Rad Life Science, Hercules, CA, USA). A leaf area basis, rather than the usual total protein basis, was used so that findings could be related to changes in photosynthetic capacity, which is measured on a leaf area basis (Naidu and Long, 1994; Wang *et al.*, 2008*b*). Polypeptides were separated by SDS-PAGE, as described previously (Wang *et al.*, 2008*b*). Each gel contained four biological replicates for the control and of the chilling treatment for both *M.* × *giganteus* and *Z. mays*. Protein-prestained standards were used to provide a molecular weight ladder (Precision Plus Protein Kaleidoscope Standards 161–0375, Bio-Rad Life Science). Separated proteins were blotted onto polyvinylidene fluoride membranes in transfer buffer (0.025M Tris base, 0.192M glycine, and 20%, v/v, methanol) at 100V for 1h at 4 °C. Membranes were then incubated with primary polyclonal antibodies in Tris-buffered saline (170–6435, Bio-Rad Life Science) with 0.05% Tween 20 (P5927, Sigma-Aldrich, St. Louis, MO, USA). LHCII chlorophyll a/b-binding proteins and the D1 protein and its degradation products were detected with rabbit polyclonal antibodies (AS01-003 and AS05-084, Agrisera Antibodies, Vännäs, Sweden). Membranes were then incubated with anti-rabbit IgG (whole molecule) alkaline phosphatase antibody produced in goat (A7539, Sigma-Aldrich). The secondary antibody was detected with a mixture of 5-bromo-4-chloro-3-indolyl-phosphate and nitroblue tetrazolium, which binds alkaline phosphatase (Western Blue Stabilized Substrate, Promega, Madison, WI, USA). Digital images were made of each membrane and the relative volume and intensity for each band was quantified using the 2D image analysis software ImageQuant TL 7.0 (GE Healthcare Biosciences, Little Chalfont, UK).

Mixed-model analysis of variance followed by least significant difference tests of differences between means determined the significance of the effect of chilling on the content of each of LHCII chlorophyll a/b-binding protein, D1 protein, and D1 degradation products in *M.* × *giganteus* and *Z. mays* (PROC MIXED SAS version 9.2, SAS Institute, Cary, NC, USA). Results were considered significant at α≤0.05 (*n*=4).

## Results

**Fig. 1.**
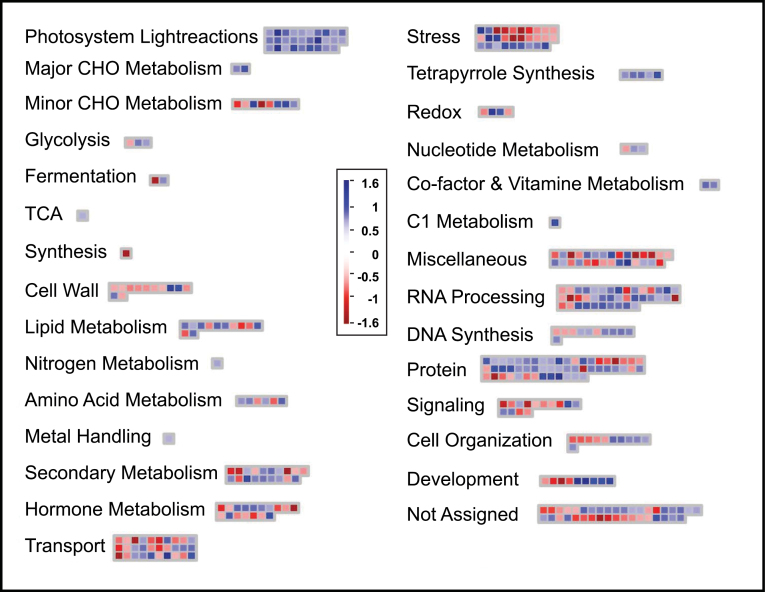
Functional categorization of transcripts found to be up- or downregulated during chilling (≤14 °C) in *Miscanthus* × *giganteus*. Functional groups are as categorized by MapMan (Usadel *et al.* 2009). Red boxes indicate downregulated transcripts and blue boxes represent upregulated transcripts, as indicated by the scale from –1.6 to 1.6 log_2_ fold-change in expression. See Table 2 for numerical details of the change in each transcript.

*M.* × *giganteus* leaf cDNA hybridized to the Agilent 44K maize oligonucelotide microarray with 48% efficiency. Approximately 21 000 probes showed a median fluorescence intensity that could be clearly distinguished from the background. Chilling changed the relative signal intensity for 723 probes relative to the control; based on a significance threshold of a log_2_ fold-change of ≥0.7 and false-discovery-rate-adjusted probability of α<0.00001. These thresholds were selected to include candidate genes with small but reproducible changes in expression (Wurmbach *et al.*, 2003). Chilling caused significant increase in signal intensity for 410 probes and a decrease in 313. Approximately half of the probes showing significant changes corresponded to sequences annotated for predicted or putative functions in *Z. mays*, and these were categorized accordingly in MapMan (Fig. 1). A complete list of putative chilling-responsive genes identified here is given in the supporting information (Supplementary Tables S1 and S2 available at *JXB* online). Functional categories that were significantly represented among chilling-responsive genes included photosynthesis, stress response, RNA processing, protein synthesis, targeting and degradation, transport, secondary metabolism, and hormone metabolism.

**Fig. 2.**
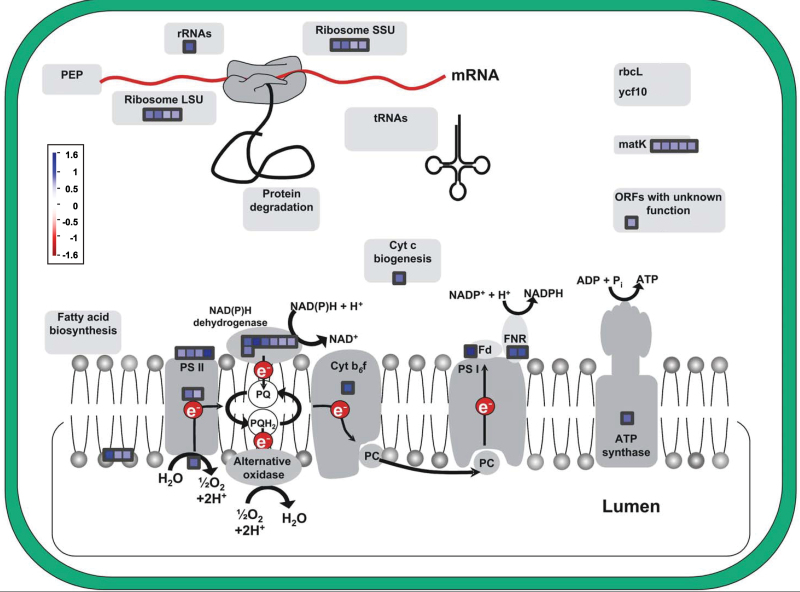
Transcripts related to photosynthesis showing change in *M.* × *giganteus* after 14 days of chilling. Colouration scale as in Fig. 1. See Table 2 for numerical details of the change in each transcript.

Most striking among these categories were genes associated with the light reactions. Among the probes binned by MapMan to this category, 30 were significantly upregulated and none were significantly downregulated (Table 2). Particularly prominent were transcripts for PSII-associated proteins (Fig. 2 and Table 2). Within the carbon metabolism pathway, the probes for PPDK and Rubisco were also maintained and showed high expression values (Supplementary Table S3). Probes for *rca1* and *rca2*, the genes for Rubisco activase, an enzyme that is critical to the activation and stability of Rubisco (Portis, 2003), were upregulated 145%, although just below the fold-change cut off (log_2_ ≤ 0.7).

**Table 2.** Relative changes in abundance of all *M.* × *giganteus* leaf transcripts for chloroplast proteins, associated with the light reactions that showed a log_2_ fold increase ≥0.70 with chilling (14 °C)Data are relative to control (25 °C). There were no significant decreases in response to chilling.

Transcript	Functional category	log_2_ fold-change
Chlorophyll a/b-binding apoprotein CP26 precursor	Photosystem II: light harvesting complex II	0.815
Photosystem II P680 chlorophyll A apoprotein (CP-47 protein)	Photosystem II: light harvesting complex II	0.93
Violaxanthin de-epoxidase precursor	Photosystem II: light harvesting complex II	0.855
D1 protease precursor (fragment)	Photosystem II: light harvesting complex II	1.751
Photosystem II 44kDa reaction centre protein (P6 protein) (CP43)	Photosystem II: reaction centre proteins	0.95
Photosystem II reaction centre J protein	Photosystem II: reaction centre proteins	0.735
Oxygen evolving enhancer protein 3	Photosystem II: oxygen-evolving proteins	0.989
Thylakoid lumenal 17.4kDa protein, chloroplast	Photosystem II: thylakoid lumenal proteins	1.408
Thylakoid lumenal 21.5kDa protein, chloroplast precursor	Photosystem II: thylakoid lumenal proteins	0.805
Thylakoid lumenal 15kDa protein, chloroplast precursor (p15)	Photosystem II: thylakoid lumenal proteins	0.837
NDA2 (ALTERNATIVE NAD(P)H DEHYDROGENASE 2); NADH dehydrogenase	Photosystem II: NAD(P)H dehydrogenase/PQ	0.906
NAD(P)H-quinoneoxidoreductase chain 5, chloroplast (NADH- plastoquinoneoxidoreductase chain 5)	Photosystem II: NAD(P)H dehydrogenase/PQ	1.081
NAD(P)H-quinoneoxidoreductase chain 1, chloroplast (NADH- plastoquinoneoxidoreductase chain 1)	Photosystem II: NAD(P)H dehydrogenase/PQ	1.914
NAD(P)H-quinoneoxidoreductase chain 4L, chloroplast (NADH- plastoquinoneoxidoreductase chain 4L)	Photosystem II: NAD(P)H dehydrogenase/PQ	1.052
NADH-plastoquinoneoxidoreductase subunit 5	Photosystem II: NAD(P)H dehydrogenase/PQ	0.856
NADH-plastoquinoneoxidoreductase subunit 4	Photosystem II: NAD(P)H dehydrogenase/PQ	0.766
NAD(P)H-quinoneoxidoreductase chain 1, chloroplast (NADH- plastoquinoneoxidoreductase chain 1)	Photosystem II: NAD(P)H dehydrogenase/PQ	0.797
Cytochrome b6	Photosystem II: NAD(P)H dehydrogenase/PQ	1.223
3Fe-4S ferredoxin	Photosystem II: NAD(P)H dehydrogenase/PQ	2.734
Ferredoxin-nitrite reductase precursor–maize (fragment) (*Zea mays*)	Photosystem II: NAD(P)H dehydrogenase/PQ	1.153
Ferredoxin-NADP reductase, root isozyme, chloroplast precursor (FNR)	Photosystem II: NAD(P)H dehydrogenase/PQ	1.207
ATP synthase alpha chain	Photosystem light reactions: ATP synthase	1.007
Cytochrome c biogenesis protein family	Chloroplast biogenesis	0.972
Chloroplast ORF70	Chloroplast biogenesis	0.786
matK protein (trnK intron)	Chloroplast proteins	0.8146
*Zea mays* chloroplast rRNA-operon	Chloroplast RNA regulation of transcription	1.078
50S ribosomal protein L29, chloroplast precursor	Chloroplast protein synthesis: plastid ribosomal proteins	0.907
Chloroplast 30S ribosomal protein S18	Chloroplast protein synthesis: plastid ribosomal proteins	0.972
50S ribosomal protein L21, chloroplast precursor (CL21)	Chloroplast protein synthesis: plastid ribosomal proteins	0.721
Chloroplast 50S ribosomal protein L16	Chloroplast protein synthesis: plastid ribosomal proteins	0.775

**Fig. 3.**
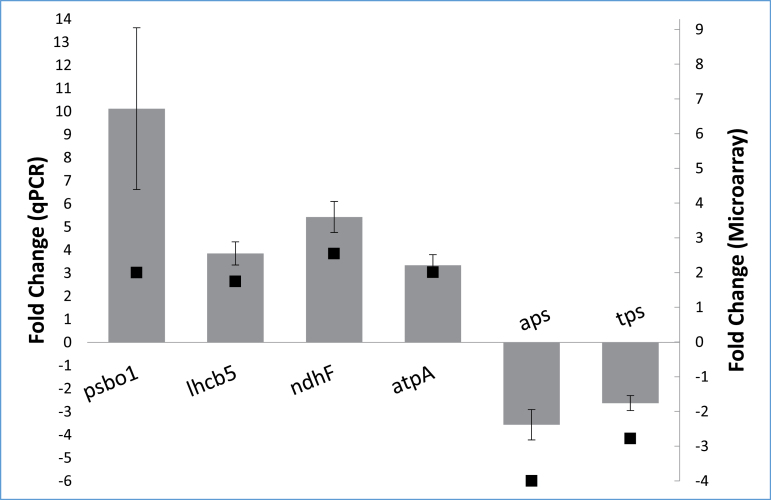
qPCR validation of six transcripts found to be up- or downregulated on the microarray (Table 2): *psb01* (oxygen-evolving enhancer protein 1), *lhcb5* (chlorophyll a/b-binding protein CP26), *ndhF* (NADH dehydrogenase F, chloroplast, *atpA* (ATP synthase alpha subunit), *aps* (ATP sulphurylase), and *tps* (terpene synthase). The ΔΔCT method was used to calculate fold-change and all results were normalized against the reference gene *tubb6*. Grey bars indicate the qPCR-validated fold-change (primary Y-axis) and black squares indicate the microarray fold-change (secondary Y-axis). Error bars represent the standard error of the mean of the biological replicates (*n*=4).

Six of the probes found to change on the microarray were selected for validation by qPCR in the first experiment. Each showed the same directional change as well as similar quantitative changes in expression to that observed from the microarray (α≤0.05; Fig. 3). These included four upregulated probes representing genes encoding key proteins of photosynthetic light reactions: *psbo1* (oxygen-evolving enhancer protein 1), *lhcb5* (chlorophyll a/b-binding protein CP26), *ndhF* (chloroplast NADH dehydrogenase subunit 5), and *atpA* (ATP synthase alpha subunit), as well as two separate downregulated probes, one for *aps* (ATP sulphurylase) and the other for *tps* (terpene synthase). Five probes representing genes with likely housekeeping functions were also tested for constancy of expression between the treatment and control. *tubb6* had the most stable expression among these genes and was therefore used as a reference gene for each of the other seven transcripts in estimating relative fold-changes by the ΔΔCT method (Schmittgen and Livak, 2008).

**Fig. 4.**
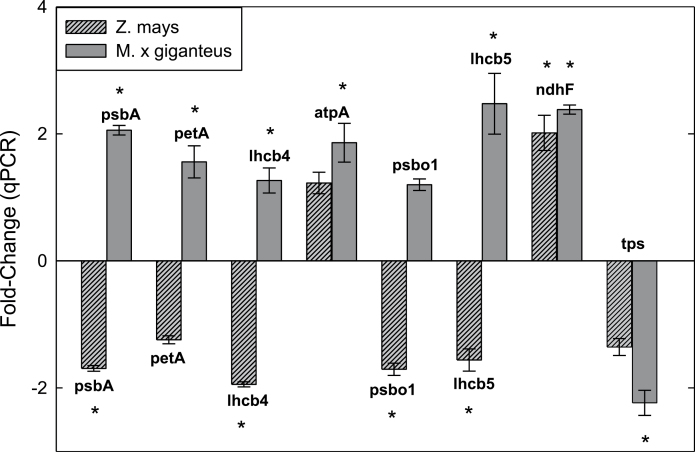
Comparison of gene expression between *M.* × *giganteus* and *Z. mays* after 14 days of chilling (14 °C) versus control (25 °C). qPCR-tested transcripts included five transcripts found to be up- or downregulated on the microarray (*atpA*, ATP synthase alpha subunit; *psbo1*, oxygen-evolving enhancer protein 1; *lhcb5*, chlorophyll a/b-binding protein CP26; *ndhF*, NADH dehydrogenase F; and *tps*, terpene synthase), and three additional light reaction transcripts: *psbA*, D1; *petA*, cytochrome f; and *lhcb4*, chlorophyll a/b-binding protein CP29). Results were normalized against the reference gene *act* and the ΔΔCT method was used to calculate fold-change. Error bars represent the standard error of the mean of all biological replicates (*n*=4). Asterisks indicate significant differential expression between control and chilling (α≤ 0.05).

In a second qPCR experiment comparing *M.* × *giganteus* to *Z. mays*, using five transcripts validated from the microarray experiment and three additional genes with known regulation during chilling, significant differences in gene expression between the two species were found during chilling. Changes in transcripts for *M.* × *giganteus* were consistent with those found in the first experiment, although *psbo1* expression change was not significantly different (*P*=0.14). In *Z. mays*, transcripts for *psbA* (D1), *lhcb4* (chlorophyll a/b-binding protein CP29), *psbo1*, and *lhcb5* all showed significant downregulation (α≤0.05), while the same transcripts, with the exception of *psbo1*, in *M.* × *giganteus* were significantly upregulated (α≤0.05) (Fig. 4). *petA* (cytochrome F) indicated downregulation in *Z. mays* but it was not significantly different from control (*P*=0.14). Similar expression patterns were seen in *ndhF* for both species (Fig. 4). These two qPCR experiments were performed on RNA samples from completely independent chilling treatments and showed the apparent reproducibility of these results in *M.* × *giganteus*.

**Fig. 5.**
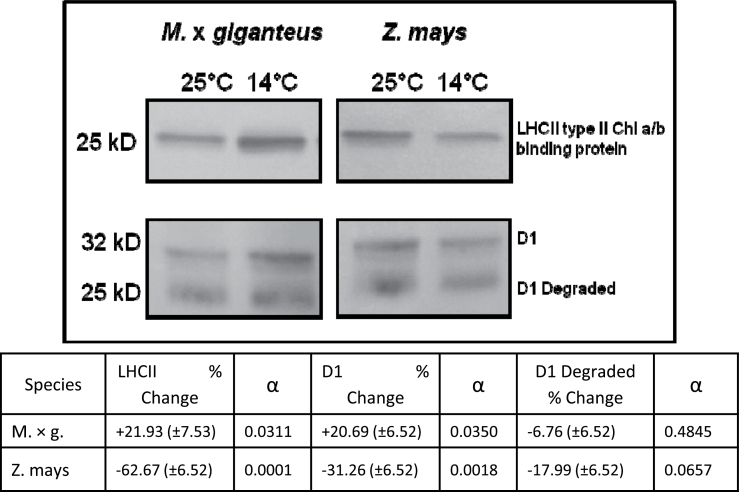
Representative Western blot images showing changes in LHCII type II chlorophyll a/b-binding protein, D1 protein, and D1 degradation products after 14 days of chilling (14 °C) compared to control (25 °C) in *M.* × *giganteus* and *Z. mays*. The same leaves as for Fig. 4 were used. Values are mean±SE for all biological samples. Differences between control and chilling were assessed by Student’s t-test (α≤0.05, *n*=4).

The second experiment also showed that the pattern of change in transcripts was reflected in the amounts of two key proteins. As predicted from the transcript changes, amounts of LHCII type II chlorophyll a/b-binding protein and D1 protein per unit leaf area increased significantly (α≤0.05) in *M.* × *giganteus* leaves in response to chilling, but decreased significantly (α≤0.05) in *Z. mays* (Fig. 5). The proportion of D1 degradation products relative to D1 protein did not change significantly in either species (Fig. 5).

**Table 3.** Comparison of *Miscanthus* × *giganteus* and *Zea mays* transcriptional response to chilling**↑** indicates upregulation during chilling; **↓** indicates downregulation during chilling. Arrows in both directions indicate that some transcripts in this category were upregulated and others downregulated. A larger arrow indicates that more transcripts were up- or downregulated, and vice-versa. Transcriptional data: *M.* × *giganteus*, this study; *Z. mays*, Trzcinska-Danielewicz *et al.* (2009).

Category	*M. × giganteus*		*Z. mays*	
	Response	No. of genes	Response	No. of genes
Protein synthesis	↑↓	8	↑↓	8
Protein degradation	↑↓	17	↑	1
Chlorophyll biosynthesis	↑	3	↓	1
PS II reaction centre and LHCII	↑	6	↓	3
Temperature Induced Proteins	↓	1	–	0
Cellulose synthesis	↓	2	↓	1
Flavonol biosynthesis	↑↓	5	–	0
Brassinosteroid biosynthesis	↓	1	–	0
Ethylene	↓	1	↓	3
Gibberellic acid biosynthesis	↓	3	–	0
Abscisic acid biosynthesis	↓↑	3	↓	1
Cytokinins	↓	1	–	0
Wax biosynthesis	↑↓	2	–	0

## Discussion

The present study asked whether the physiological chilling tolerance of photosynthesis in *M.* × *giganteus* corresponded to maintenance or upregulation of genes encoding key aspects of the photosynthetic apparatus, specifically those proteins shown to be damaged or deficient in *Z. mays* during acclimation to chilling that relate to energy transduction on the photosynthetic membrane. Decline in the maximum quantum yield of *ΦCO*_2max_ in maize with chilling has been associated with the loss of key proteins of the photosynthetic membrane, particularly those of PSII and those involved in transfer of excitation energy to PSII (Fryer *et al.*, 1995). In contrast, a small but not statistically significant loss of *ΦCO*_2max_ was indicated when *M.* × *giganteus* was subjected to the same chilling conditions (Naidu and Long, 2004). This raises the question of how this species avoids this loss. Here, 30 genes annotated as encoding proteins with key functions in the light reactions of photosynthesis showed significantly increased expression, and none of this category showed a decrease. This suggests that *M.* × *giganteus* may avoid the losses observed in maize, at least in part, by increasing synthesis of these key proteins. Across all of the MapMan categories, photosynthesis was the only one that did not show any downregulated probes (Tables 2 and 3 and Fig. 2). Functions of the proteins encoded by upregulated transcripts include electron transport, light harvesting, photosystem reaction centres, ATP synthesis, and the xanthophyll cycle which protects PSII against photoinhibitory damage (Demmig-Adams and Adams, 1992; Fryer *et al.*, 1995; Farage *et al.*, 2006). In the current study, in *Z. mays* following 28h of 14 °C chilling, four genes associated with the light reactions of photosynthesis were significantly downregulated. These encode the chlorophyll a/b-binding protein precursor, chlorophyll a/b-binding apoprotein and minor antenna complex CP24 precursor, chlorophyll a/b-binding and minor antenna complex CP26, and NAD kinase chloroplast precursor (Trzcinska-Danielewicz *et al.*, 2009). The current study, which used a longer chilling treatment, similarly showed that genes encoding key proteins of the photosynthetic light reactions (i.e. D1, cytochrome f, PSII CP29, oxygen-evolving enhance protein 1, and chlorophyll a/b-binding proteins) were significantly downregulated in maize but upregulated in *M.* × *giganteus* (Fig. 4).

Parallel to these transcript studies, proteomic analysis of *Z. mays* leaves developed at 13 °C show large decreases in the minor antenna complexes CP24, CP26, and CP29 (Caffarri *et al.*, 2005). In a similar experiment, Nie and Baker (1991) showed large decreases at 12 °C also in the 26-kDa LHCII apoprotein, as well as the 32-kDa D1 reaction centre protein, cytochrome f (33kDa), cytochrome b6/f subunit IV (17kDa), and the α and β subunits of the coupling factor (58 and 57kDa). Consistent with these prior studies, Western blotting in the current study similarly showed significant decreases in both D1 reaction centre protein and LHCII chlorophyll a/b-binding protein in *Z. mays* after 14 days of chilling. In contrast, these same proteins increased in *M.* × *giganteus* (Fig. 5). For plants that acclimate to chilling conditions, increased overall leaf protein contents are commonly observed. This study group has previously shown that acclimation to the chilling conditions used here lowers total protein per unit leaf area by 17% in *Z. mays* and increases content by 2% in *M.* × *giganteus* (Naidu *et al.*, 2003). This suggests that the much larger increases observed here in LHCII chlorophyll a/b-binding protein and D1 are more than the result of an overall increase in protein content. This difference in levels of these light reaction-related transcripts and protein contents is likely to be a key underlying mechanism, explaining an unusual but effective acclimation of photosynthesis to chilling.

A critical function necessary to the maintenance of photosynthesis during chilling in C_3_ and C_4_ plants is the ability of the chloroplast to degrade damaged D1, a component of PSII, and to then synthesize and assemble D1 back into PSII (Bredenkamp and Baker, 1994; Fryer *et al.*, 1995; Ruelland *et al.*, 2009). This repair function is inhibited in *Z. mays* during chilling and this has been attributed to decreased expression of *psbA*, the plastid gene that encodes D1 (Bredenkamp and Baker, 1994; Allen and Ort, 2001). The current results show that the transcripts for the gene for D1 protease, involved in the degradation of damaged D1, and for *psbA*, the gene encoding D1, are strongly upregulated in *M.* × *giganteus* grown under chilling conditions, with fold-changes of 3.37 (Table 2) and 2.06 (Fig. 4), respectively. Damage to D1 is a major cause of loss of PSII efficiency, since absorbed light energy will be channelled to inactive centres that, under light-limiting conditions, will lower *ΦCO*_2max_ (reviewed by Long *et al.*, 1994). Previously a high expression of xanthophyll cycle pigments and accumulation of zeaxanthin during chilling of *M.* × *giganteus* has been shown (Farage *et al.*, 2006). The present study provides an explanation for this: a significant increase in expression of the transcript for a violaxanthin de-epoxidase, which converts violaxanthin to zeaxanthin (log_2_ fold-change 0.855, Table 2). Accumulation of zeaxanthin would serve to lessen damage to D1 by dissipating excess excitation energy by thermal de-excitation (Farage *et al.*, 2006). This, combined with increased capacity for processing damaged D1 should greatly decrease the number of centres with damaged D1 at any point in time and could explain the maintenance of high *ΦCO*_2max_ during chilling, as observed in *M.* × *giganteus* in chilling in contrast to maize (Naidu and Long, 1994; Beale *et al.*, 1996).

Maximum quantum yield (*ΦCO*_2max_) and light-saturated rates (*A*_sat_) of carbon assimilation are equally important in maintaining crop canopy carbon gain over a diurnal course, since this depends on both sunlit and shaded canopy leaves (Baker *et al.*, 1988). While *ΦCO*_2max_ in C_4_ photosynthesis depends on the efficiency of energy transduction on the photosynthetic membrane and the stability of key proteins, such as D1, *A*_sat_ depends on the maximum capacity of the rate-limiting steps in electron flow and carbon metabolism beyond the reaction centres, and diversion of electrons to sinks other than CO_2_ assimilation. During chilling, *M.* × *giganteus*, in sharp contrast to *Z. mays*, maintains high levels of the enzymes of photosynthetic carbon metabolism that exert maximal metabolic control of *A*_sat_, Rubisco, and PPDK. Rubisco protein contents are stable in *M.* × *giganteus* in response to chilling while they decline by more than 50% in *Z. mays* during chilling (Naidu *et al.*, 2003; Wang *et al.*, 2008*a*). PPDK declines to an even greater extent in *Z. mays* but increases in *M.* × *giganteus* (Naidu *et al.*, 2003; Wang *et al.*, 2008*b*). The current results correspond with these findings, with the expression levels of both Rubisco (log_2_ fold-change 0.142) and PPDK (log_2_ fold-change 0.015) remaining stable during chilling. In addition, the transcripts for the two isoforms of Rubisco activase (*rca1* and *rca2*) were upregulated during chilling (Supplementary Table S3). Rubisco activase is a catalytic chaperone that is critical to maintaining the stability and high catalytic activity of Rubisco. Specifically, Rubisco activase promotes the dissociation of a wide variety of inhibitory sugar phosphates from the Rubisco active site (Portis, 2003). More recently, Shan *et al.* (2011) found that downregulation of *rca* was a key and necessary component in the jasmonate-induced senescence that is associated with a wide range of abiotic and biotic stresses. It is intriguing that chilling in *M.* × *giganteus* induced the opposite response to that seen in induced senescence.

The Agilent 44K maize oligonucleotide microarray chip is a platform that was designed to include nearly all available maize genes (Skibbe and Walbot, 2009). It includes expressed sequence tags involved in flowering, fruit formation, root and shoot development, and germination, as well as developing and mature leaves (Skibbe and Walbot, 2009). A prior study utilizing this platform found that unstressed mature *Z. mays* leaves expressed approximately 26 000 transcripts under greenhouse growth conditions (Casati and Walbot, 2008). This indicates that approximately 59% of transcripts on the array are expressed in adult *Z. mays* leaves. A study on the diurnal rhythms of the maize leaf trancriptome, using a 105K Agilent maize array designed for all maize genomic and transcript sequences, found that approximately 44 000 transcripts were detectable in the tested maize leaves, equating to 42% of the total transcripts (Hayes *et al.*, 2010). Since the majority of transcripts associated with photosynthesis are maximally expressed during daylight hours (Urbanczyk-Wochniak *et al.*, 2005), samples were taken in this study 2–3h into the photoperiod. At this time point, 48% of the probes were expressed in *M.* × *giganteus* leaves in the control (25 °C) and chilling (14 °C). This is comparable to other studies using Agilent maize arrays. This hybridization efficiency for *M.* × *giganteus* leaf mRNA to the maize array is consistent with the >90% sequence identity observed among exon sequences between *M.* × *giganteus* and *Z. mays* (Swaminathan *et al.*, 2010). This is not surprising given that both are members of the same grass tribe, Andropogoneae. This grass tribe is exclusively of the NADP-ME C_4_ subtype (Giussani *et al.*, 2001), suggesting that C_4_ photosynthesis in maize, *Miscanthus*, and other species of the tribe most likely evolved from a single common ancestor (Sage, 2004; Swaminathan *et al.*, 2010).

While *Z. mays* is a crop of tropical origin, more than 60% of global production today is in the temperate zone (Long and Spence, 2013). It is therefore grown in colder climates more than any other C_4_ food crop, yet its inability to acclimate photosynthesis to chilling conditions is an Achilles heel (Baker *et al.*, 1989; Dohleman *et al.*, 2009). It is assumed that C_4_ photosynthesis evolved in hot climates and subsequently radiated to more temperate climates. The photosynthetic genes identified as upregulated in chilling-tolerant *M.* × *giganteus* in this comparative study indicate targets for upregulation, by selection or bioengineering, to improve the chilling tolerance of maize and to contribute to allowing a longer growing season for the crop in temperate climates.

## Supplementary material

Supplementary data are available at *JXB* online.

Supplementary Table S1. All transcripts of *M.* × *giganteus* leaves found to be significantly upregulated by treatment with a log_2_ fold-change ≥0.70.

Supplementary Table S2. All transcripts of *M.* × *giganteus* leaves found to be significantly downregulated by treatment with a log_2_ fold-change ≥0.70.

Supplementary Table S3. Transcripts for Rubisco, Rubisco-interacting proteins, and PPDK transcripts showing no significant log_2_ fold-change and maintaining high expression during chilling.

Supplementary Data
