# Supplementary material for: Transcriptional responses indicate maintenance of photosynthetic proteins as key to the exceptional chilling tolerance of C4 photosynthesis in Miscanthus × giganteus
Source: J Exp Bot. 2014 Jun 22;65(13):3737–47. doi: 10.1093/jxb/eru209 (PMC4085969; doi:10.1093/jxb/eru209)
Supplement: Supplementary Data [file supp_65_13_3737__index.html]

Transcriptional responses indicate maintenance of photosynthetic proteins as key to the exceptional chilling tolerance of C4 photosynthesis in Miscanthus × giganteus — Transcriptional responses indicate maintenance of photosynthetic proteins as key to the exceptional chilling tolerance of C4 photosynthesis in Miscanthus × giganteus — Supplementary Data 

# Transcriptional responses indicate maintenance of photosynthetic proteins as key to the exceptional chilling tolerance of C4 photosynthesis in *Miscanthus* × *giganteus*

## Supplementary Data

Data files

**Files in this Data Supplement:**

- Supplementary Data - Supplementary Data
