# Supplementary material for: Transcriptional responses indicate maintenance of photosynthetic proteins as key to the exceptional chilling tolerance of C4 photosynthesis in Miscanthus × giganteus
Source: J Exp Bot. 2014 Jun 22;65(13):3737–47. doi: 10.1093/jxb/eru209 (PMC4085969; doi:10.1093/jxb/eru209)
Supplement: Supplementary Data [file supp_eru209_jexbot113928_file001.docx]

Supplementary Table 1. All transcripts found to be significantly up-regulated with a log_2_ fold-change ≥ 0.70 with the chilling treatment (adj. p-value .00001). Annotations include the Agilent probe ID, MapMan bincode and corresponding bin name, and gene description.

| ID | BINCODE | BIN NAME | GENE DESCRIPTION | | LogFC |
| --- | --- | --- | --- | --- | --- |
|  |  |  |  |  | |
|  |  |  |  |  | |
| **Protein** |  |  |  |  | |
|  |  |  |  |  | |
| A_92_P022015 | 29.6 | protein.folding | 760 similar to UP:FKBP1_ARATH (Q9LM71) Probable FKBP-type peptidyl-prolyl cis-trans isomerase 1, chloroplast precursor (PPIase) (Rotamase) , partial (53%) | 1.71 | |
| A_92_P014196 | 29.2.2 | protein.synthesis.misc ribososomal protein | UP\|Q5SBH9 MAIZE (Q5SBH9) Ribosomal protein S13, complete | 1.42 | |
| A_92_P026815 | 29.5.11.04.03.02 | protein.degradation.ubiquitin.E3.SCF.FBOX | 1392 similar to UP:Q9M648_ARATH (Q9M648) FKF1 (Adagio 3), partial (56%) | 1.40 | |
| A_92_P015985 | 29.5.11.04.03.02 | protein.degradation.ubiquitin.E3.SCF.FBOX | ACG28353 tubby-like protein [Zea mays] | 1.39 | |
| A_92_P031433 | 29.4 | protein.postranslational modification | 1363 homologue to UP:SAPK3_ORYSA (Q75V63) Serine:threonine-protein kinase SAPK3 (Osmotic stress:abscisic acid-activated protein kinase 3) (Protein kinase REK) , partial (98%) | 1.35 | |
| A_92_P025065 | 29.4 | protein.postranslational modification | 1344 similar to GB:BAC05575.1:21902025:AP003332 protein phosphatase 2C-like protein (Oryza sativa (japonica cultivar-group)), partial (22%) | 1.34 | |
| A_92_P012825 | 29.4 | protein.postranslational modification | 660 similar to PRF:NP_568174.1:18415301:NP_568174 protein phosphatase 2C family protein : PP2C family protein (Arabidopsis thaliana), partial (31%) | 1.32 | |
| A_92_P032260 | 29.3.99 | protein.targeting.unknown | 823 similar to UP:Q8W156_BRAOL (Q8W156) Deoxycytidine deaminase, partial (96%) | 1.16 | |
| A_92_P013317 | 29.1 | protein.aa activation | 1778 similar to UP:Q9LJE2_ARATH (Q9LJE2) Lysyl-tRNA synthetase, partial (72%) | 1.14 | |
| A_92_P014013 | 29.2.1.99 | protein.synthesis.chloroplast/mito - plastid ribosomal protein.unknown | 1968 UP:RR18_MAIZE (P25459) Chloroplast 30S ribosomal protein S18, complete | 0.97 | |
| A_92_P033249 | 29.5 | protein.degradation | 632 similar to UP:Q8RY11_ARATH (Q8RY11) AT3g05350:T12H1_32, partial (20%), aminopeptidase | 0.96 | |
| A_92_P020018 | 29.5 | protein.degradation | 862 weakly similar to UP:CBPY_SCHPO (O13849) Carboxypeptidase Y precursor (CPY) , partial (6%) | 0.96 | |
| A_92_P004906 | 29.4 | protein.postranslational modification | 711 similar to PRF:NP_568174.1:18415301:NP_568174 protein phosphatase 2C family protein : PP2C family protein (Arabidopsis thaliana), partial (33%) | 0.95 | |
| A_92_P018510 | 29.5 | protein.degradaton | 780 similar to UP:Q8RY11_ARATH (Q8RY11) AT3g05350:T12H1_32, partial (16%) | 0.93 | |
| A_92_P034180 | 29.5.11 | protein.degradation.ubiquitin | 1466 weakly similar to UP:Q8VWY0_CUCSA (Q8VWY0) Ubiquitin-like protein, partial (87%) | 0.91 | |
| A_92_P020783 | 29.3.99 | protein.targeting.unknown | 982 similar to UP:Q8W156_BRAOL (Q8W156) Deoxycytidine deaminase, partial (96%) | 0.91 | |
| A_92_P019516 | 29.2.1.99 | protein.synthesis.chloroplast/mito - plastid ribosomal protein.unknown | 811 similar to UP:RK29_MAIZE (Q9SWI6) 50S ribosomal protein L29, chloroplast precursor, complete | 0.91 | |
| A_92_P021629 | 29.5.03 | protein.degradation.cysteine protease | 1069 similar to UP:Q6H7E6_ORYSA (Q6H7E6) PRLI-interacting factor N-like, partial (39%) | 0.88 | |
| A_92_P026007 | 29.4 | protein.postranslational modification | 1738 similar to PRF:NP_195770.1:15240999:NP_195770 mitochondrial substrate carrier family protein (Arabidopsis thaliana), partial (71%) | 0.85 | |
| A_92_P006260 | 29.4 | protein.postranslational modification | 1869 similar to UP:SPR1_YEAST (P32603) Sporulation-specific glucan 1,3-beta-glucosidase precursor (Exo-1,3-beta-glucanase) , partial (4%) | 0.85 | |
| A_92_P010355 | 29.4 | protein.postranslational modification | 744 similar to UP:Q6L5C4_ORYSA (Q6L5C4) Protein phosphatase 2C, partial (34%) | 0.85 | |
| A_92_P000461 | 29.2.3 | protein.synthesis.initiation | 943 similar to PRF:NP_566333.1:18398225:NP_566333 elongation factor P (EF-P) family protein (Arabidopsis thaliana), partial (54%) | 0.85 | |
| A_92_P031429 | 29.4 | protein.postranslational modification | 1500 similar to UP:Q84VY2_ARATH (Q84VY2) At2g30500, partial (17%) | 0.84 | |
| A_92_P041139 | 29.2.1.1 | protein.synthesis.chloroplast/mito - plastid ribosomal protein.plastid | NP_200203 ribosomal protein L11 methyltransferase-related [Arabidopsis thaliana] | 0.79 | |
| A_92_P029287 | 29.2.1.99 | protein.synthesis.chloroplast/mito - plastid ribosomal protein.plastid | 2906 UP:RK16_MAIZE (P08528) Chloroplast 50S ribosomal protein L16, partial (98%) | 0.77 | |
| A_92_P018384 | 29.2.1.1 | protein.synthesis.chloroplast/mito - plastid ribosomal protein.plastid | NP_193746 PTAC14 (PLASTID TRANSCRIPTIONALLY ACTIVE14) [Arabidopsis thaliana] | 0.76 | |
| A_92_P026877 | 29.4 | protein.postranslational modification | 687 weakly similar to PRF:NP_680566.2:42570054:NP_680566 mitochondrial substrate carrier family protein (Arabidopsis thaliana), partial (23%) | 0.75 | |
| A_92_P028460 | 29.5.03 | protein.degradation.cysteine protease | 800 similar to PIR:F96767:F96767 proteinase IV F2P9.14 [imported] (Arabidopsis thaliana), partial (26%) | 0.74 | |
| A_92_P025987 | 29.5.11.04.02 | protein.degradation.ubiquitin.E3.RING | 2401 homologue to UP:Q8VX27_MAIZE (Q8VX27) Inwardly rectifying potassium channel, complete | 0.74 | |
| A_92_P031457 | 29.6 | protein.folding | 2214 UP:CH62_MAIZE (Q43298) Chaperonin CPN60-2, mitochondrial precursor (HSP60-2), complete | 0.74 | |
| A_92_P038554 | 29.6 | protein.folding | 1220 similar to UP:FKBP2_ARATH (O22870) Probable FKBP-type peptidyl-prolyl cis-trans isomerase 2, chloroplast precursor (PPIase) (Rotamase) , partial (66%) | 0.74 | |
| A_92_P004781 | 29.4 | protein.postranslational modification | 1676 similar to GB:BAB64200.1:15408804:AP003266 serine:threonine protein kinase-like protein (Oryza sativa (japonica cultivar-group)), partial (87%) | 0.72 | |
| A_92_P008135 | 29.2.1.99 | protein.synthesis.chloroplast/mito - plastid ribosomal protein.plastid | 963 similar to UP:RK21_ARATH (P51412) 50S ribosomal protein L21, chloroplast precursor (CL21), partial (51%) | 0.72 | |
| A_92_P015141 | 29.6 | protein.folding | 837 similar to GB:AAO64777.1:29028796:BT005842 At3g60210 (Arabidopsis thaliana), partial (65%), chloroplast chaperonin 10 | 0.72 | |
| A_92_P031646 | 29.1 | protein.aa activation | 1919 UP:O82110_MAIZE (O82110) Seryl-tRNA synthetase (Fragment) , complete | 0.72 | |
| A_92_P019676 | 29.4 | protein.postranslational modification | 1553 similar to PRF:NP_193883.2:30685465:NP_193883 transducin family protein : WD-40 repeat family protein (Arabidopsis thaliana), partial (59%) | 0.71 | |
| A_92_P025466 | 29.2.1.1 | protein.synthesis.chloroplast/mito - plastid ribosomal protein.unknown | 1607 similar to GB:AAM65771.1:21593804:AY088230 ribosomal protein S1 (Arabidopsis thaliana), partial (77%) | 0.70 | |
|  |  |  |  |  | |
|  |  |  |  |  | |
| **Regulation of Transcription** | | |  |  | |
|  |  |  |  |  | |
| A_92_P027487 | 27.3.24 | RNA.regulation of transcription.MADS box transcription factor family | NP_001104927 MADS box protein 3 [Zea mays] | 1.40 | |
| A_92_P026770 | 27.2 | RNA.transcription | 2107 UP:Q8RWR8_MAIZE (Q8RWR8) Sigma factor protein, complete | 1.38 | |
| A_92_P040597 | 27.3.99 | RNA.regulation of transcription.unclassified | gb\|U42796.1\|ZMU42796 Zea mays 18S ribosomal RNA gene, partial sequence, partial (19% | 1.26 | |
| A_92_P015185 | 27.3.99 | RNA.regulation of transcription.unclassified | 921 similar to GB:BAD38854.1:51571875:AB189038 pseudo-response regulator 1 (Oryza sativa (japonica cultivar-group)), partial (42%) | 1.18 | |
| A_92_P021467 | 27.3.67 | RNA.regulation of transcription.putative DNA-binding protein | ACG26686 LHY protein [Zea mays] late elongated hypocotyl possible affects circadian clock- flowering independent of photoperiod DNA binding | 1.17 | |
| A_92_P017343 | 27.3.99 | RNA.regulation of transcription.unclassified | ACG43211 two-component response regulator-like PRR95 [Zea mays] | 1.15 | |
| A_92_P004858 | 27.3.67 | RNA.regulation of transcription.putative DNA-binding protein | 1651 UP:RPOA_MAIZE (P09562) DNA-directed RNA polymerase alpha chain (PEP) (Plastid-encoded RNA polymerase alpha subunit) (RNA polymerase alpha subunit) , complete | 1.09 | |
| A_92_P022439 | 27.3.57 | RNA.regulation of transcription.JUMONJI family | 1605 similar to UP:Q6YVS8_ORYSA (Q6YVS8) N-acetyltransferase and Transcription factor-like protein, partial (85%) | 1.09 | |
| A_92_P024541 | 27.3.99.01 | RNA.regulation of transcription.chloroplast | emb\|Z00028.1\|CHZMRRNA Zea mays chloroplast rRNA-operon, partial (11%) | 1.08 | |
| A_92_P014527 | 27.3.99 | RNA.regulation of transcription.unclassified | 893 homologue to UP:PRR1_ORYSA (Q689G9) Two-component response regulator-like PRR1 (Pseudo-response regulator 1) (OsPRR1), partial (25%) | 1.04 | |
| A_92_P000917 | 27.3.55 | RNA.regulation of transcription.HDA | similar to UP\|Q30DN4 ORYSA (Q30DN4) Hd1, partial (26%) | 1.00 | |
| A_92_P022411 | 27.3.99 | RNA.regulation of transcription.unclassified | 1245 weakly similar to PRF:NP_974703.1:42573213:NP_974703 mitochondrial transcription termination factor-related : mTERF-related (Arabidopsis thaliana), partial (43%) | 0.97 | |
| A_92_P021253 | 27.3.99 | RNA.regulation of transcription.unclassified | 1109 similar to PRF:NP_973859.1:42571537:NP_973859 zinc finger (C3HC4-type RING finger) family protein (Arabidopsis thaliana), partial (39%) | 0.96 | |
| A_92_P040904 | 27.3.12 | RNA.regulation of transcription.C3H zinc finger family | 1870 similar to UP:Q9FU27_ORYSA (Q9FU27) CCCH-type zinc finger protein-like, partial (73%) | 0.93 | |
| A_92_P006302 | 27.3.69 | RNA.regulation of transcription.SET-domain transcriptional regulator family | 961 similar to UP:Q6STH5_ARATH (Q6STH5) [4Fe-4S] cluster assembly factor, partial (48%) | 0.92 | |
| A_92_P032122 | 27.3.07 | RNA.regulation of transcription.C2C2(Zn) CO-like, Constans-like zinc finger family | ACG35514 zinc finger protein CONSTANS-LIKE 16 [Zea mays] | 0.89 | |
| A_92_P003160 | 27.3.69 | RNA.regulation of transcription.SET-domain transcriptional regulator family | 901 homologue to UP:Q7XHS1_ORYSA (Q7XHS1) 2Fe-2S iron-sulfur cluster protein-like, partial (60%) | 0.77 | |
| A_92_P001197 | 27.3.65 | RNA.regulation of transcription.Polycomb Group (PcG) | 782 similar to GB:CAD35362.1:21535744:ATH490171 FK506 binding protein 1 (Arabidopsis thaliana), partial (48%) | 0.77 | |
| A_92_P031312 | 27.3.44 | RNA.regulation of transcription.Chromatin Remodeling Factors | BAC84084 putative SNF2 domain/helicase domain-containing protein [Oryza sativa Japonica Group] | 0.74 | |
| A_92_P009406 | 27.3.25 | RNA.regulation of transcription.MYB domain transcription factor family | 818 similar to PRF:NP_027544.1:18395562:NP_027544 myb family transcription factor (Arabidopsis thaliana), partial (14%) | 0.73 | |
| A_92_P041279 | 27.3.69 | RNA.regulation of transcription.SET-domain transcriptional regulator family | 2436 UP:Q8L821_MAIZE (Q8L821) SET domain-containing protein SET118, complete | 0.71 | |
|  |  |  |  |  | |
|  |  |  |  |  | |
| **RNA Processing** | |  |  |  | |
|  |  |  |  |  | |
| A_92_P041988 | 27.1 | RNA.processing | ACG27691 ribonucleoprotein [Zea mays] | 0.99 | |
| A_92_P025875 | 27.1.1 | RNA.processing.splicing | 1536 similar to UP:O81126_ARATH (O81126) 9G8-like SR protein (RSZp22 splicing factor), partial (69%) | 0.94 | |
| A_92_P030730 | 27.1 | RNA.processing | 2076 similar to UP:Q6NQ85_ARATH (Q6NQ85) At4g09730 (MRNA, complete cds, clone: RAFL21-78-P15) (MRNA, complete cds, clone: RAFL21-86-L24) (MRNA, complete cds, clone: RAFL22-33-K01), partial (68%) | 0.93 | |
| A_92_P025198 | 27.1 | RNA.processing | 2067 similar to UP:Q6L724_HORVU (Q6L724) ATP-dependent RNA helicase, partial (66%) | 0.76 | |
| A_92_P002168 | 27.1 | RNA.processing | 804 UP:Q64HC3_MAIZE (Q64HC3) ASF:SF2-like pre-mRNA splicing factor SRP32, partial (18%) | 0.73 | |
|  |  |  |  |  | |
|  |  |  |  |  | |
| **TCA** |  |  |  |  | |
|  |  |  |  |  | |
| A_92_P033332 | 8.1.01.02 | TCA / org. transformation.TCA.pyruvate DH.E2 | 1771 similar to UP:Q9LUA6_ARATH (Q9LUA6) Dihydrolipoamide S-acetyltransferase, partial (73%) | 0.72 | |
|  |  |  |  |  | |
|  |  |  |  |  | |
| **Transport** |  |  |  |  | |
|  |  |  |  |  | |
| A_92_P031545 | 34.19.2 | transport.Major Intrinsic Proteins.TIP | 1305 homologue to UP:Q9ATL5_MAIZE (Q9ATL5) Tonoplast membrane integral protein ZmTIP4-2, complete | 1.67 | |
| A_92_P014071 | 34.99 | transport misc | 1447 similar to UP:Q9M5P1_ORYSA (Q9M5P1) Secretory carrier membrane protein, partial (96%) | 1.22 | |
| A_92_P004977 | 34.18 | transport.unspecified anions | similar to UP\|Q2QS63 ORYSA (Q2QS63) CLC-d chloride channel; anion channel protein, partial (18%) | 1.20 | |
| A_92_P000665 | 34.7 | transport.phosphate | 492 similar to PRF:NP_197538.2:42567984:NP_197538 transporter-related (Arabidopsis thaliana), partial (11%) | 1.05 | |
| A_92_P041862 | 34.15 | transport.potassium | 2373 similar to UP:Q9LQ76_ARATH (Q9LQ76) T1N6.21 protein, partial (96%), K efflux antiporter 1 | 0.97 | |
| A_92_P031659 | 34.12 | transport.metal | ACG39262 zinc transporter 4 [Zea mays] | 0.96 | |
| A_92_P003712 | 34.15 | transport.potassium | 955 similar to UP:Q9LQ76_ARATH (Q9LQ76) T1N6.21 protein, partial (32%) | 0.94 | |
| A_92_P024704 | 34.16 | transport.ABC transporters and multidrug resistance systems | 770 similar to UP:Q9CH68_LACLA (Q9CH68) ABC transporter permease protein, partial (4%) | 0.89 | |
| A_92_P025776 | 34.15 | transport.potassium | XP_002320781 potassium efflux antiporter [Populus trichocarpa] | 0.88 | |
| A_92_P026571 | 34.16 | transport.ABC transporters and multidrug resistance systems | ABA94465 ABC transporter, putative, expressed [Oryza sativa (japonica cultivar-group)] | 0.83 | |
| A_92_P034631 | 34.12 | transport.metal | 836 similar to UP:Q94IM5_HORVU (Q94IM5) P-type ATPase (Fragment), partial (44%) | 0.80 | |
| A_92_P023563 | 34.9 | transport.metabolite transporters at the mitochondrial membrane | 1602 similar to UP:Q6YVE7_ORYSA (Q6YVE7) Mitochondrial aspartate-glutamate carrier protein-like, partial (97%) | 0.79 | |
| A_92_P009918 | 34.3 | transport.amino acids | 2024 similar to PRF:NP_850361.1:30688867:NP_850361 amino acid transporter family protein (Arabidopsis thaliana), partial (79%) | 0.79 | |
|  |  |  |  |  | |
|  |  |  |  |  | |
| **Signalling** |  |  |  |  | |
|  |  |  |  |  | |
| A_92_P010280 | 30.5 | signalling.G-proteins | weakly similar to UP\|ROGF2 ARATH (Q9LQ89) Rop guanine nucleotide exchange factor 2 (RopGEF2), partial (38%) | 1.30 | |
| A_92_P039544 | 30.5 | signalling.G-proteins | 1323 homologue to UP:Q68HC1_WHEAT (Q68HC1) Rab GTP-binding protein, complete | 0.94 | |
| A_92_P020329 | 30.5 | signalling.G-proteins | 1445 homologue to UP:Q68HC1_WHEAT (Q68HC1) Rab GTP-binding protein, complete | 0.92 | |
| A_92_P035301 | 30.5 | signalling.G-proteins | 1534 similar to UP:Q7EZC9_ORYSA (Q7EZC9) Nucleolar GTP-binding protein 1-like, partial (80%) | 0.87 | |
| A_92_P030885 | 30.2.3 | signalling.receptor kinases.leucine rich repeat III | 2422 UP:O81105_MAIZE (O81105) Leucine-rich repeat transmembrane protein kinase 1 (Fragment), complete | 0.79 | |
|  |  |  |  |  | |
|  |  |  |  |  | |
| **DNA Processing** | |  |  |  | |
|  |  |  |  |  | |
| A_92_P038437 | 28.2 | DNA.repair | 1371 similar to UP:Q9LJK7_ARATH (Q9LJK7) DNA repair protein RAD54-like, partial (25%) | 0.95 | |
| A_92_P034364 | 28.2 | DNA.repair | 1511 UP:R51A1_MAIZE (Q67EU8) DNA repair protein RAD51 homolog A (Rad51-like protein A) (RAD51A) (ZmRAD51a), complete | 0.92 | |
| A_92_P038646 | 28.1.1 | DNA.synthesis/chromatin structure.retrotransposon/transposase | ABA93737 retrotransposon protein, putative, Ty1-copia subclass [Oryza sativa (japonica cultivar-group)] | 0.90 | |
| A_92_P008754 | 28.2 | DNA.repair | 1375 weakly similar to UP:Q5H794_ARATH (Q5H794) AtRAD51Balpha protein, partial (62%) | 0.90 | |
| A_92_P010166 | 28.99 | DNA.unspecified | 1404 similar to PRF:NP_566318.1:18398046:NP_566318 3 exoribonuclease family domain 1-containing protein (Arabidopsis thaliana), complete | 0.88 | |
| A_92_P033224 | 28.1 | DNA.synthesis/chromatin structure | 989 similar to UP:Q9FT74_ARATH (Q9FT74) DNA Helicase, partial (38%) | 0.77 | |
| A_92_P040510 | 28.1 | DNA.synthesis/chromatin structure | 719 similar to UP:Q6UEJ2_PEA (Q6UEJ2) Mini-chromosome maintenance 7, partial (17%) | 0.76 | |
|  |  |  |  |  | |
|  |  |  |  |  | |
| **Nucleotide Metabolism** | |  |  |  | |
|  |  |  |  |  | |
| A_92_P029107 | 23.1.2.20 | nucleotide metabolism.synthesis.purine.adenyosuccinate synthase | 1365 similar to PDB:1DJ3_A:7546404:1DJ3_A Chain A, Structures Of Adenylosuccinate Synthetase From And Arabidopsis Thaliana. (Triticum aestivum), partial (90%) | 0.82 | |
| A_92_P031851 | 23.4.99 | nucleotide metabolism.phosphotransfer and pyrophosphatases.misc | 1311 UP:P93410_ORYSA (P93410) Vacuolar H+-pyrophosphatase (Ovp2) , partial (40%) | 0.75 | |
|  |  |  |  |  | |
|  |  |  |  |  | |
| **Stress** |  |  |  |  | |
|  |  |  |  |  | |
| A_92_P025901 | 20.1 | stress.biotic | 1668 similar to PRF:NP_195579.2:30692042:NP_195579 pathogenesis-related thaumatin family protein (Arabidopsis thaliana), partial (74%) | 1.88 | |
| A_92_P015451 | 20 | stress | 999 similar to UP:Q5ZBH4_ORYSA (Q5ZBH4) Myc-regulated DEAD:H box 18 RNA helicase-like, partial (35%) | 1.55 | |
| A_92_P001677 | 20.1 | stress.biotic | 1463 similar to PRF:NP_195579.2:30692042:NP_195579 pathogenesis-related thaumatin family protein (Arabidopsis thaliana), partial (74%) | 1.49 | |
| A_92_P010408 | 20.2.99 | stress.abiotic.unspecified | 1351 similar to UP:Q9ZTR5_HORVU (Q9ZTR5) Dehydrin 6, partial (28%) | 1.42 | |
| A_92_P009280 | 20.2.2 | stress.abiotic.cold | similar to GB\|AAK82513.1\|15081717\|AY048251 At2g37220/F3G5.1 {Arabidopsis thaliana} (exp=-1; wgp=0; cg=0), partial (43%), poly(U) binding, RNA binding, nucleic acid binding, response to cold | 1.35 | |
| A_92_P004714 | 20.2.2 | stress.abiotic.cold | 2308 similar to UP:Q69JW8_ORYSA (Q69JW8) Loricrin-like protein, partial (46%) | 1.15 | |
| A_92_P041455 | 20.2.5 | stress.abiotic.light | 1955 similar to PRF:NP_566520.1:18400841:NP_566520 6-4 photolyase (UVR3) (Arabidopsis thaliana), partial (76%), UVB repair | 1.15 | |
| A_92_P019347 | 20 | stress | 700 similar to UP:Q5ZBH4_ORYSA (Q5ZBH4) Myc-regulated DEAD:H box 18 RNA helicase-like, partial (24%) | 1.04 | |
| A_92_P006553 | 20.2.1 | stress.abiotic.heat | 905 similar to UP:Q6Z7B3_ORYSA (Q6Z7B3) Heat shock factor protein hsf8-like, partial (35%) | 0.97 | |
| A_92_P012975 | 20.2.5 | stress.abiotic.light | 734 homologue to UP:Q5N797_ORYSA (Q5N797) UVB-resistance protein UVR8-like, partial (91%) | 0.94 | |
| A_92_P008038 | 20.2.1 | stress.abiotic.heat | 1461 similar to UP:Q6EPX0_ORYSA (Q6EPX0) Chaperone protein dnaJ-related-like, partial (84%) | 0.87 | |
| A_92_P025174 | 20.2.5 | stress.abiotic.light | 836 homologue to UP:Q5N797_ORYSA (Q5N797) UVB-resistance protein UVR8-like, complete | 0.86 | |
| A_92_P028434 | 20.2.1 | stress.abiotic.heat | 1028 homologue to UP:Q43638_SECCE (Q43638) Heat-shock protein precursor, partial (33%) | 0.71 | |
|  |  |  |  |  | |
|  |  |  |  |  | |
| **Hormone Metabolism** | |  |  |  | |
|  |  |  |  |  | |
| A_92_P012734 | 17.8 | hormone metabolism.salicylic acid | 889 weakly similar to UP:ICS_CATRO (Q9ZPC0) Isochorismate synthase, chloroplast precursor , partial (16%), synthesize salicylic acid for defense | 1.35 | |
| A_92_P026121 | 17.5.3 | hormone metabolism.ethylene.induced-regulated-responsive-activated | 1875 similar to UP:Q5MFV3_ORYSA (Q5MFV3) BTH-induced ERF transcriptional factor 1, partial (36%) benzothiadiazole( BTH )- induced ethylene responsive transcriptional factors | 1.14 | |
| A_92_P027654 | 17.1.2 | hormone metabolism.abscisic acid.signal transduction | 587 similar to UP:PRR95_ORYSA (Q689G6) Two-component response regulator-like PRR95 (Pseudo-response regulator 95) (OsPRR95), partial (27%) | 1.06 | |
| A_92_P031740 | 17.1.2 | hormone metabolism.abscisic acid.signal transduction | 818 similar to UP:PRR95_ORYSA (Q689G6) Two-component response regulator-like PRR95 (Pseudo-response regulator 95) (OsPRR95), partial (17%) | 1.02 | |
| A_92_P035267 | 17.1.2 | hormone metabolism.abscisic acid.signal transduction | 1524 similar to UP:PRR95_ORYSA (Q689G6) Two-component response regulator-like PRR95 (Pseudo-response regulator 95) (OsPRR95), partial (52%) | 1.01 | |
| A_92_P025367 | 17.1.2 | hormone metabolism.abscisic acid.signal transduction | 745 similar to PRF:NP_973703.1:42571259:NP_973703 pseudo-response regulator 9 (APRR9) : timing of CAB expression 1-like protein (TL1) (Arabidopsis thaliana), partial (15%) | 0.92 | |
| A_92_P035854 | 17.1.2 | hormone metabolism.abscisic acid.signal transduction | 4249 UP:RPOB_MAIZE (P16023) DNA-directed RNA polymerase beta chain (PEP) (Plastid-encoded RNA polymerase beta subunit) (RNA polymerase beta subunit) , complete | 0.76 | |
|  |  |  |  |  | |
|  |  |  |  |  | |
| **Secondary Metabolism** | |  |  |  | |
|  |  |  |  |  | |
| A_92_P006173 | 16.7 | secondary metabolism.wax | 2405 similar to UP:O04693_ORYSA (O04693) Glossy1 homolog (Fragment), partial (94%) | 1.44 | |
| A_92_P020451 | 16.8.4 | secondary metabolism.flavonoids.flavonols | 1475 similar to UP:Q5JM91_ORYSA (Q5JM91) Ripening-related protein-like, partial (95%) | 1.02 | |
| A_92_P027353 | 16.1.4 | secondary metabolism.isoprenoids.carotenoids | 1623 UP:Q6EI12_MAIZE (Q6EI12) Phytoene synthase 2, complete | 0.99 | |
| A_92_P027411 | 16.7 | secondary metabolism.wax | 2406 similar to UP:O04693_ORYSA (O04693) Glossy1 homolog (Fragment), partial (88%) | 0.95 | |
| A_92_P029992 | 16.7 | secondary metabolism.wax | 2195 UP:Q6RBX9_MAIZE (Q6RBX9) Glossy1 protein, complete | 0.90 | |
| A_92_P004456 | 16.2.1.05 | secondary metabolism.phenylpropanoids.lignin biosynthesis.C3H | 1299 weakly similar to UP:Q9LFB5_ARATH (Q9LFB5) Anthranilate N-benzoyltransferase-like protein (AT5g01210:F7J8_190), partial (39%) | 0.89 | |
| A_92_P018149 | 16.1.4 | secondary metabolism.isoprenoids.carotenoids | 1655 Zea mays phytoene synthase (Y1) gene, complete cds | 0.89 | |
| A_92_P041371 | 16.8.2 | secondary metabolism.flavonoids.chalcones | ACG35950 chalcone isomerase [Zea mays] | 0.87 | |
| A_92_P025926 | 16.8.3 | secondary metabolism.flavonoids.dihydroflavonols | 1474 weakly similar to GB:AAT85328.1:50881483:AC091670 UDP-glucoronosyl and UDP-glucosyl transferase domain containing protein (Oryza sativa (japonica cultivar-group)), partial (24%) | 0.82 | |
| A_92_P028365 | 16.1.1 | secondary metabolism.isoprenoids.non-mevalonate pathway | 1849 homologue to UP:Q8W250_ORYSA (Q8W250) 1-deoxy-D-xylulose 5-phosphate reductoisomerase precursor , partial (94%) | 0.73 | |
| A_92_P038053 | 16.1.4 | secondary metabolism.isoprenoids.carotenoids | 2196 UP:CRTI_MAIZE (P49086) Phytoene dehydrogenase, chloroplast precursor (Phytoene desaturase) , complete | 0.72 | |
|  |  |  |  |  | |
|  |  |  |  |  | |
| **Amino Acid Metabolism** | |  |  |  | |
|  |  |  |  |  | |
| A_92_P017581 | 13.2.3.5 | amino acid metabolism.degradation.aspartate family.lysine | 2871 acetoacetyl CoA thiolase | 1.07 | |
| A_92_P006156 | 13.1.1.3 | amino acid metabolism.synthesis.central amino acid metabolism.alanine | 1235 homologue to UP:O82443_MAIZE (O82443) Alanine aminotransferase , partial (60%) | 0.91 | |
| A_92_P029838 | 13.1.1.3 | amino acid metabolism.synthesis.central amino acid metabolism.alanine | 818 homologue to UP:O82443_MAIZE (O82443) Alanine aminotransferase , partial (39%) | 0.83 | |
| A_92_P011008 | 13.1.6.5 | amino acid metabolism.synthesis.aromatic aa.tryptophan | 777 similar to UP:Q5PP33_ARATH (Q5PP33) At5g19500, partial (20%) | 0.80 | |
|  |  |  |  |  | |
|  |  |  |  |  | |
| **N-Metabolism** | |  |  |  | |
|  |  |  |  |  | |
| A_92_P025617 | 12.2.01 | N-metabolism.ammonia metabolism.glutamate synthase | 2311 homologue to UP:Q9ZNX7_ORYSA (Q9ZNX7) NADH dependent Glutamate Synthase precursor , partial (31%) | 0.76 | |
|  |  |  |  |  | |
|  |  |  |  |  | |
| **Lipid Metabolism** | |  |  |  | |
|  |  |  |  |  | |
| A_92_P015609 | 11.9.2 | lipid metabolism.lipid degradation.lipases | 1918 homologue to UP:Q6ZLH2_ORYSA (Q6ZLH2) Lipase-like protein, partial (89%) | 1.11 | |
| A_92_P012132 | 11.3 | lipid metabolism.Phospholipid synthesis | ABF83429 S-adenosyl-L-methionine: phosphoethanolamine N-methyltransferase [Zea mays] | 1.07 | |
| A_92_P007417 | 11.2.03 | lipid metabolism.FA desaturation.omega 3 desaturase | ACG28208 omega-3 fatty acid desaturase [Zea mays] | 1.04 | |
| A_92_P018620 | 11.9.3 | lipid metabolism.lipid degradation.lysophospholipases | 955 similar to UP:Q6DW08_MEDSA (Q6DW08) GMPase, partial (48%) | 0.99 | |
| A_92_P021497 | 11.1.06 | lipid metabolism.FA synthesis and FA elongation.enoyl ACP reductase | ACG42145 enoyl-[acyl-carrier-protein] reductase [NADH] [Zea mays] | 0.97 | |
| A_92_P019173 | 11.3 | lipid metabolism.Phospholipid synthesis | 2320 similar to UP:Q8VYX1_WHEAT (Q8VYX1) Phosphoethanolamine methyltransferase, partial (98%) | 0.97 | |
| A_92_P022841 | 11.1.08 | lipid metabolism.FA synthesis and FA elongation.acyl coa ligase | AAX93005 probable acyl-CoA synthetase, 62297-59022 [imported] - Arabidopsis thaliana [Oryza sativa (japonica cultivar-group)] | 0.74 | |
|  |  |  |  |  | |
|  |  |  |  |  | |
| **Cell Wall** |  |  |  |  | |
|  |  |  |  |  | |
| A_92_P006369 | 10.5.2 | cell wall.cell wall proteins.proline rich proteins | 380 weakly similar to UP:Q41122_PHAVU (Q41122) Proline-rich protein precursor, partial (8%) | 1.50 | |
| A_92_P033798 | 10.5.3 | cell wall.cell wall proteins.LRR | 748 weakly similar to UP:Q5GMM0_CAPCH (Q5GMM0) Extensin-like protein, partial (74%) | 1.32 | |
| A_92_P011124 | 10.6.3 | cell wall.degradation.pectate lyases and polygalacturonases | ACG36195 2,3-bisphosphoglycerate-independent phosphoglycerate mutase [Zea mays] | 0.95 | |
|  |  |  |  |  | |
|  |  |  |  |  | |
| **Chloroplast** | |  |  |  | |
|  |  |  |  |  | |
| A_92_P024404 | 1.05.03 | PS.chloroplast.biogenesis | 776 similar to UP:Q8RX79_ARATH (Q8RX79) AT3g62910:T20O10_10 (Chloroplast Release Factor 1), partial (32%) | 1.17 | |
| A_92_P003033 | 1.05.03 | PS.chloroplast.biogenesis | 950 similar to PRF:NP_564544.1:18402618:NP_564544 cytochrome c biogenesis protein family (Arabidopsis thaliana), partial (37%) | 0.97 | |
| A_92_P014384 | 1.05.02 | PS.chloroplast.protein | 2211 PIR:S58532:S58532 matK protein (trnK intron) - maize chloroplast (Zea mays), complete | 0.86 | |
| A_92_P026796 | 1.05.02 | PS.chloroplast.protein | 2211 PIR:S58532:S58532 matK protein (trnK intron) - maize chloroplast (Zea mays), complete | 0.82 | |
| A_92_P021461 | 1.05.02 | PS.chloroplast.protein | 2211 PIR:S58532:S58532 matK protein (trnK intron) - maize chloroplast (Zea mays), complete | 0.81 | |
| A_92_P038661 | 1.05.02 | PS.chloroplast.protein | 2211 PIR:S58532:S58532 matK protein (trnK intron) - maize chloroplast (Zea mays), complete | 0.81 | |
| A_92_P014558 | 1.05.01 | PS.chloroplast.transcription | BAD81964 Chloroplast ORF70 [Oryza sativa Japonica Group] | 0.79 | |
| A_92_P009998 | 1.05.02 | PS.chloroplast.protein | 2211 PIR:S58532:S58532 matK protein (trnK intron) - maize chloroplast (Zea mays), complete | 0.77 | |
| A_92_P016685 | 1.05.02 | PS.chloroplast.protein | 1205 similar to UP:HIS2_ARATH (O82768) Histidine biosynthesis bifunctional protein hisIE, chloroplast precursor [Includes: Phosphoribosyl-AMP cyclohydrolase (PRA-CH) | 0.72 | |
| A_92_P034506 | 1.05.02 | PS.chloroplast.protein | 2297 weakly similar to UP:O24293_PEA (O24293) Chloroplast inner envelope protein, 110 kD (IEP110) precursor, partial (32%) | 0.72 | |
|  |  |  |  |  | |
|  |  |  |  |  | |
| **Photosystem Light Reactions** | | |  |  | |
|  |  |  |  |  | |
| A_92_P001685 | 1.01.05.02 | PS.lightreaction.other electron carrier (ox/red).ferredoxin | ACG40210 3Fe-4S ferredoxin [Zea mays] | 2.73 | |
| A_92_P000003 | 1.01.01.02.05 | PS.lightreaction.photosystem II.PSII polypeptide subunits.NADH | 901 UP:NU1C_MAIZE (P25706) NAD(P)H-quinone oxidoreductase chain 1, chloroplast (NAD(P)H dehydrogenase, chain 1) (NADH-plastoquinone oxidoreductase chain 1) , partial (24%) | 1.91 | |
| A_92_P017012 | 1.01.01.01.02 | PS.lightreaction.photosystem II.LHC-II.RC | 780 homologue to UP:Q8S3I9_WHEAT (Q8S3I9) D1 protease precursor (Fragment), partial (44%) | 1.75 | |
| A_92_P033677 | 1.01.01.02.06 | PS.lightreaction.photosystem II.PSII polypeptide subunits.thylakoid | 924 similar to PRF:NP_200161.2:30696347:NP_200161 thylakoid lumenal 17.4 kDa protein, chloroplast (Arabidopsis thaliana), partial (69%) | 1.41 | |
| A_92_P028453 | 1.01.03 | PS.lightreaction.cytochrome b6/f | 699 UP:Q6L372_9POAL (Q6L372) Cytochrome b6 , complete | 1.22 | |
| A_92_P020911 | 1.01.05.03 | PS.lightreaction.other electron carrier (ox/red).ferredoxin reductase | 1719 homologue to UP:FENR2_ORYSA (P41345) Ferredoxin--NADP reductase, root isozyme, chloroplast precursor (FNR) , partial (98%) | 1.21 | |
| A_92_P023933 | 1.01.05.03 | PS.lightreaction.other electron carrier (ox/red).ferredoxin reductase | 1203 homologue to PIR:JA0172:JA0172 ferredoxin-nitrite reductase precursor - maize (fragment) (Zea mays) , partial (24%) | 1.15 | |
| A_92_P031954 | 1.01.01.02.05 | PS.lightreaction.photosystem II.PSII polypeptide subunits.NADH | 2891 homologue to UP:NU5C_MAIZE (P46620) NAD(P)H-quinone oxidoreductase chain 5, chloroplast (NAD(P)H dehydrogenase, chain 5) (NADH-plastoquinone oxidoreductase chain 5) , complete | 1.08 | |
| A_92_P037087 | 1.01.01.02.05 | PS.lightreaction.photosystem II.PSII polypeptide subunits.NADH | 735 UP:NU4LC_MAIZE (P11646) NAD(P)H-quinone oxidoreductase chain 4L, chloroplast (NAD(P)H dehydrogenase, chain 4L) (NADH-plastoquinone oxidoreductase chain 4L) , complete | 1.05 | |
| A_92_P006945 | 1.01.04 | PS.lightreaction.ATP synthase | 1600 UP:ATPA_MAIZE (P05022) ATP synthase alpha chain , complete | 1.01 | |
| A_92_P005796 | 1.01.01.02.04 | PS.lightreaction.photosystem II.PSII polypeptide subunits.O | ACG28532 oxygen evolving enhancer protein 3 [Zea mays] | 0.99 | |
| A_92_P040336 | 1.01.01.02.03 | PS.lightreaction.photosystem II.PSII polypeptide subunits.RC | 3157 UP:PSBC_MAIZE (P48187) Photosystem II 44 kDa reaction center protein (P6 protein) (CP43), complete | 0.95 | |
| A_92_P035201 | 1.01.01.01.07 | PS.lightreaction.photosystem II.LHC-II.Light | 1649 UP:PSBB_MAIZE (P05641) Photosystem II P680 chlorophyll A apoprotein (CP-47 protein), complete | 0.93 | |
| A_92_P032975 | 1.01.01.02.05 | PS.lightreaction.photosystem II.PSII polypeptide subunits.NADH | NP_180560 NDA2 (ALTERNATIVE NAD(P)H DEHYDROGENASE 2); NADH dehydrogenase [Arabidopsis thaliana] | 0.91 | |
| A_92_P009104 | 1.01.01.02.05 | PS.lightreaction.photosystem II.PSII polypeptide subunits.NADH | YP_899454 NADH-plastoquinone oxidoreductase subunit 5 [Sorghum bicolor] | 0.86 | |
| A_92_P041578 | 1.01.01.01.07 | PS.lightreaction.photosystem II.LHC-II.Light | 850 similar to UP:Q945R7_ORYSA (Q945R7) Violaxanthin de-epoxidase precursor, partial (43%), nonphotochemical quenching in excessive light | 0.86 | |
| A_92_P019403 | 1.01.01.02.06 | PS.lightreaction.photosystem II.PSII polypeptide subunits.thylakoid | 843 similar to UP:TL15_ARATH (O22160) Thylakoid lumenal 15 kDa protein, chloroplast precursor (p15), partial (70%) | 0.84 | |
| A_92_P010453 | 1.01.01.01.07 | PS.lightreaction.photosystem II.LHC-II.Light | NP_001105374 chlorophyll a/b-binding apoprotein CP26 precursor [Zea mays] | 0.81 | |
| A_92_P010649 | 1.01.01.02.06 | PS.lightreaction.photosystem II.PSII polypeptide subunits.thylakoid | 802 similar to UP:TL215_ARATH (O23403) Thylakoid lumenal 21.5 kDa protein, chloroplast precursor, partial (62%) | 0.80 | |
| A_92_P040737 | 1.01.01.02.05 | PS.lightreaction.photosystem II.PSII polypeptide subunits.NADH | 2740 UP:NU1C_MAIZE (P25706) NAD(P)H-quinone oxidoreductase chain 1, chloroplast (NAD(P)H dehydrogenase, chain 1) (NADH-plastoquinone oxidoreductase chain 1) , complete | 0.80 | |
| A_92_P034929 | 1.01.01.02.05 | PS.lightreaction.photosystem II.PSII polypeptide subunits.NADH | YP_874787 NADH-plastoquinone oxidoreductase subunit 4 [Agrostis stolonifera] | 0.77 | |
| A_92_P004626 | 1.01.01.02.03 | PS.lightreaction.photosystem II.PSII polypeptide subunits.RC | 939 UP:PSBJ_NYMAL (Q6EW39) Photosystem II reaction center J protein, complete | 0.74 | |
|  |  |  |  |  | |
|  |  |  |  |  | |
| **Development** | |  |  |  | |
|  |  |  |  |  | |
| A_92_P028746 | 33.99 | development.unspecified | 1637 weakly similar to UP:Q7X9Q6_ORYSA (Q7X9Q6) Embryo-specific protein, partial (21%) | 2.42 | |
| A_92_P034047 | 33.99 | development.unspecified | 1103 UP:LEA3_MAIZE (Q42376) Late embryogenesis abundant protein, group 3 (LEA), complete, some LEA genes respond to abiotic stress | 1.65 | |
| A_92_P002996 | 33.99 | root nodule development, development.unspecified | 1372 weakly similar to UP:Q8W0K2_ORYSA (Q8W0K2) MtN3-like, partial (70%), nodulin | 1.48 | |
| A_92_P035967 | 33.99 | development.unspecified | 1750 UP:Q58I00_MAIZE (Q58I00) 1-deoxy-D-xylulose 5-phosphate synthase 1 (Fragment), partial (91%), overexpressing lines showed an increased accumulation of MEP- derived plastid isoprenoids such as chlorophylls, carotenoids, and taxadiene, light response, circadian clock, plastid targeted in chloroplast | 1.30 | |
| A_92_P007621 | 33.99 | development.unspecified | 813 homologue to UP:LEA3_MAIZE (Q42376) Late embryogenesis abundant protein, group 3 (LEA), partial (90%) | 1.26 | |
|  |  |  |  |  | |
|  |  |  |  |  | |
| **Cell Functions** | |  |  |  | |
|  |  |  |  |  | |
| A_92_P015815 | 31.1 | cell.organisation | 670 similar to UP:Q9SSZ6_ORYSA (Q9SSZ6) Cyclin, partial (18%) | 1.03 | |
| A_92_P035079 | 31.1 | cell.organisation | 751 weakly similar to PRF:NP_191914.1:15236688:NP_191914 plastid-lipid associated protein PAP : fibrillin family protein (Arabidopsis thaliana), partial (58%), fibrillin accumulation by ab acid helps enhance tolerance of light stess-triggerd photoinhibition in PSII | 1.02 | |
| A_92_P023148 | 31.1 | cell.organisation | ABG21984 Cyclin, N-terminal domain containing protein, expressed [Oryza sativa (japonica cultivar-group)] | 0.89 | |
| A_92_P031726 | 31.1 | cell.organisation | 738 similar to UP:Q5Z9S9_ORYSA (Q5Z9S9) Kinesin 4-like, partial (13%) | 0.88 | |
| A_92_P022461 | 31.1 | cell.cycle | 1096 weakly similar to PRF:NP_191871.1:15229382:NP_191871 cyclin family protein (Arabidopsis thaliana), partial (48%) | 0.86 | |
| A_92_P019092 | 31.2 | cell.division | 1243 similar to UP:RRFC_SPIOL (P82231) Ribosome recycling factor, chloroplast precursor (Ribosome-releasing factor, chloroplast) (RRF) (CpFrr) (RRFHCP), partial (71%) | 0.79 | |
|  |  |  |  |  | |
|  |  |  |  |  | |
| **Redox** |  |  |  |  | |
|  |  |  |  |  | |
| A_92_P016279 | 21.01 | redox.thioredoxin | 1195 similar to GB:AAD35009.1:4973264:AF144391 thioredoxin-like 5 (Arabidopsis thaliana), partial (46%) | 1.57 | |
| A_92_P014797 | 21.01 | redox.thioredoxin | 1417 similar to UP:Q40230_LILLO (Q40230) Thioredoxin, partial (60%) | 1.19 | |
|  |  |  |  |  | |
|  |  |  |  |  | |
| **Tetrapyrrole Biosynthesis** | | |  |  | |
|  |  |  |  |  | |
| A_92_P041889 | 19.14 | tetrapyrrole synthesis.protochlorophyllide reductase | 1308 homologue to UP:PORA_WHEAT (Q41578) Protochlorophyllide reductase A, chloroplast precursor (PCR A) (NADPH-protochlorophyllide oxidoreductase A) (POR A) , partial (78%) | 1.34 | |
| A_92_P036568 | 19.2 | tetrapyrrole synthesis.glu-tRNA reductase | 954 homologue to UP:HEM1_ORYSA (O48674) Glutamyl-tRNA reductase, chloroplast precursor (GluTR) , partial (28%) | 0.99 | |
| A_92_P006618 | 19.2 | tetrapyrrole synthesis.glu-tRNA reductase | 2136 homologue to UP:HEM1_ORYSA (O48674) Glutamyl-tRNA reductase, chloroplast precursor (GluTR) , partial (94%) | 0.98 | |
| A_92_P009811 | 19.2 | tetrapyrrole synthesis.glu-tRNA reductase | 789 homologue to UP:HEM1_ORYSA (O48674) Glutamyl-tRNA reductase, chloroplast precursor (GluTR) , partial (30%) | 0.85 | |
| A_92_P024338 | 19.10 | tetrapyrrole synthesis.magnesium chelatase | CAA04526 magnesium chelatase subunit [Glycine max] | 0.78 | |
|  |  |  |  |  | |
|  |  |  |  |  | |
| **C1 Metabolism** | |  |  |  | |
|  |  |  |  |  | |
| A_92_P034671 | 25 | C1-metabolism | 1016 similar to UP:Q6TFM2_LYCES (Q6TFM2) Dihydroneopterin aldolase, partial (91%) | 1.33 | |
|  |  |  |  |  | |
|  |  |  |  |  | |
| **Misc.** |  |  |  |  | |
|  |  |  |  |  | |
| A_92_P031745 | 26.22 | misc.short chain dehydrogenase/reductase (SDR) | 1260 similar to UP:Q93ZA0_ARATH (Q93ZA0) AT4g13250:F17N18_140, partial (51%) | 1.71 | |
| A_92_P041107 | 26.04 | misc.beta 1,3 glucan hydrolases | 1016 weakly similar to UP:Q45X99_9ROSA (Q45X99) Beta-1,3-glucanase 2, partial (18%) | 1.37 | |
| A_92_P022116 | 26.09 | misc.glutathione S transferases | 1133 UP:Q9FQB0_MAIZE (Q9FQB0) Glutathione S-transferase GST 29 , complete | 1.19 | |
| A_92_P006949 | 26.22 | misc.short chain dehydrogenase/reductase (SDR) | 875 weakly similar to UP:Q93ZA0_ARATH (Q93ZA0) AT4g13250:F17N18_140, partial (29%) | 1.18 | |
| A_92_P031846 | 26.03 | misc.gluco-, galacto- and mannosidases | 849 similar to PRF:NP_193907.2:22328863:NP_193907 glycosyl hydrolase family 1 protein (Arabidopsis thaliana), partial (13%) | 1.04 | |
| A_92_P030655 | 26.10 | misc.cytochrome P450 | NP_190881 LUT1 (LUTEIN DEFICIENT 1); oxygen binding [Arabidopsis thaliana] | 0.96 | |
| A_92_P002479 | 26.11 | misc.alcohol dehydrogenases | 1424 similar to UP:Q8S411_LOLPR (Q8S411) Cinnamyl alcohol dehydrogenase, partial (92%) | 0.89 | |
| A_92_P024905 | 26.02 | misc.UDP glucosyl and glucoronyl transferases | 756 weakly similar to UP:Q9SBL1_SORBI (Q9SBL1) UDP-glucose glucosyltransferase, partial (25%) | 0.85 | |
| A_92_P023173 | 26.03 | misc.gluco-, galacto- and mannosidases | 1320 weakly similar to GB:AAF70821.1:7939617:AF154420 beta-galactosidase (Lycopersicon esculentum), partial (25%) | 0.85 | |
| A_92_P011551 | 26.03 | misc.gluco-, galacto- and mannosidases | 1320 weakly similar to GB:AAF70821.1:7939617:AF154420 beta-galactosidase (Lycopersicon esculentum), partial (25%) | 0.84 | |
| A_92_P020053 | 26.23 | misc.rhodanese | 706 similar to UP:Q5N838_ORYSA (Q5N838) Rhodanese domain-containing protein-like, partial (23%) | 0.78 | |
| A_92_P005836 | 26.23 | misc.rhodanese | 589 similar to UP:Q6ZI49_ORYSA (Q6ZI49) Rhodanese-like domain-containing protein-like, partial (57%) | 0.76 | |
| A_92_P028128 | 26.10 | misc.cytochrome P450 | 583 UP:Q8VYA8_MAIZE (Q8VYA8) Cytochrome P450 monooxygenase CYP71C3v2, partial (6%) | 0.75 | |
| A_92_P023099 | 27.06 | misc.oxidases - copper, flavone etc. | 1306 similar to PIR:T05166:T05166 quinone reductase homolog F18E5.200 (Arabidopsis thaliana), partial (89%) | 0.74 | |
|  |  |  |  |  | |
|  |  |  |  |  | |
| **Minor and Major CHO Metabolism** | | |  |  | |
|  |  |  |  |  | |
| A_92_P039958 | 3.4.03 | minor CHO metabolism.myo-inositol.InsP Synthases | 2171 homologue to GB:AAG40328.1:11762100:AF323175 myo-inositol 1-phosphate synthase (Zea mays), complete, under control of circadian clock | 1.50 | |
| A_92_P030731 | 3.7 | minor CHO metabolism.sugar kinases | 776 similar to UP:Q4M0P2_9BURK (Q4M0P2) Xylulokinase, partial (3%) | 1.43 | |
| A_92_P030330 | 3.5 | minor CHO metabolism.others | 779 similar to PRF:NP_564718.2:30696124:NP_564718 haloacid dehalogenase-like hydrolase family protein (Arabidopsis thaliana), partial (16%) | 1.21 | |
| A_92_P041403 | 2.2.2.04 | major CHO metabolism.degradation.starch.D enzyme | 1367 similar to UP:Q9LV91_ARATH (Q9LV91) 4-alpha-glucanotransferase (AT5g64860:MXK3_9), partial (62%) | 1.20 | |
| A_92_P003267 | 2.2.2.01 | major CHO metabolism.degradation.starch.starch cleavage | 1503 similar to UP:Q5BLY3_MALDO (Q5BLY3) Plastid alpha-amylase, partial (35%) | 0.90 | |
| A_92_P022813 | 3.8.2 | minor CHO metabolism.galactose.alpha-galactosidases | 1058 similar to PRF:NP_189269.2:30688284:NP_189269 glycosyl hydrolase family protein 27 : alpha-galactosidase family protein : melibiase family protein (Arabidopsis thaliana), partial (3%) | 0.88 | |
|  |  |  |  |  | |
|  |  |  |  |  | |
| **Metal Handling** | |  |  |  | |
|  |  |  |  |  | |
| A_92_P037563 | 15 | metal handling | ACG29581 selenium-binding protein-like [Zea mays] | 0.71 | |
|  |  |  |  |  | |
|  |  |  |  |  | |
| **Vitamin Metabolism** | |  |  |  | |
|  |  |  |  |  | |
| A_92_P004063 | 18 | Co-factor and vitamine metabolism | 751 similar to UP:Q93Z45_ARATH (Q93Z45) At1g02880:F22D16_33, partial (83%) | 1.01 | |
| A_92_P023416 | 18 | Co-factor and vitamine metabolism | 575 similar to UP:THIC_BACSU (P45740) Thiamine biosynthesis protein thiC, partial (13%) | 0.92 | |
|  |  |  |  |  | |
|  |  |  |  |  | |
| **Fermentation** | |  |  |  | |
|  |  |  |  |  | |
| A_92_P001553 | 5.10 | fermentation.aldehyde dehydrogenase | 1928 UP:Q8S532_MAIZE (Q8S532) Cytosolic aldehyde dehydrogenase RF2C, complete | 0.87 | |
|  |  |  |  |  | |
|  |  |  |  |  | |
| **Glycolysis** |  |  |  |  | |
|  |  |  |  |  | |
| A_92_P003661 | 4.11 | glycolysis.phosphoglycerate mutase | 572 GB:CAA83914.1:551288:ZMPHMU1 phosphoglycerate mutase (Zea mays), partial (29%) | 0.93 | |
| A_92_P037596 | 4.13 | glycolysis.PK | 1357 similar to GB:AAL47446.1:17978970:AY069894 At2g36580:F1O11.21 (Arabidopsis thaliana), partial (75%) | 0.80 | |
|  |  |  |  |  | |
|  |  |  |  |  | |
| **No Ontology** | |  |  |  | |
|  |  |  |  |  | |
| A_92_P024018 | 35.2 | not assigned.unknown | 975 GB:BAB47035.1:13928206:AB042240 ycf3 (Triticum aestivum), partial (31%) | 1.23 | |
| A_92_P003122 | 35.1 | not assigned.no ontology | NP_001105791 shugosin centromeric cohesion1 [Zea mays], meiosis 1 centromere fusion | 1.04 | |
| A_92_P021563 | 35.1.5 | not assigned.no ontology.pentatricopeptide (PPR) repeat-containing protein | 1356 similar to UP:Q653J1_ORYSA (Q653J1) PPR-protein-like, partial (61%) | 1.01 | |
| A_92_P012604 | 35.2 | not assigned.unknown | 1396 similar to UP:Q8H1U5_ARATH (Q8H1U5) APC2, partial (37%), anaphase-promoting complex/cyclosome | 1.00 | |
| A_92_P007141 | 35.1.40 | not assigned.no ontology.glycine rich proteins | similar to UP\|Q2IIH7 ANADE (Q2IIH7) PE-PGRS family protein, partial (6%) | 1.00 | |
| A_92_P007943 | 35.1.5 | not assigned.no ontology.pentatricopeptide (PPR) repeat-containing protein | BAD07548 pentatricopeptide (PPR) repeat-containing protein-like [Oryza sativa Japonica Group] | 0.99 | |
| A_92_P024941 | 35.1.19 | not assigned.no ontology.C2 domain-containing protein | 2024 similar to UP:Q9LXU2_ARATH (Q9LXU2) Anthranilate phosphoribosyltransferase-like protein, partial (64%) | 0.96 | |
| A_92_P018114 | 35.2 | not assigned.unknown | 731 similar to UP:Q69Y52_ORYSA (Q69Y52) BHLH transcription factor PTF1, partial (19%) | 0.95 | |
| A_92_P000679 | 35.1 | not assigned.no ontology | 1785 similar to UP:Q76FS5_ARATH (Q76FS5) Solanesyl diphosphate synthase 2 , partial (76%) | 0.92 | |
| A_92_P026743 | 35.1 | not assigned.no ontology | 801 similar to UP:Q5NBD6_ORYSA (Q5NBD6) 5 -nucleotidase, cytosolic II-like, partial (13%) | 0.92 | |
| A_92_P001545 | 35.1 | not assigned.no ontology | AAB61961 integral membrane protein [Oryza sativa] | 0.91 | |
| A_92_P010563 | 35.1 | not assigned.no ontology | 1555 similar to UP:Q67UY4_ORYSA (Q67UY4) Rhomboid-like protein, partial (96%) | 0.91 | |
| A_92_P019088 | 35.1.5 | not assigned.no ontology.pentatricopeptide (PPR) repeat-containing protein | 591 weakly similar to UP:Q6YWP8_ORYSA (Q6YWP8) Pentatricopeptide (PPR) repeat-containing protein-like, partial (10%) | 0.89 | |
| A_92_P025857 | 35.1 | not assigned.no ontology | 1189 similar to PRF:NP_178473.2:30678076:NP_178473 translin family protein (Arabidopsis thaliana), partial (77%) | 0.85 | |
| A_92_P026360 | 35.2 | not assigned.unknown | EEF29779 DNA polymerase epsilon subunit, putative [Ricinus communis] | 0.85 | |
| A_92_P034419 | 35.1.19 | not assigned.no ontology.C2 domain-containing protein | 1084 similar to UP:ERG3_ORYSA (Q7F9X0) Elicitor-responsive protein 3 (16 kDa phloem protein) (RPP16), complete | 0.80 | |
| A_92_P033639 | 35.1.5 | not assigned.no ontology.pentatricopeptide (PPR) repeat-containing protein | BAD15896 PPR-repeat protein-like [Oryza sativa Japonica Group] | 0.78 | |
| A_92_P025703 | 35.1 | not assigned.no ontology | 1024 similar to GB:AAN31783.1:23396189:AC134516 Putataive pollen specific protein C13 precursor (Oryza sativa (japonica cultivar-group)), partial (88%) | 0.73 | |
| A_92_P033974 | 35.1 | not assigned.no ontology | 1392 similar to UP:Q6Z539_ORYSA (Q6Z539) CAAX amino terminal protease family-like protein, partial (64%) | 0.73 | |
| A_92_P034079 | 35.1.5 | not assigned.no ontology.pentatricopeptide (PPR) repeat-containing protein | 534 weakly similar to PRF:NP_177601.1:15221306:NP_177601 pentatricopeptide (PPR) repeat-containing protein (Arabidopsis thaliana), partial (15%) | 0.70 | |
|  |  |  |  |  | |
|  |  |  |  |  | |
| **Unknown** |  |  |  |  | |
|  |  |  |  |  | |
| A_92_P032231 | N/A | N/A | 1152 similar to UP:Q9SII5_ARATH (Q9SII5) Expressed protein (At2g17230:T23A1.9), partial (69%) | 2.73 | |
| A_92_P023273 | N/A | N/A | weakly similar to XP_804629 dispersed gene family protein 1 (DGF-1) [Trypanosoma cruzi strain CL Brener] | 2.66 | |
| A_92_P031228 | N/A | N/A | 3474 Zea mays clone EL01N0515H05.c mRNA sequence | 2.33 | |
| A_92_P028320 | N/A | N/A | 735 unknown | 2.27 | |
| A_92_P024009 | N/A | N/A | 2624 Zea mays clone EL01T0203B04.c mRNA sequence | 2.04 | |
| A_92_P035573 | N/A | N/A | 1492 similar to PRF:NP_568615.2:30694168:NP_568615 expressed protein (Arabidopsis thaliana), partial (9%) | 1.99 | |
| A_92_P024585 | N/A | N/A | similar to YP_814253 adhesion exoprotein [Lactobacillus gasseri ATCC 33323] | 1.89 | |
| A_92_P019900 | N/A | N/A | 1226 unknown | 1.86 | |
| A_92_P032862 | N/A | N/A | 878 Zea mays clone EK07D2309B08.c mRNA sequence | 1.85 | |
| A_92_P015527 | N/A | N/A | 798 similar to UP:Q70ZY3_MAIZE (Q70ZY3) M22 protein (Fragment), partial (70%) | 1.58 | |
| A_92_P015941 | N/A | N/A | BAD95277 chaperonin precursor [Arabidopsis thaliana] | 1.55 | |
| A_92_P008221 | N/A | N/A | 1986 Zea mays clone EL01N0425D06.d mRNA sequence | 1.53 | |
| A_92_P024279 | N/A | N/A | 988 Zea mays clone EL01N0556C01.c mRNA sequence | 1.45 | |
| A_92_P027560 | N/A | N/A | 1767 weakly similar to UP:Q9FMX6_ARATH (Q9FMX6) Gb:AAD21732.1, partial (34%) | 1.44 | |
| A_92_P028916 | N/A | N/A | XP_001759186 predicted protein [Physcomitrella patens subsp. patens] | 1.42 | |
| A_92_P008382 | N/A | N/A | 1176 similar to UP:O82118_ORYSA (O82118) Zinc finger protein, partial (40%) | 1.42 | |
| A_92_P017780 | N/A | N/A | 892 weakly similar to PRF:NP_201078.1:15241937:NP_201078 integral membrane HPP family protein (Arabidopsis thaliana), partial (63%) | 1.42 | |
| A_92_P001476 | N/A | N/A | NP_001094343 chromosome 16 open reading frame 46 isoform 1 [Homo sapiens] | 1.40 | |
| A_92_P013508 | N/A | N/A | 814 homologue to GB:AAO37991.2:49457926:AC090871 expressed protein (Oryza sativa (japonica cultivar-group)), partial (44%) | 1.33 | |
| A_92_P011932 | N/A | N/A | 1062 similar to PRF:NP_192603.2:42566341:NP_192603 expressed protein (Arabidopsis thaliana), partial (13%) | 1.33 | |
| A_92_P025693 | N/A | N/A | EEF28716 Inner membrane transport protein yjjL, putative [Ricinus communis] | 1.29 | |
| A_92_P013666 | N/A | N/A | 477 unknown | 1.26 | |
| A_92_P037738 | N/A | N/A | AAQ13901 multifunctional protein [Oryza sativa] | 1.24 | |
| A_92_P016645 | N/A | N/A | EEF33979 eukaryotic translation initiation factor 3 subunit, putative [Ricinus communis] | 1.23 | |
| A_92_P012099 | N/A | N/A | 2882 unknown | 1.18 | |
| A_92_P002144 | N/A | N/A | 646 unknown | 1.17 | |
| A_92_P010264 | N/A | N/A | 1037 similar to PRF:NP_565853.1:18404264:NP_565853 expressed protein (Arabidopsis thaliana), partial (66%) | 1.14 | |
| A_92_P012602 | N/A | N/A | 2111 Zea mays clone EL01N0523D10.d mRNA sequence | 1.13 | |
| A_92_P041335 | N/A | N/A | 1027 similar to UP:O82118_ORYSA (O82118) Zinc finger protein, partial (47%) | 1.10 | |
| A_92_P007416 | N/A | N/A | 526 weakly similar to PRF:NP_198091.1:15240916:NP_198091 expressed protein (Arabidopsis thaliana), partial (6%) | 1.09 | |
| A_92_P014307 | N/A | N/A | 425 unknown | 1.09 | |
| A_92_P025992 | N/A | N/A | EEF52409 Paramyosin, putative [Ricinus communis] | 1.08 | |
| A_92_P040332 | N/A | N/A | 752 similar to UP:Q852K0_ORYSA (Q852K0) Expressed protein, partial (30%) | 1.08 | |
| A_92_P038409 | N/A | N/A | 1031 GB:AAB30283.2:13449983:S69194S3 IRF170 (Zea mays), partial (54%) | 1.06 | |
| A_92_P039587 | N/A | N/A | BAC07074 putative oxidoreductase, FAD-binding [Oryza sativa Japonica Group] | 1.06 | |
| A_92_P030818 | N/A | N/A | 632 weakly similar to UP:Q53NQ9_ORYSA (Q53NQ9) F12P19.3 [imported]-Arabidopsis thaliana, partial (22%) | 1.06 | |
| A_92_P005090 | N/A | N/A | EEF35862 pentatricopeptide repeat-containing protein, putative [Ricinus communis] | 1.06 | |
| A_92_P026908 | N/A | N/A | 1099 similar to PRF:NP_176001.2:42562778:NP_176001 GTP-binding protein-related (Arabidopsis thaliana), partial (66%) | 1.04 | |
| A_92_P031081 | N/A | N/A | 1489 weakly similar to PRF:NP_198738.2:30693366:NP_198738 hydrolase, alpha:beta fold family protein (Arabidopsis thaliana), partial (62%), auxin/cell elongation | 1.03 | |
| A_92_P028274 | N/A | N/A | 636 homologue to UP:Q6EPQ3_ORYSA (Q6EPQ3) SPX (SYG1:Pho81:XPR1) domain-containing protein-like, partial (20%) | 1.03 | |
| A_92_P035549 | N/A | N/A | 1084 similar to PRF:NP_568615.2:30694168:NP_568615 expressed protein (Arabidopsis thaliana), partial (9%) | 1.03 | |
| A_92_P024659 | N/A | N/A | similar to UP\|Q2HSJ8 MEDTR (Q2HSJ8) Protein kinase, partial (10%) | 1.02 | |
| A_92_P037728 | N/A | N/A | 1272 weakly similar to PRF:NP_680215.1:22327025:NP_680215 expressed protein (Arabidopsis thaliana), partial (48%) | 1.01 | |
| A_92_P009768 | N/A | N/A | 1414 weakly similar to PRF:NP_567745.1:18416739:NP_567745 antitermination NusB domain-containing protein (Arabidopsis thaliana), partial (60%) | 1.01 | |
| A_92_P001870 | N/A | N/A | 2301 similar to UP:O82261_ARATH (O82261) DegP2 protease (At2g47940:F17A22.33), partial (76%) | 1.01 | |
| A_92_P020367 | N/A | N/A | 664 similar to UP:Q67XR9_ARATH (Q67XR9) MRNA, , clone: RAFL25-28-O12 (At1g29120), partial (14%) | 1.00 | |
| A_92_P005622 | N/A | N/A | 1576 Zea mays clone EL01N0403D03.c mRNA sequence | 0.99 | |
| A_92_P025356 | N/A | N/A | 1532 similar to UP:Q5EAH9_ARATH (Q5EAH9) At3g55760, partial (47%) | 0.99 | |
| A_92_P018109 | N/A | N/A | 623 similar to PRF:NP_172579.1:15220289:NP_172579 expressed protein (Arabidopsis thaliana), partial (5%) | 0.99 | |
| A_92_P033837 | N/A | N/A | 744 weakly similar to PRF:NP_200090.1:15237263:NP_200090 expressed protein (Arabidopsis thaliana), partial (29%) | 0.98 | |
| A_92_P028553 | N/A | N/A | 576 unknown | 0.98 | |
| A_92_P010539 | N/A | N/A | 911 weakly similar to PRF:NP_565570.1:18400547:NP_565570 expressed protein (Arabidopsis thaliana), partial (44%) | 0.95 | |
| A_92_P020519 | N/A | N/A | ACG39255 KHG/KDPG aldolase [Zea mays] | 0.95 | |
| A_92_P023535 | N/A | N/A | 746 similar to UP:Q9LDF8_ARATH (Q9LDF8) Gb:AAF35944.1 (At3g12950), partial (6%) | 0.95 | |
| A_92_P033344 | N/A | N/A | YP_001201351 Type I restriction-modification system methyltransferase subunit [Streptococcus suis 98HAH33] | 0.94 | |
| A_92_P031381 | N/A | N/A | 944 weakly similar to UP:Q4U4H1_9ROSI (Q4U4H1) KH domain-containing protein (Fragment), partial (77%) | 0.93 | |
| A_92_P006610 | N/A | N/A | 3821 Zea mays clone EL01N0551H02.c mRNA sequence | 0.93 | |
| A_92_P002333 | N/A | N/A | 204 unknown | 0.93 | |
| A_92_P017030 | N/A | N/A | 883 unknown | 0.93 | |
| A_92_P040816 | N/A | N/A | 2688 UP:Q672R6_MAIZE (Q672R6) Hydroxymethylbutenyl 4-diphosphate synthase, complete | 0.93 | |
| A_92_P042032 | N/A | N/A | 1225 homologue to UP:Q656T5_ORYSA (Q656T5) Oxidoreductase-like, partial (86%) | 0.92 | |
| A_92_P003311 | N/A | N/A | 1553 Zea mays clone EK07D2312C08.c mRNA sequence | 0.92 | |
| A_92_P023817 | N/A | N/A | 1296 weakly similar to PRF:NP_199714.2:22327660:NP_199714 tRNA synthetase class I (I, L, M and V) family protein (Arabidopsis thaliana), partial (20%) | 0.91 | |
| A_92_P001858 | N/A | N/A | NP_001106257 ZCN25 protein [Zea mays] | 0.91 | |
| A_92_P010331 | N/A | N/A | 2396 homologue to UP:Q8L8I4_ORYSA (Q8L8I4) RNase L inhibitor-like protein, complete | 0.91 | |
| A_92_P023614 | N/A | N/A | 1321 similar to PRF:NP_565610.1:18400953:NP_565610 YebC-related (Arabidopsis thaliana), partial (77%) | 0.91 | |
| A_92_P018531 | N/A | N/A | ACG42765 lysosomal protective protein precursor [Zea mays] | 0.90 | |
| A_92_P005979 | N/A | N/A | 877 homologue to UP:Q8LP98_ORYSA (Q8LP98) Transcription factor PCF3 (Fragment), partial (12%) | 0.90 | |
| A_92_P017003 | N/A | N/A | 580 unknown | 0.90 | |
| A_92_P035799 | N/A | N/A | 1017 weakly similar to PRF:NP_567701.1:18416334:NP_567701 expressed protein (Arabidopsis thaliana), partial (81%) | 0.90 | |
| A_92_P010913 | N/A | N/A | 322 unknown | 0.89 | |
| A_92_P028419 | N/A | N/A | 2033 weakly similar to UP:Q84M90_ARATH (Q84M90) At3g15180, partial (33%) | 0.89 | |
| A_92_P005849 | N/A | N/A | 1419 Zea mays clone EL01N0449B09.c mRNA sequence | 0.89 | |
| A_92_P039106 | N/A | N/A | 1125 Zea mays clone EL01N0551G08.c mRNA sequence | 0.89 | |
| A_92_P012657 | N/A | N/A | 963 similar to PRF:NP_199714.2:22327660:NP_199714 tRNA synthetase class I (I, L, M and V) family protein (Arabidopsis thaliana), partial (29%) | 0.89 | |
| A_92_P008984 | N/A | N/A | 788 unknown | 0.88 | |
| A_92_P034436 | N/A | N/A | 749 similar to GB:AAS07143.1:41469239:AC145381 expressed protein (Oryza sativa (japonica cultivar-group)), partial (18%) | 0.88 | |
| A_92_P028610 | N/A | N/A | 1546 weakly similar to PRF:NP_564287.1:18396370:NP_564287 expressed protein (Arabidopsis thaliana), partial (24%) | 0.88 | |
| A_92_P036412 | N/A | N/A | 726 similar to PRF:NP_190247.1:15232596:NP_190247 expressed protein (Arabidopsis thaliana), partial (55%) | 0.88 | |
| A_92_P005056 | N/A | N/A | ACG44343 AER [Zea mays] | 0.88 | |
| A_92_P014585 | N/A | N/A | EEF47825 kinase, putative [Ricinus communis] | 0.87 | |
| A_92_P030533 | N/A | N/A | 234 homologue to UP:Q656T5_ORYSA (Q656T5) Oxidoreductase-like, partial (8%) | 0.87 | |
| A_92_P014141 | N/A | N/A | 356 weakly similar to PRF:NP_175994.1:15222815:NP_175994 expressed protein (Arabidopsis thaliana), partial (14%) | 0.87 | |
| A_92_P020446 | N/A | N/A | 752 similar to PRF:NP_568306.1:18417512:NP_568306 heavy-metal-associated domain-containing protein (Arabidopsis thaliana), partial (31%) | 0.87 | |
| A_92_P025303 | N/A | N/A | CAA32268 petD [Hordeum vulgare subsp. vulgare] | 0.87 | |
| A_92_P005960 | N/A | N/A | 2616 telomerase reverse transcriptase catalytic subunit [Zea mays] | 0.85 | |
| A_92_P008365 | N/A | N/A | 1324 similar to UP:Q58IJ7_HORVU (Q58IJ7) UDP-D-glucose epimerase 3, partial (95%) | 0.85 | |
| A_92_P024795 | N/A | N/A | 867 weakly similar to PRF:NP_563709.1:18390404:NP_563709 expressed protein (Arabidopsis thaliana), partial (16%) | 0.85 | |
| A_92_P004298 | N/A | N/A | 820 Zea mays clone EL01N0551C01.c mRNA sequence | 0.84 | |
| A_92_P036647 | N/A | N/A | 1249 similar to PRF:NP_195914.2:30679833:NP_195914 expressed protein (Arabidopsis thaliana), partial (31%) | 0.84 | |
| A_92_P031952 | N/A | N/A | 841 unknown | 0.84 | |
| A_92_P022195 | N/A | N/A | 1284 similar to PRF:NP_564513.1:18401869:NP_564513 expressed protein (Arabidopsis thaliana), partial (52%) | 0.83 | |
| A_92_P037006 | N/A | N/A | 644 similar to UP:Q5CZ54_SOLTU (Q5CZ54) Pom14 protein, partial (53%) | 0.83 | |
| A_92_P007245 | N/A | N/A |  | 0.83 | |
| A_92_P035543 | N/A | N/A | 1080 similar to PRF:NP_189354.2:42565237:NP_189354 expressed protein (Arabidopsis thaliana), partial (32%) | 0.83 | |
| A_92_P000258 | N/A | N/A | 638 homologue to UP:Q6L724_HORVU (Q6L724) ATP-dependent RNA helicase, partial (27%) | 0.83 | |
| A_92_P028842 | N/A | N/A | ACG35967 retrotransposon protein SINE subclass [Zea mays] | 0.83 | |
| A_92_P034585 | N/A | N/A | 958 similar to PRF:NP_564287.1:18396370:NP_564287 expressed protein (Arabidopsis thaliana), partial (12%) | 0.83 | |
| A_92_P003523 | N/A | N/A | 623 homologue to UP:Q8L8I4_ORYSA (Q8L8I4) RNase L inhibitor-like protein, partial (29%) | 0.83 | |
| A_92_P015040 | N/A | N/A | 1022 weakly similar to PRF:NP_196828.2:22326767:NP_196828 expressed protein (Arabidopsis thaliana), partial (92%) | 0.83 | |
| A_92_P007901 | N/A | N/A | 1742 Zea mays clone Contig117 mRNA sequence | 0.82 | |
| A_92_P001516 | N/A | N/A | 1372 unknown | 0.82 | |
| A_92_P039122 | N/A | N/A | 861 similar to UP:Q94JV0_ARATH (Q94JV0) At1g69210:F4N2_11, partial (19%) | 0.82 | |
| A_92_P018708 | N/A | N/A | 2393 similar to UP:Q9M4Q0_HORVU (Q9M4Q0) Molybdenum cofactor biosynthesis protein Cnx1, partial (97%) | 0.81 | |
| A_92_P017691 | N/A | N/A | EEF40901 lrr receptor-linked protein kinase, putative [Ricinus communis] | 0.81 | |
| A_92_P033874 | N/A | N/A | YP_002135627 methyl-accepting chemotaxis sensory transducer [Anaeromyxobacter sp. K] | 0.81 | |
| A_92_P001925 | N/A | N/A | 734 similar to UP:Q75GL2_ORYSA (Q75GL2) Expressed protein (With alternative splicing), partial (14%) | 0.81 | |
| A_92_P024716 | N/A | N/A | 615 weakly similar to UP:PRT2_ONCMY (P02330) Protamine II (Iridine II), partial (66%) | 0.81 | |
| A_92_P039610 | N/A | N/A | YP_008997 putative 23S rRNA (Uracil-5-)-methyltransferase [Candidatus Protochlamydia amoebophila UWE25] | 0.81 | |
| A_92_P040402 | N/A | N/A | 1084 similar to UP:Q9ZTU8_WHEAT (Q9ZTU8) S276, partial (40%) | 0.81 | |
| A_92_P010440 | N/A | N/A | 871 homologue to UP:Q6YW48_ORYSA (Q6YW48) Zinc finger protein-like, partial (10%) | 0.81 | |
| A_92_P012980 | N/A | N/A | 1031 GB:AAB30283.2:13449983:S69194S3 IRF170 (Zea mays), partial (54%) | 0.81 | |
| A_92_P008359 | N/A | N/A | 1354 UP:Q6ZHA3_ORYSA (Q6ZHA3) Small GTP-binding protein RACBP, complete | 0.80 | |
| A_92_P011771 | N/A | N/A | 1002 similar to PRF:NP_199714.2:22327660:NP_199714 tRNA synthetase class I (I, L, M and V) family protein (Arabidopsis thaliana), partial (25%) | 0.80 | |
| A_92_P007278 | N/A | N/A | 2023 similar to UP:Q9ZTU8_WHEAT (Q9ZTU8) S276, partial (95%) | 0.80 | |
| A_92_P000940 | N/A | N/A | 868 similar to PRF:NP_199967.1:15242116:NP_199967 peptidase M3 family protein : thimet oligopeptidase family protein (Arabidopsis thaliana), partial (8%) | 0.80 | |
| A_92_P012302 | N/A | N/A | 1099 similar to PRF:NP_176001.2:42562778:NP_176001 GTP-binding protein-related (Arabidopsis thaliana), partial (66%) | 0.80 | |
| A_92_P028341 | N/A | N/A | 1723 similar to UP:Q9FYR6_ARATH (Q9FYR6) Prolyl tRNA synthetase (At5g52520), partial (88%) | 0.80 | |
| A_92_P003384 | N/A | N/A | CAC01785 Carboxylesterase-like protein [Arabidopsis thaliana] | 0.79 | |
| A_92_P004401 | N/A | N/A | AAF78485 Contains similarity to S1 protein from Homo sapiens gb\|U27517 and contains a S1 RNA binding PF\|00575 domain. EST gb\|F15427, gb\|F15428 comes from this gene. [Arabidopsis thaliana] | 0.79 | |
| A_92_P030327 | N/A | N/A | 1031 GB:AAB30283.2:13449983:S69194S3 IRF170 (Zea mays), partial (54%) | 0.79 | |
| A_92_P027967 | N/A | N/A | 1382 homologue to UP:Q7EAG4_ORYSA (Q7EAG4) Dmc1 protein type B, complete | 0.79 | |
| A_92_P010803 | N/A | N/A | 1485 weakly similar to PRF:NP_194669.1:15233566:NP_194669 expressed protein (Arabidopsis thaliana), partial (69%) | 0.78 | |
| A_92_P007738 | N/A | N/A | ZP_01689674 cell wall-associated hydrolase [Microscilla marina ATCC 23134] | 0.78 | |
| A_92_P010494 | N/A | N/A | 1799 similar to GB:AAS76771.1:45773934:BT012284 At3g26932 (Arabidopsis thaliana), partial (44%) | 0.78 | |
| A_92_P027310 | N/A | N/A | 959 similar to UP:Q94AW2_ARATH (Q94AW2) AT5g39590:MIJ24_60, partial (25%) | 0.78 | |
| A_92_P039423 | N/A | N/A | 923 similar to UP:Q7XIW7_ORYSA (Q7XIW7) Myosin heavy chain-like, partial (35%) | 0.77 | |
| A_92_P008375 | N/A | N/A | 1350 weakly similar to PRF:NP_567745.1:18416739:NP_567745 antitermination NusB domain-containing protein (Arabidopsis thaliana), partial (63%) | 0.77 | |
| A_92_P028344 | N/A | N/A | 2912 similar to UP:Q5JMB8_ORYSA (Q5JMB8) Calmodulin-binding family protein-like, partial (69%) | 0.77 | |
| A_92_P041536 | N/A | N/A | 557 similar to PRF:NP_568580.1:18421967:NP_568580 metal-dependent phosphohydrolase HD domain-containing protein (Arabidopsis thaliana), partial (12%) | 0.77 | |
| A_92_P028480 | N/A | N/A | 633 weakly similar to PRF:NP_198034.2:22327094:NP_198034 ferroportin-related (Arabidopsis thaliana), partial (17%) | 0.77 | |
| A_92_P020541 | N/A | N/A | 1305 similar to PRF:NP_565590.1:18400785:NP_565590 expressed protein (Arabidopsis thaliana), complete | 0.77 | |
| A_92_P016565 | N/A | N/A | 1305 weakly similar to PRF:NP_564471.1:18400085:NP_564471 expressed protein (Arabidopsis thaliana), partial (50%) | 0.76 | |
| A_92_P020703 | N/A | N/A | 1241 similar to GB:BAC24810.1:25553534:AP002805 contains ESTs C72127(E1041),AU078646(E1041) nodulin-like protein (Oryza sativa (japonica cultivar-group)), partial (92%) | 0.76 | |
| A_92_P026383 | N/A | N/A | 1648 weakly similar to PRF:NP_566302.1:18397912:NP_566302 expressed protein (Arabidopsis thaliana), partial (36%) | 0.76 | |
| A_92_P032459 | N/A | N/A | 2396 homologue to UP:Q8L8I4_ORYSA (Q8L8I4) RNase L inhibitor-like protein, complete | 0.76 | |
| A_92_P031683 | N/A | N/A | 1296 weakly similar to UP:ECH1_HUMAN (Q13011) Delta3,5-delta2,4-dienoyl-CoA isomerase, mitochondrial precursor , partial (9%) | 0.76 | |
| A_92_P026875 | N/A | N/A | 2160 Zea mays clone Contig443 mRNA sequence | 0.76 | |
| A_92_P029877 | N/A | N/A | 1226 Zea mays clone EL01T0202B06.c mRNA sequence | 0.76 | |
| A_92_P040175 | N/A | N/A | 1286 similar to UP:Q69K07_ORYSA (Q69K07) Ribosomal large subunit pseudouridine synthase C-like, partial (65%) | 0.76 | |
| A_92_P020612 | N/A | N/A | 219 homologue to UP:O23761_ARATH (O23761) MEtRS , partial (12%) | 0.76 | |
| A_92_P034544 | N/A | N/A | 891 similar to PRF:NP_850290.1:30687357:NP_850290 expressed protein (Arabidopsis thaliana), partial (28%) | 0.75 | |
| A_92_P031844 | N/A | N/A | 1333 similar to UP:Q8S984_ORYSA (Q8S984) Arabidopsis ETTIN-like protein 2, partial (35%) | 0.75 | |
| A_92_P038651 | N/A | N/A | 550 similar to UP:Q67YS7_ARATH (Q67YS7) MRNA, complete cds, clone: RAFL24-10-D10, partial (11%) | 0.75 | |
| A_92_P025816 | N/A | N/A | 1479 Zea mays clone Contig495.F mRNA sequence | 0.75 | |
| A_92_P037482 | N/A | N/A |  | 0.75 | |
| A_92_P002389 | N/A | N/A | 606 unknown | 0.75 | |
| A_92_P033905 | N/A | N/A | 2197 Zea mays clone EL01N0553C01.d mRNA sequence | 0.75 | |
| A_92_P031899 | N/A | N/A | 1382 weakly similar to UP:Q9LL85_SOLTU (Q9LL85) DNA-binding protein p24, partial (65%) | 0.74 | |
| A_92_P040648 | N/A | N/A | XP_002313020 inner membrane protein [Populus trichocarpa] | 0.74 | |
| A_92_P035921 | N/A | N/A | EEF50694 Protease, putative [Ricinus communis] | 0.74 | |
| A_92_P030725 | N/A | N/A | 1847 Zea mays clone EL01T0204A05.d mRNA sequence | 0.74 | |
| A_92_P009744 | N/A | N/A | 1175 similar to UP:Q6L613_AEGTA (Q6L613) WNdr1D-like protein kinase, partial (53%) | 0.74 | |
| A_92_P020399 | N/A | N/A | 762 similar to GB:AAL37489.1:17225592:AF331847 serine acetyltransferase (Arabidopsis thaliana), partial (22%) | 0.73 | |
| A_92_P004650 | N/A | N/A | 1147 similar to UP:Q84MH1_ORYSA (Q84MH1) Expressed protein, partial (38%) | 0.73 | |
| A_92_P027950 | N/A | N/A | 1465 similar to UP:Q6SQN4_ORYSA (Q6SQN4) Circadian oscillator component, partial (44%) | 0.73 | |
| A_92_P016845 | N/A | N/A | 1808 weakly similar to UP:Q944A0_ARATH (Q944A0) AT5g22820:MRN17_5, partial (11%), binding | 0.73 | |
| A_92_P024103 | N/A | N/A | 1146 similar to UP:Q6L724_HORVU (Q6L724) ATP-dependent RNA helicase, partial (29%) | 0.73 | |
| A_92_P003480 | N/A | N/A | 988 similar to PRF:NP_566205.1:18396541:NP_566205 Ku70-binding family protein (Arabidopsis thaliana), partial (65%) | 0.73 | |
| A_92_P001107 | N/A | N/A | 1155 similar to UP:Q6FUA8_CANGA (Q6FUA8) Candida glabrata strain CBS138 chromosome F complete sequence, partial (6%) | 0.73 | |
| A_92_P016685 | N/A | N/A | 1205 similar to UP:HIS2_ARATH (O82768) Histidine biosynthesis bifunctional protein hisIE, chloroplast precursor [Includes: Phosphoribosyl-AMP cyclohydrolase (PRA-CH) | 0.72 | |
| A_92_P006001 | N/A | N/A | 2201 similar to PRF:NP_198037.2:30690432:NP_198037 expressed protein (Arabidopsis thaliana), partial (12%) | 0.72 | |
| A_92_P041511 | N/A | N/A | 702 similar to UP:Q6YZC9_ORYSA (Q6YZC9) Uridylyl transferase-like, partial (26%) | 0.72 | |
| A_92_P004409 | N/A | N/A | EEF41815 cytosolic purine 5-nucleotidase, putative [Ricinus communis] | 0.72 | |
| A_92_P041316 | N/A | N/A | 662 unknown | 0.72 | |
| A_92_P034506 | N/A | N/A | 2297 weakly similar to UP:O24293_PEA (O24293) Chloroplast inner envelope protein, 110 kD (IEP110) precursor, partial (32%) | 0.72 | |
| A_92_P035708 | N/A | N/A | ABF95548 cyclin, putative, expressed [Oryza sativa (japonica cultivar-group)] | 0.72 | |
| A_92_P027087 | N/A | N/A | 1768 similar to PRF:NP_974731.1:42573269:NP_974731 aminotransferase class I and II family protein (Arabidopsis thaliana), partial (71%) | 0.71 | |
| A_92_P015431 | N/A | N/A | 2125 Zea mays clone EL01N0432E12.c mRNA sequence | 0.71 | |
| A_92_P012485 | N/A | N/A | 935 similar to UP:Q93YF1_TOBAC (Q93YF1) Nucleic acid binding protein, partial (29%) | 0.71 | |
| A_92_P023560 | N/A | N/A | 706 similar to UP:Q84MA4_ARATH (Q84MA4) At5g07400, partial (3%) forkhead associated | 0.71 | |
| A_92_P025557 | N/A | N/A | XP_521998 PREDICTED: similar to seven transmembrane helix receptor [Pan troglodytes] | 0.70 | |
| A_92_P026091 | N/A | N/A | 690 homologue to UP:Q5FB28_MAIZE (Q5FB28) Chloroplastic iron-superoxide dismutase precursor , partial (84%) | 0.70 | |
| A_92_P004053 | N/A | N/A | 1182 Zea mays clone EL01N0323H03.c mRNA sequence | 0.70 | |
| A_92_P035752 | N/A | N/A | 805 similar to PRF:NP_564623.2:30695448:NP_564623 sodium:calcium exchanger family protein : calcium-binding EF hand family protein (Arabidopsis thaliana), partial (26%) | 0.70 | |
| A_92_P041434 | N/A | N/A | 943 weakly similar to UP:Q8H2U1_ORYSA (Q8H2U1) Selenium-binding protein-like, partial (16%) | 0.70 | |
| A_92_P017437 | N/A | N/A | 1065 similar to UP:Q6STH5_ARATH (Q6STH5) [4Fe-4S] cluster assembly factor, partial (38%) | 0.70 | |
| A_92_P033177 | N/A | N/A | 1290 homologue to UP:Q4KBA9_PSEF5 (Q4KBA9) 4-hydroxyphenylacetate catabolism regulatory protein HpaA, partial (5%) | 0.70 | |
| A_92_P000875 | N/A | N/A | 762 similar to UP:Q9ASP8_ARATH (Q9ASP8) AT3g55400:T22E16_60, partial (16%), OVULE ABORTION 1 | 0.70 | |

Supplementary Table 2. All transcripts found to be significantly down-regulated with a log_2_ fold-change ≥ 0.70 (adj. p-value .00001). Annotations include the Agilent probe ID, MapMan bincode and corresponding bin name, and gene description.

| ID | BINCODE | BIN NAME | GENE DESCRIPTION | LogFC |
| --- | --- | --- | --- | --- |
|  |  |  |  |  |
|  |  |  |  |  |
| **Protein** |  |  |  |  |
|  |  |  |  |  |
| A_92_P015623 | 29.4 | protein.postranslational modification | 1192 similar to UP:Q943R3_ORYSA (Q943R3) Calmodulin-binding protein-like, partial (32%) | -2.39 |
| A_92_P040148 | 29.5.11.04.02 | protein.degradation.ubiquitin.E3.RING | 411 similar to UP:Q655C7_ORYSA (Q655C7) Ring-H2 zinc finger protein-like, partial (7%) | -1.80 |
| A_92_P019384 | 29.5 | protein.degradation | 1935 UP:Q9ZP28_MAIZE (Q9ZP28) C13 endopeptidase NP1 precursor, complete | -1.55 |
| A_92_P012315 | 29.4 | protein.postranslational modification | 2080 weakly similar to UP:Q94C40_ARATH (Q94C40) CBL-interacting protein kinase 17, partial (81%) | -1.24 |
| A_92_P006094 | 29.4 | protein.postranslational modification | 406 weakly similar to UP:P93370_TOBAC (P93370) Calmodulin-binding protein, partial (9%) | -1.06 |
| A_92_P010324 | 29.5.11.04.02 | protein.degradation.ubiquitin.E3.RING | similar to UP\|Q5GAQ1 MAIZE (Q5GAQ1) Ring-H2 zinc finger protein, partial (38%) | -1.02 |
| A_92_P005471 | 29.4 | protein.postranslational modification | ABA98870 calmodulin-binding protein, putative, expressed [Oryza sativa (japonica cultivar-group)] | -0.99 |
| A_92_P020974 | 29.4 | protein.postranslational modification | 2080 weakly similar to UP:Q94C40_ARATH (Q94C40) CBL-interacting protein kinase 17, partial (81%) | -0.98 |
| A_92_P001627 | 29.5.11.04.03.02 | protein.degradation.ubiquitin.E3.SCF.FBOX | 1035 weakly similar to UP:Q5VR67_ORYSA (Q5VR67) F-box family protein-like, partial (44%) | -0.98 |
| A_92_P037353 | 29.5.11.03 | protein.degradation.ubiquitin.E2 | 1002 similar to UP:Q6YKA6_PAVLU (Q6YKA6) Ubiquitin-conjugating enzyme E2, partial (66%) | -0.87 |
| A_92_P016716 | 29.4 | protein.postranslational modification | 1508 similar to PRF:NP_194348.1:15236140:NP_194348 mitochondrial substrate carrier family protein (Arabidopsis thaliana), partial (85%) | -0.84 |
| A_92_P007118 | 29.5.11 | protein.degradation.ubiquitin | 701 homologue to PRF:NP_190104.1:15230632:NP_190104 ubiquitin family protein (Arabidopsis thaliana), complete | -0.84 |
| A_92_P031338 | 29.5.11.04.03.02 | protein.degradation.ubiquitin.E3.SCF.FBOX | 662 similar to UP:Q9SDA8_ARATH (Q9SDA8) At2g17020 (At2g17020:At2g17020), partial (8%), F-BOX family protein FBL10 | -0.83 |
| A_92_P040397 | 29.4 | protein.postranslational modification | 940 homologue to UP:Q9ATP2_PENCL (Q9ATP2) Calmodulin-like protein, partial (96%) | -0.79 |
| A_92_P026402 | 29.2.4 | protein.synthesis.elongation | 1229 UP:SUI1_MAIZE (P56330) Protein translation factor SUI1 homolog (GOS2 protein), complete | -0.77 |
| A_92_P002413 | 29.5.11.04.02 | protein.degradation.ubiquitin.E3.RING | 1306 weakly similar to UP:Q6ZHB2_ORYSA (Q6ZHB2) S-ribonuclease binding protein SBP1-like, partial (32%) | -0.72 |
|  |  |  |  |  |
|  |  |  |  |  |
| **Regulation of Transcription** | | |  |  |
|  |  |  |  |  |
| A_92_P009010 | 27.3.32 | RNA.regulation of transcription.WRKY domain transcription factor family | 1418 similar to UP:Q6B6Q9_ORYSA (Q6B6Q9) Transcription factor WRKY31, partial (41%) | -2.20 |
| A_92_P030158 | 27.3.99 | RNA.regulation of transcription.unclassified | 1414 similar to UP:Q5N8K7_ORYSA (Q5N8K7) Aspartic proteinase nepenthesin I-like, partial (49%) | -2.04 |
| A_92_P018974 | 27.3.99 | RNA.regulation of transcription.WRKY domain transcription factor family | 1153 similar to UP:Q84K08_ORYSA (Q84K08) Zinc-finger protein, partial (40%) | -1.53 |
| A_92_P038150 | 27.3.99 | RNA.regulation of transcription.WRKY domain transcription factor family | homologue to UP\|Q73Y19 MYCPA (Q73Y19) FurB, partial (8%) | -1.34 |
| A_92_P019538 | 27.3.03 | RNA.regulation of transcription.AP2/EREBP, APETALA2/Ethylene-responsive element binding protein family | 1343 similar to UP:Q8LKW9_MAIZE (Q8LKW9) DRE binding factor 1, partial (48%) | -1.34 |
| A_92_P032910 | 27.3.32 | RNA.regulation of transcription.WRKY domain transcription factor family | 1260 similar to UP:Q6B6Q8_ORYSA (Q6B6Q8) Transcription factor WRKY32, partial (34%) | -1.20 |
| A_92_P020341 | 27.3.11 | RNA.regulation of transcription.C2H2 zinc finger family | 975 similar to UP:Q6ZII7_ORYSA (Q6ZII7) Zinc finger and C2 domain protein-like, complete | -1.08 |
| A_92_P021296 | 27.3.99 | RNA.regulation of transcription.unclassified | 2113 similar to UP:Q6Z5I2_ORYSA (Q6Z5I2) Zinc finger (C3HC4-type RING finger)-like protein, partial (28%) | -1.04 |
| A_92_P019648 | 27.3.67 | RNA.regulation of transcription.putative DNA-binding protein | 1132 similar to UP:REMO_SOLTU (P93788) Remorin (pp34), partial (80%) | -0.99 |
| A_92_P016747 | 27.3 | RNA.regulation of transcription | 1548 UP:Q93WI2_MAIZE (Q93WI2) Teosinte branched1 protein (Fragment), complete | -0.87 |
| A_92_P014828 | 27.3.99 | RNA.regulation of transcription.unclassified | 1496 weakly similar to UP:TNKS1_HUMAN (O95271) Tankyrase 1 (TANK1) (Tankyrase I) (TNKS-1) (TRF1-interacting ankyrin-related ADP-ribose polymerase) , partial (3%) | -0.84 |
| A_92_P017500 | 27.3.32 | RNA.regulation of transcription.WRKY domain transcription factor family | 1496 similar to UP:Q94EA8_ORYSA (Q94EA8) WRKY transcription factor 14 (WRKY14), partial (56%) | -0.80 |
| A_92_P027733 | 27.3.29 | RNA.regulation of transcription.TCP transcription factor family | ACG42335 TCP family transcription factor containing protein [Zea mays] | -0.78 |
| A_92_P026052 | 27.3.08 | RNA.regulation of transcription.C2C2(Zn) DOF zinc finger family | 1899 similar to UP:DOF22_ARATH (Q9ZV33) Dof zinc finger protein DOF2.2 (AtDOF2.2), partial (25%) | -0.73 |
| A_92_P013435 | 27.3.25 | RNA.regulation of transcription.MYB domain transcription factor family | 1355 R2R3MYB-domain protein | -0.7021 |
|  |  |  |  |  |
|  |  |  |  |  |
| **RNA Processing** | |  |  |  |
|  |  |  |  |  |
| A_92_P009279 | 27.1 | RNA Processing | 643 weakly similar to UP:O23646_ARATH (O23646) RSZp22 protein, partial (12%) | -1.09 |
| A_92_P025593 | 27.1 | RNA.processing | 503 weakly similar to UP:FBRL_HUMAN (P22087) Fibrillarin (34 kDa nucleolar scleroderma antigen), partial (12%) | -0.84 |
| A_92_P031972 | 27.1 | RNA.processing | 2143 weakly similar to UP:FBRL_SCHPO (P35551) Fibrillarin, partial (8%) | -0.76 |
|  |  |  |  |  |
|  |  |  |  |  |
| **Transport** |  |  |  |  |
|  |  |  |  |  |
| A_92_P028076 | 34.16 | transport.ABC transporters and multidrug resistance systems | 547 similar to UP:Q8GU66_ORYSA (Q8GU66) MRP-like ABC transporter, partial (7%) | -1.63 |
| A_92_P022084 | 34.3 | transport.amino acids | 549 similar to UP:Q941Z7_ORYSA (Q941Z7) BHLH transcription factor-like, partial (51%) | -1.60 |
| A_92_P011533 | 34.7 | transport.phosphate | 1944 UP:Q49B46_MAIZE (Q49B46) Inorganic phosphate transporter 1, complete | -1.42 |
| A_92_P037740 | 34.12 | transport.metal | 2494 GB:AF186234.2:AAG17016.2 iron-phytosiderophore transporter protein yellow stripe 1 [Zea mays]- Fe(III) transport/uptake membrane protein | -1.29 |
| A_92_P030183 | 34.15 | transport.potassium | weakly similar to UP\|HAK16 ORYSA (Q84MS3) Probable potassium transporter 16 (OsHAK16), partial (18%) | -1.25 |
| A_92_P026621 | 34.4 | transport.nitrate | 2180 weakly similar to PIR:T10255:T10255 nitrite transport protein, chloroplast - cucumber (Cucumis sativus), partial (57%) | -1.07 |
| A_92_P016254 | 34.2 | transporter.sugars | 1012 weakly similar to GB:CAD70577.1:32698459:MMU549317 solute carrier family 2 (facilitated glucose transporter), member 12 (Mus musculus), partial (4%) | -0.99 |
| A_92_P039817 | 34.9 | transport.metabolite transporters at the mitochondrial membrane | 438 GB:CAA33742.1:22166:ZMANTG1 adenine nucleotide translocator (Zea mays), partial (15%) | -0.98 |
| A_92_P000905 | 34.99 | transport misc | 2018 similar to UP:Q9LVD9_ARATH (Q9LVD9) Gb:AAC28507.1 (At3g21690), partial (84%) MATE efflux family protein | -0.95 |
| A_92_P024242 | 34.3 | transport.amino acids | ACG46044 LHT1 [Zea mays] lysine histidine transporter 1 | -0.95 |
| A_92_P008394 | 34.14 | transport.unspecified cations | XP_002325685 equilibrative nucleoside transporter [Populus trichocarpa] | -0.89 |
| A_92_P022176 | 34.16 | transport.ABC transporters and multidrug resistance systems | 1220 homologue to UP:Q6J0P5_MAIZE (Q6J0P5) Multidrug-resistance associated protein 3, partial (18%) | -0.88 |
| A_92_P036313 | 34.9 | transport.metabolite transporters at the mitochondrial membrane | 895 similar to UP:O80413_MAIZE (O80413) Mitochondrial phosphate transporter, partial (53%) | -0.86 |
| A_92_P028355 | 34.15 | transport.potassium | 1098 similar to UP:HAK7_ORYSA (Q8H3P9) Potassium transporter 7 (OsHAK7), partial (31%) | -0.84 |
| A_92_P028019 | 34.12 | transport.metal | 566 similar to UP:Q6H7J6_ORYSA (Q6H7J6) Oligopeptide transporter OPT-like, partial (20%) | -0.75 |
| A_92_P038771 | 34.2 | transporter.sugars | 1135 homologue to UP:Q6VEF2_ORYSA (Q6VEF2) Monosaccharide transporter 4, partial (50%) | -0.73 |
| A_92_P031303 | 34.19.2 | transport.Major Intrinsic Proteins.TIP | 1081 UP:Q9ATL8_MAIZE (Q9ATL8) Tonoplast membrane integral protein ZmTIP2-2, complete | -0.72 |
| A_92_P040408 | 34.98 | transporter.membrane system unknown | 1197 similar to UP:Q9WU81_MOUSE (Q9WU81) CAMP inducible 2 protein (Mus musculus adult male thymus cDNA, RIKEN full-length enriched library, clone:5832433D20 product:solute carrier family 37 (glycerol-3-phosphate transporter), member 1, full insert sequen | -0.72 |
|  |  |  |  |  |
|  |  |  |  |  |
| **Signalling** |  |  |  |  |
|  |  |  |  |  |
| A_92_P017326 | 30.2.12 | signalling.receptor kinases.leucine rich repeat XII | ACA05156 Xa21-like protein [Triticum aestivum] | -1.54 |
| A_92_P039832 | 30.2 | signalling.receptor kinases | ABB84341 resistance-related receptor-like kinase [Triticum aestivum] | -1.49 |
| A_92_P001231 | 30.3 | signalling.calcium | 1395 similar to UP:Q5SND2_ORYSA (Q5SND2) Calmodulin-like protein, partial (75%) | -1.20 |
| A_92_P014921 | 30.11 | signalling.light | 437 homologue to UP:Q84YF6_SORBI (Q84YF6) RPT2 (Root Phototropism 2)-like protein, partial (3%) | -1.09 |
| A_92_P030669 | 30.2.2 | signalling.receptor kinases.leucine rich repeat II | 2354 homologue to UP:Q94IJ5_MAIZE (Q94IJ5) SERK2 protein precursor, complete | -0.97 |
| A_92_P024725 | 30.2.99 | signalling.receptor kinases.misc | 2855 UP:Q53U95_MAIZE (Q53U95) Wound and phytochrome signaling involved receptor like kinase, complete | -0.92 |
| A_92_P029592 | 30.11 | signalling.light | 1462 weakly similar to UP:Q7XYY2_ARATH (Q7XYY2) Phytochrome and flowering time 1 protein, partial (11%) | -0.88 |
| A_92_P036200 | 30.2.99 | signalling.receptor kinases.misc | 1012 similar to UP:Q53U95_MAIZE (Q53U95) Wound and phytochrome signaling involved receptor like kinase, partial (19%) | -0.78 |
| A_92_P006139 | 30.2.17 | signalling.receptor kinases.DUF 26 | 733 similar to UP:Q7F1L5_ORYSA (Q7F1L5) Serine:threonine kinase receptor-like protein, partial (13%) | -0.70 |
|  |  |  |  |  |
|  |  |  |  |  |
| **Signal Transduction** | |  |  |  |
|  |  |  |  |  |
| A_92_P006292 |  | Signal Transduction | 1270 similar to UP:Q5Z661_ORYSA (Q5Z661) Receptor protein kinase-like, partial (54%) | -1.88 |
| A_92_P018299 |  | Signal Transduction | 1284 UP:P93518_MAIZE (P93518) PRm 3 , complete | -1.39 |
| A_92_P026938 |  | Signal Transduction | AAT39269 putative receptor-like protein kinase [Oryza sativa (japonica cultivar-group)] | -1.14 |
| A_92_P014436 |  | Signal Transduction | 873 similar to UP:Q84SH0_ORYSA (Q84SH0) Serine:threonine kinase receptor-like protein, partial (38%) | -1.13 |
| A_92_P028354 |  | Signal Transduction | 609 weakly similar to UP:O49974_MAIZE (O49974) KI domain interacting kinase 1 , partial (4%) | -1.11 |
| A_92_P007191 |  | Signal Transduction | 916 similar to UP:Q5Z7K5_ORYSA (Q5Z7K5) S-receptor kinase-like, partial (20%) | -1.05 |
|  |  |  |  |  |
|  |  |  |  |  |
| **DNA Processing** | |  |  |  |
|  |  |  |  |  |
| A_92_P031085 | 28.1 | DNA.synthesis/chromatin structure | XP_952181 5'-3' exonuclease [Theileria annulata strain Ankara] | -0.80 |
| A_92_P020637 | 28.1 | DNA.synthesis/chromatin structure | 934 similar to UP:Q6NMJ9_ARATH (Q6NMJ9) At2g23840, partial (63%), HNH endonuclease-domain containing protein | -0.78 |
| A_92_P025815 | 28.1 | DNA.synthesis/chromatin structure | 1436 similar to PRF:NP_180594.2:42569467:NP_180594 endo:excinuclease amino terminal domain-containing protein (Arabidopsis thaliana), partial (34%) | -0.78 |
| A_92_P039284 | 28.1.1 | DNA.synthesis/chromatin structure.retrotransposon/transposase | ABF97694 retrotransposon protein, putative, unclassified [Oryza sativa (japonica cultivar-group)] | -0.78 |
|  |  |  |  |  |
|  |  |  |  |  |
| **Nucleotide Metabolism** | |  |  |  |
|  |  |  |  |  |
| A_92_P006313 | 23.1.1.10 | nucleotide metabolism.synthesis.pyrimidine.CTP synthetase | NP_693929 CTP synthetase [Oceanobacillus iheyensis HTE831] | -0.80 |
|  |  |  |  |  |
|  |  |  |  |  |
| **Stress** |  |  |  |  |
|  |  |  |  |  |
| A_92_P010358 | 20.1 | stress.biotic | 1011 homologue to UP:Q4VQB3_SORBI (Q4VQB3) Pathogenesis-related protein 10d, complete | -4.70 |
| A_92_P014043 | 20.1 | stress.biotic | weakly similar to YP_001673854 acriflavin resistance protein [Shewanella halifaxensis HAW-EB4] | -2.10 |
| A_92_P001927 | 20.2 | stress.abiotic | 1378 UP:Q8GT70_MAIZE (Q8GT70) Alternative oxidase AOX3, complete | -1.99 |
| A_92_P014347 | 20.2.1 | stress.abiotic.heat | 957 UP:Q41815_MAIZE (Q41815) Heat shock protein 26, complete | -1.91 |
| A_92_P036065 | 20.2.1 | stress.abiotic.heat | 1011 similar to GB:CAA27330.1:861170:ZMHSP702 heat shock protein 70 (Zea mays), partial (43%) | -1.57 |
| A_92_P023606 | 20.1 | stress.biotic | 769 homologue to UP:PRMS_MAIZE (Q00008) Pathogenesis-related protein PRMS precursor, complete | -1.50 |
| A_92_P018873 | 20.1 | stress.biotic | 1196 similar to UP:Q6IER2_ORYSA (Q6IER2) WRKY transcription factor 19, partial (39%) | -1.31 |
| A_92_P017915 | 20.1 | stress.biotic | 905 weakly similar to UP:Q6V9H6_ORYSA (Q6V9H6) Brown planthopper-induced resistance protein 1, partial (22%) | -1.06 |
| A_92_P005705 | 20.2 | stress.abiotic | 1488 similar to UP:TIP1_YEAST (P27654) Temperature-shock inducible protein 1 precursor , partial (9%) | -1.04 |
| A_92_P000408 | 20.2 | stress.abiotic | 592 similar to UP:Q52QX9_MANES (Q52QX9) Aldo:keto reductase AKR, partial (39%) | -1.03 |
| A_92_P024944 | 20.2.1 | stress.abiotic.heat | 1544 homologue to UP:Q5Z9N8_ORYSA (Q5Z9N8) Heat shock protein 90, partial (54%) | -0.96 |
| A_92_P006793 | 20.1 | stress.biotic | 958 similar to UP:Q69RN2_ORYSA (Q69RN2) Chitinase III-like protein, partial (98%) | -0.90 |
| A_92_P016081 | 20.2.1 | stress.abiotic.heat | 1313 similar to PRF:NP_188036.1:15231803:NP_188036 DNAJ heat shock N-terminal domain-containing protein (Arabidopsis thaliana), partial (21%) | -0.84 |
| A_92_P001321 | 20.1 | stress.biotic | 765 similar to UP:O82086_MAIZE (O82086) Pathogenesis related protein-1, partial (85%) | -0.82 |
| A_92_P016088 | 20.1 | stress.biotic | 1240 homologue to UP:Q4NPG7_9DELT (Q4NPG7) PE-PGRS family protein, partial (3%) | -0.81 |
| A_92_P019440 | 20.2.1 | stress.abiotic.heat | 516 homologue to UP:Q5Z9N8_ORYSA (Q5Z9N8) Heat shock protein 90, partial (17%) | -0.80 |
| A_92_P024708 | 20.1 | stress.biotic | 4882 UP:Q6Y3I1_MAIZE (Q6Y3I1) Multidrug resistance associated protein 1, complete | -0.77 |
| A_92_P016866 | 20.2.1 | stress.abiotic.heat | 1400 similar to PRF:NP_565982.1:18406052:NP_565982 DNAJ heat shock N-terminal domain-containing protein (Arabidopsis thaliana), partial (70%) | -0.73 |
|  |  |  |  |  |
|  |  |  |  |  |
| **Hormone Metabolism** | |  |  |  |
|  |  |  |  |  |
| A_92_P011791 | 17.4.1 | hormone metabolism.cytokinin.synthesis-degradation | 1360 weakly similar to UP:CKX7_ARATH (Q9FUJ1) Cytokinin dehydrogenase 7 (Cytokinin oxidase 7) (CKO7) (AtCKX7) (AtCKX5) , partial (19%) | -1.73 |
| A_92_P040776 | 17.1.1 | hormone metabolism.abscisic acid.synthesis-degradation | 521 similar to UP:Q44GA7_CHRSL (Q44GA7) Aldehyde oxidase and xanthine dehydrogenase, a:b hammerhead:Aldehyde oxidase and xanthine dehydrogenase, molybdopterin binding, partial (3%) | -1.30 |
| A_92_P021777 | 17.6.3 | hormone metabolism.gibberelin.induced-regulated-responsive-activated | 1820 similar to UP:Q9AS97_ORYSA (Q9AS97) Gibberellin response modulator-like, partial (79%) | -1.21 |
| A_92_P011313 | 17.2.1 | hormone metabolism.auxin.synthesis-degradation | 903 similar to PRF:NP_567276.1:18412757:NP_567276 amidohydrolase family protein (Arabidopsis thaliana), partial (35%) | -1.12 |
| A_92_P041713 | 17.6.1 | hormone metabolism.gibberelin.synthesis-degradation | ACG29607 ent-kaurene synthase B [Zea mays] | -0.93 |
| A_92_P020866 | 17.03.01.02 | hormone metabolism.brassinosteroid.synthesis-degradation.sterols | 1446 similar to UP:Q6YWY7_ORYSA (Q6YWY7) Sterol desaturase-like, partial (98%) | -0.83 |
| A_92_P036394 | 17.6.1 | hormone metabolism.gibberelin.synthesis-degradation | 1702 similar to UP:Q8S0S6_ORYSA (Q8S0S6) Gibberellin 2-oxidase, partial (46%) | -0.78 |
| A_92_P007859 | 17.7.1.2 | hormone metabolism.jasmonate.synthesis-degradation.lipoxygenase | 893 similar to UP:LOX4_ORYSA (Q53RB0) Probable lipoxygenase 4 , partial (23%) | -0.77 |
| A_92_P005060 | 17.5.1 | hormone metabolism.ethylene.synthesis-degradation | 1390 UP:Q6JN53_MAIZE (Q6JN53) Acc oxidase, complete | -0.71 |
| A_92_P032273 | 17.1.1 | hormone metabolism.abscisic acid.synthesis-degradation | 530 homologue to UP:O23888_MAIZE (O23888) Aldehyde oxidase-2 , partial (9%) | -0.71 |
|  |  |  |  |  |
|  |  |  |  |  |
| **Secondary Metabolism** | |  |  |  |
|  |  |  |  |  |
| A_92_P035736 | 16.2 | secondary metabolism.phenylpropanoids | 1391 similar to UP:ZRP4_MAIZE (P47917) O-methyltransferase ZRP4 (OMT) , partial (69%) | -2.03 |
| A_92_P004468 | 16.1 | secondary metabolism.isoprenoids | NP_001105855 terpene synthase 3 [Zea mays] | -1.49 |
| A_92_P019259 | 16.1 | secondary metabolism.isoprenoids | 1917 UP:Q84ZW8_MAIZE (Q84ZW8) Terpene synthase, complete | -1.38 |
| A_92_P003968 | 16.7 | secondary metabolism.wax | ABL11231 putative b-keto acyl reductase [Hordeum vulgare subsp. vulgare] | -1.09 |
| A_92_P014228 | 16.2.1.03 | secondary metabolism.phenylpropanoids.lignin biosynthesis.4CL | 2110 UP:Q6Q297_MAIZE (Q6Q297) 4-coumarate coenzyme A ligase, complete | -0.86 |
| A_92_P011443 | 16.8.4 | secondary metabolism.flavonoids.flavonols | 1245 homologue to UP:Q43262_MAIZE (Q43262) Flavanone 3-beta-hydroxylase , complete | -0.81 |
| A_92_P005089 | 16.1.2 | secondary metabolism.isoprenoids.mevalonate pathway | 1534 similar to UP:Q8L7R2_ARATH (Q8L7R2) 3-hydroxy-3-methylglutaryl-coenzyme A reductase 2, partial (77%) | -0.71 |
| A_92_P029136 | 16.2 | secondary metabolism.phenylpropanoids | ACG47332 AMP-binding protein [Zea mays] | -0.71 |
|  |  |  |  |  |
|  |  |  |  |  |
| **Amino Acid Metabolism** | |  |  |  |
|  |  |  |  |  |
| A_92_P041131 | 13.2.3.1 | amino acid metabolism.degradation.aspartate family.asparagine | 1369 similar to UP:ASPG_LUPLU (Q9ZSD6) L-asparaginase (L-asparagine amidohydrolase) , partial (86%) | -1.05 |
| A_92_P016888 | 13.1.5.3.01 | amino acid metabolism.synthesis.serine-glycine-cysteine group.cysteine.OASTL | 1449 UP:CYSK_MAIZE (P80608) Cysteine synthase (O-acetylserine sulfhydrylase) (O-acetylserine (Thiol)-lyase) (CSase) (OAS-TL) , complete | -0.90 |
|  |  |  |  |  |
|  |  |  |  |  |
| **Lipid Metabolism** | |  |  |  |
|  |  |  |  |  |
| A_92_P012703 | 11.7 | lipid metabolism.unassigned | similar to RF\|NP 180232.1\|15225767\|NM 128221 acyltransferase {Arabidopsis thaliana} (exp=-1; wgp=0; cg=0), partial (38%) | -1.22 |
| A_92_P006702 | 11.9.3 | lipid metabolism.lipid degradation.lysophospholipases | 2306 UP:Q6QJ78_MAIZE (Q6QJ78) Phospholipase C, complete | -1.07 |
| A_92_P027951 | 11.8.1 | lipid metabolism.'exotics' (steroids, squalene etc).sphingolipids | 787 similar to UP:Q852R1_LOTJA (Q852R1) Serine palmitoyltransferase, partial (30%) | -1.00 |
| A_92_P036185 | 11.3 | lipid metabolism.Phospholipid synthesis | 1163 similar to UP:Q84XL8_9LILI (Q84XL8) Digalactosyldiacylglycerol synthase (Fragment), partial (54%) | -0.91 |
| A_92_P033076 | 11.5 | lipid metabolism.glyceral metabolism | 1845 similar to UP:Q6RJ33_9LILI (Q6RJ33) Glycerol kinase , partial (97%) | -0.79 |
|  |  |  |  |  |
|  |  |  |  |  |
| **Cell Wall** |  |  |  |  |
|  |  |  |  |  |
| A_92_P017506 | 10.3 | cell wall.hemicellulose synthesis | 708 weakly similar to UP:Q9SYK9_ARATH (Q9SYK9) F3F20.13 protein (At1g05680), partial (27%) UDP-glucoronosyl/UDP-glucosyl transferase | -0.91 |
| A_92_P015829 | 10.3 | cell wall.hemicellulose synthesis | 1725 weakly similar to UP:UDB2_RAT (P08541) UDP-glucuronosyltransferase 2B2 precursor (UDPGT) (3-hydroxyandrogen specific) (UDPGTR-4) (RLUG23) , partial (7%) | -0.88 |
| A_92_P000704 | 10.6.3 | cell wall.degradation.pectate lyases and polygalacturonases | 1376 similar to UP:PGIP1_ORYSA (Q8GT95) Polygalacturonase inhibitor 1 precursor (Polygalacturonase-inhibiting protein) (Floral organ regulator 1), partial (96%) | -0.87 |
| A_92_P013700 | 10.4 | cell wall.pectin synthesis | 977 similar to PRF:NP_564983.1:18409445:NP_564983 glycosyl transferase family 8 protein (Arabidopsis thaliana), partial (19%) | -0.86 |
| A_92_P007780 | 10.4 | cell wall.pectin synthesis | 727 similar to UP:Q5JM51_ORYSA (Q5JM51) Glycosyl transferase family 8 protein-like, partial (25%) | -0.77 |
| A_92_P018065 | 10.7 | cell wall.modification | 1317 similar to UP:Q6QFA6_WHEAT (Q6QFA6) Expansin EXPA7, partial (96%) | -0.76 |
| A_92_P012268 | 10.2 | cell wall.cellulose synthesis | 1499 similar to UP:Q69F98_PHAVU (Q69F98) Phytochelatin synthetase-like protein, partial (62%) | -0.75 |
| A_92_P036761 | 10.2 | cell wall.cellulose synthesis | ACG29048 CSLE6 - cellulose synthase-like family E [Zea mays] | -0.72 |
| A_92_P038168 | 10.5.1 | cell wall.cell wall proteins.AGPs | 1376 similar to UP:Q5N9U4_ORYSA (Q5N9U4) Endosperm specific protein-like, partial (70%) | -0.71 |
|  |  |  |  |  |
|  |  |  |  |  |
| **Development** | |  |  |  |
|  |  |  |  |  |
| A_92_P015377 | 33.99 | development.unspecified | 1525 similar to UP:Q8S4X0_PEA (Q8S4X0) Embryo-abundant protein EMB, partial (25%) | -1.74 |
| A_92_P030069 | 33.99 | development.unspecified | 1265 homologue to UP:IN21_MAIZE (P49248) IN2-1 protein, complete | -1.37 |
| A_92_P023149 | 33.99 | development.unspecified | 1083 similar to UP:Q5N8J1_ORYSA (Q5N8J1) MtN3-like, complete | -1.13 |
| A_92_P018803 | 33.88 | development.unspecified | ACG30167 sec12-like protein 1 [Zea mays] | -0.86 |
|  |  |  |  |  |
|  |  |  |  |  |
| **Cell Organization** | |  |  |  |
|  |  |  |  |  |
| A_92_P014061 | 31.1 | cell.organization | 2107 homologue to UP:Q9FUS4_SETIT (Q9FUS4) Actin, complete | -1.07 |
| A_92_P029926 | 31.1 | cell.organisation | 1620 similar to UP:Q84VU1_DAUCA (Q84VU1) 65kD microtubule associated protein, partial (58%) | -1.06 |
| A_92_P008734 | 31.1 | cell.organization | 1750 UP:TBB6_MAIZE (Q41783) Tubulin beta-6 chain (Beta-6 tubulin), complete | -1.02 |
| A_92_P015345 | 31.1 | cell.organisation | 806 similar to UP:Q9FJD5_ARATH (Q9FJD5) Laccase (Diphenol oxidase) (At5g60020), partial (26%) | -0.89 |
| A_92_P003305 | 31.1 | cell.organisation | 846 similar to UP:ADF1_PETHY (Q9FVI2) Actin-depolymerizing factor 1 (ADF 1), complete | -0.81 |
|  |  |  |  |  |
|  |  |  |  |  |
| **Redox** |  |  |  |  |
|  |  |  |  |  |
| A_92_P029619 | 21 | redox | 1447 weakly similar to PRF:NP_566623.1:18402079:NP_566623 oxidoreductase, 2OG-Fe(II) oxygenase family protein (Arabidopsis thaliana), partial (71%) | -1.04 |
| A_92_P007906 | 21.01 | redox.thioredoxin | 969 weakly similar to UP:TRXM_BRANA (Q9XGS0) Thioredoxin M-type, chloroplast precursor (TRX-M), partial (62%) | -0.91 |
| A_92_P030005 | 21.2.1 | redox.ascorbate and glutathione.ascorbate | 1778 similar to UP:Q6ZJ08_ORYSA (Q6ZJ08) Monodehydroascorbate reductase, complete | -0.82 |
|  |  |  |  |  |
|  |  |  |  |  |
| **Minor CHO Metabolism** | | |  |  |
|  |  |  |  |  |
| A_92_P021967 | 3.5 | minor CHO metabolism.others | ACG36394 NAD(P)H-dependent oxidoreductase [Zea mays] | -1.81 |
| A_92_P030063 | 3.1.1.01 | minor CHO metabolism.raffinose family.galactinol synthases.known | 874 homologue to UP:Q5DVS7_MAIZE (Q5DVS7) Galactinol synthase 1 , partial (47%) | -1.23 |
| A_92_P007542 | 3.5 | minor CHO metabolism.others | ACG34871 NAD(P)H-dependent oxidoreductase [Zea mays] | -1.07 |
| A_92_P025409 | 3.1.2.02 | minor CHO metabolism.raffinose family.raffinose synthases.putative | 2536 UP:Q575Z6_MAIZE (Q575Z6) Alkaline alpha galactosidase 3 , complete | -0.80 |
|  |  |  |  |  |
|  |  |  |  |  |
| **Fermentation** | |  |  |  |
|  |  |  |  |  |
| A_92_P013544 | 5.10 | fermentation.aldehyde dehydrogenase | 1903 UP:Q8S529_MAIZE (Q8S529) Cytosolic aldehyde dehydrogenase RF2D, complete | -1.58 |
|  |  |  |  |  |
|  |  |  |  |  |
| **Glycolysis** |  |  |  |  |
|  |  |  |  |  |
| A_92_P004702 | 4.05 | glycolysis.pyrophosphate-fructose-6-P phosphotransferase | 2755 homologue to GB:BAB55499.1:14090340:AP002972 putatative pyrophosphate--fructose-6-phosphate1 phosphotransferase (Oryza sativa (japonica cultivar-group)), partial (67%) | -0.77 |
|  |  |  |  |  |
|  |  |  |  |  |
| **C4 Photosynthesis** | |  |  |  |
|  |  |  |  |  |
| A_92_P000780 | 36.1 | C4.Photosynthesis | 1611 similar to UP:O64910_PEA (O64910) Glucose-6-phosphate:phosphate-translocator precursor, partial (75%) | -0.77 |
| A_92_P029778 | 36.1 | C4.Photosynthesis | 1329 similar to UP:TPT2_BRAOB (P52178) Triose phosphate:phosphate translocator, non-green plastid, chloroplast precursor (CTPT), partial (57%) | -0.76 |
|  |  |  |  |  |
|  |  |  |  |  |
| **Misc.** |  |  |  |  |
|  |  |  |  |  |
| A_92_P004241 | 26.03 | misc.gluco-, galacto- and mannosidases | BAC84503 putative beta-1,3-glucanase [Oryza sativa Japonica Group] | -3.00 |
| A_92_P024063 | 26.1 | misc.cytochrome P450 | ABA97037 Cytochrome P450 family protein, expressed [Oryza sativa (japonica cultivar-group)] | -1.78 |
| A_92_P025599 | 26.1 | misc.cytochrome P450 | (' similar to GP\|15408780\|dbj\|BAB64180. cytochrome P450-like protein {Oryza sativa (japonica cultivar-group)}, partial (9%)), BAD17629 putative thromboxane-A synthase [Oryza sativa Japonica Group] | -1.55 |
| A_92_P041998 | 26.27 | misc.calcineurin-like phosphoesterase family protein | 609 similar to UP:Q53KP2_ORYSA (Q53KP2) At1g18480:F15H18_1, partial (32%) | -1.33 |
| A_92_P022169 | 26.12 | misc.peroxidases | 1513 similar to UP:O81524_AVESA (O81524) Peroxidase PXC2 precursor, partial (85%) | -1.31 |
| A_92_P016764 | 26.1 | misc.cytochrome P450 | 589 similar to UP:Q69NQ0_ORYSA (Q69NQ0) Elicitor-inducible cytochrome P450-like, partial (10%) | -1.30 |
| A_92_P040791 | 26.08 | misc.nitrilases, *nitrile lyases, berberine bridge enzymes, reticuline oxidases, troponine reductases | weakly similar to UP\|Q7G6E7 ORYSA (Q7G6E7) Amidase, partial (34%) | -1.25 |
| A_92_P034521 | 26.11.01 | misc.alcohol dehydrogenases | weakly similar to UP\|Q2QVJ8 ORYSA (Q2QVJ8) Allyl alcohol dehydrogenase, partial (44%) | -1.05 |
| A_92_P008963 | 26.01 | misc.misc2 | 1168 weakly similar to UP:Q9ZP87_TOBAC (Q9ZP87) Epoxide hydrolase, partial (69%) | -1.05 |
| A_92_P036511 | 26.11 | misc.alcohol dehydrogenases | 1535 similar to UP:Q8RV10_ARATH (Q8RV10) AT5g24760:T4C12_30, partial (93%) alcohol dehydrogenase | -0.99 |
| A_92_P004287 | 26.03 | misc.gluco-, galacto- and mannosidases | 384 weakly similar to PRF:NP_567055.1:18410809:NP_567055 glycoside hydrolase family 28 protein : polygalacturonase (pectinase) family protein (Arabidopsis thaliana), partial (19%) | -0.90 |
| A_92_P011382 | 26.12 | misc.peroxidases | 1012 UP:Q9ZTS8_MAIZE (Q9ZTS8) Anionic peroxidase H, complete | -0.83 |
| A_92_P012299 | 26.22 | misc.short chain dehydrogenase/reductase (SDR) | 1230 weakly similar to UP:Q6H7C9_ORYSA (Q6H7C9) Short-chain dehydrogenase:reductase protein-like, partial (56%) | -0.83 |
| A_92_P024442 | 26.10 | misc.cytochrome P450 | 537 weakly similar to UP:C71E1_SORBI (O48958) Cytochrome P450 71E1 (4-hydroxyphenylacetaldehyde oxime monooxygenase) , partial (26%) | -0.78 |
| A_92_P016763 | 26.09 | misc.glutathione S transferases | 1434 UP:Q9ZP61_MAIZE (Q9ZP61) GST6 protein , complete | -0.73 |
| A_92_P016652 | 26.10 | misc.cytochrome P450 | 834 similar to UP:Q8S9F0_ORYSA (Q8S9F0) Cytochrome P450, partial (46%) | -0.72 |
| A_92_P011466 | 26.23 | misc.rhodanese | 944 similar to UP:Q94A65_ARATH (Q94A65) AT4g27700:T29A15_190, partial (77%), rhodanese-like domain containing | -0.71 |
|  |  |  |  |  |
|  |  |  |  |  |
| **No Ontology** | |  |  |  |
|  |  |  |  |  |
| A_92_P021630 | 35.1 | not assigned.no ontology | 898 weakly similar to PRF:NP_566732.1:18403820:NP_566732 dienelactone hydrolase family protein (Arabidopsis thaliana), partial (77%) | -2.47 |
| A_92_P032299 | 35.1 | not assigned.no ontology | 1891 homologue to UP:O48888_MAIZE (O48888) ATP sulfurylase , complete | -2.00 |
| A_92_P022341 | 35.2 | not assigned.uknown | EEF48837 ATP binding protein, putative [Ricinus communis] | -1.74 |
| A_92_P020632 | 35.1 | not assigned.no ontology | weakly similar to RF\|NP 180431.1\|15226916\|NM 128424 acyltransferase {Arabidopsis thaliana} (exp=-1; wgp=0; cg=0), partial (25%) | -1.46 |
| A_92_P010989 | 35.2 | not assigned.uknown | 676 similar to UP:AGA1_YEAST (P32323) A-agglutinin attachment subunit precursor, partial (4%) | -1.45 |
| A_92_P015090 | 35.1.5 | not assigned.no ontology.pentatricopeptide (PPR) repeat-containing protein | 786 weakly similar to UP:Q66GP4_ARATH (Q66GP4) At5g13770, partial (20%) | -1.35 |
| A_92_P012688 | 35.2 | not assigned.unknown | 1439 similar to UP:NRG3_HUMAN (P56975) Pro-neuregulin-3, membrane-bound isoform precursor (Pro-NRG3) [Contains: Neuregulin-3 (NRG-3)], partial (3%) | -1.17 |
| A_92_P019011 | 35.2 | not assigned.unknown | ACG35502 lipopolysaccharide-modifying protein [Zea mays] | -1.16 |
| A_92_P028121 | 35.1 | not assigned.no ontology | 849 weakly similar to UP:Q7XJ26_HORVU (Q7XJ26) Iron:ascorbate-dependent oxidoreductase, partial (41%) | -1.15 |
| A_92_P005678 | 35.1 | not assigned.no ontology | 1639 similar to UP:Q9LGF6_ORYSA (Q9LGF6) 3 -N-debenzoyltaxol N-benzoyltransferase-like, partial (88%) | -1.14 |
| A_92_P026509 | 35.1 | not assigned.no ontology | 982 homologue to UP:Q5ZDA1_ORYSA (Q5ZDA1) BCS1 protein-like, partial (44%) | -1.12 |
| A_92_P039916 | 35.2 | not assigned.unknown | 466 weakly similar to UP:Q9LLM1_WHEAT (Q9LLM1) EF-hand Ca2+-binding protein CCD1, partial (70%) | -1.11 |
| A_92_P021726 | 35.2 | not assigned.unknown | weakly similar to ACG45478 ATP-dependent Clp protease adaptor protein ClpS containing protein [Zea mays] | -1.08 |
| A_92_P041833 | 35.2 | not assigned.unknown | 617 similar to UP:Q5QMP3_ORYSA (Q5QMP3) Fiber protein Fb2-like, partial (48%) | -0.97 |
| A_92_P033860 | 35.1 | not assigned.no ontology | 744 similar to UP:Q5ZDA1_ORYSA (Q5ZDA1) BCS1 protein-like, partial (19%) | -0.91 |
| A_92_P007025 | 35.2 | not assigned.unknown | 1995 weakly similar to UP:WSC3_YEAST (Q12215) Cell wall integrity and stress response component 3 precursor, partial (4%) | -0.89 |
| A_92_P032716 | 35.1 | not assigned.no ontology | 636 weakly similar to UP:Q9SD45_ARATH (Q9SD45) Epoxide hydrolase-like protein (AT3g51000:F24M12_40), partial (18%) | -0.86 |
| A_92_P025394 | 35.1.21 | not assigned.no ontology.epsin N-terminal homology (ENTH) domain-containing protein | 1656 weakly similar to PRF:NP_190238.1:15231451:NP_190238 epsin N-terminal homology (ENTH) domain-containing protein : clathrin assembly protein-related (Arabidopsis thaliana), partial (28%) | -0.80 |
| A_92_P021201 | 35.1.2 | not assigned.no ontology.agenet domain-containing protein | 811 similar to UP:Q500V5_ARATH (Q500V5) At1g09320, partial (6%) agenet-domain containing | -0.79 |
| A_92_P012873 | 35.2 | not assigned.unknown | 1076 similar to UP:Q6L8G3_ORYSA (Q6L8G3) Ferric reductase, partial (45%) | -0.76 |
| A_92_P018888 | 35.1 | not assigned.no ontology | 497 weakly similar to UP:Q69UD7_ORYSA (Q69UD7) 0-deacetylbaccatin III-10-O-acetyl transferase-like, partial (27%) | -0.72 |
| A_92_P012968 | 35.1 | not assigned.no ontology | 1870 PIR:T04136:T04136 cell death suppressor protein lls1 - maize (Zea mays), complete | -0.72 |
| A_92_P039583 | 35.1 | not assigned.no ontology | 866 similar to UP:DDPS5_ARATH (Q570Q8) Dehydrodolichyl diphosphate synthase 5 (Dedol-PP synthase 5) , partial (20%) | -0.71 |
| A_92_P023186 | 35.2 | not assigned.unknown | 648 similar to UP:Q6L8G3_ORYSA (Q6L8G3) Ferric reductase, partial (11%) | -0.71 |
|  |  |  |  |  |
|  |  |  |  |  |
| **Unknown** |  |  |  |  |
|  |  |  |  |  |
| A_92_P035716 | N/A | N/A | 1796 UP:Q8IP68_DROME (Q8IP68) CG31813-PA, partial (9%) | -1.76 |
| A_92_P007969 | N/A | N/A | 308 unknown | -1.67 |
| A_92_P019950 | N/A | N/A | NP_001106265 ZCN26 protein [Zea mays] | -1.62 |
| A_92_P033112 | N/A | N/A | ABA94937 Leucine Rich Repeat family protein [Oryza sativa (japonica cultivar-group)] | -1.54 |
| A_92_P023827 | N/A | N/A | 448 unknown | -1.43 |
| A_92_P008078 | N/A | N/A | 602 similar to UP:Q6NSL3_HUMAN (Q6NSL3) SRRM2 protein (Fragment), partial (4%) | -1.42 |
| A_92_P009246 | N/A | N/A | 301 Rxo-3_G10 subtracted cDNA library of maize inbred line B73 infected with Xanthomonas oryzae pv. oryzicola strain BLS222 Zea mays cDNA clone Rxo-3_G10, mRNA sequence. | -1.39 |
| A_92_P020428 | N/A | N/A | 390 unknown | -1.34 |
| A_92_P024275 | N/A | N/A | 522 similar to PRF:NP_188893.1:15228776:NP_188893 expressed protein (Arabidopsis thaliana), partial (65%) | -1.31 |
| A_92_P016951 | N/A | N/A | 1158 weakly similar to UP:Q9FG96_ARATH (Q9FG96) Gb:AAF04872.1 (AT5g50150:MPF21_17), partial (14%) | -1.31 |
| A_92_P000042 | N/A | N/A | 1360 homologue to UP:Q53NY5_ORYSA (Q53NY5) Expressed protein, partial (46%) | -1.29 |
| A_92_P015427 | N/A | N/A | 777 similar to UP:Q54377_STRLN (Q54377) LmrB protein, partial (6%) | -1.27 |
| A_92_P036560 | N/A | N/A | 587 unknown | -1.27 |
| A_92_P041350 | N/A | N/A | 602 similar to UP:Q4SKD2_TETNG (Q4SKD2) Chromosome 13 SCAF14566, whole genome shotgun sequence. (Fragment), partial (4%) | -1.27 |
| A_92_P033976 | N/A | N/A | 1998 weakly similar to UP:Q93YG9_LYCES (Q93YG9) Insulin degrading enzyme, partial (43%) | -1.27 |
| A_92_P011878 | N/A | N/A | 831 similar to UP:Q852F8_ORYSA (Q852F8) Expressed protein, partial (61%) | -1.23 |
| A_92_P009383 | N/A | N/A | 2710 homologue to UP:AMER1_HUMAN (Q9Y4X0) AMME syndrome candidate gene 1 protein, partial (9%) | -1.18 |
| A_92_P040860 | N/A | N/A | 730 unknown | -1.16 |
| A_92_P031602 | N/A | N/A | 2725 weakly similar to UP:CY24B_HUMAN (P04839) Cytochrome B-245 heavy chain (P22 phagocyte B-cytochrome) (Neutrophil cytochrome B, 91 kDa polypeptide) (CGD91-PHOX) (GP91-PHOX) (GP91-1) (Heme binding membrane glycoprotein GP91PHOX) (Cytochrome B(558) beta | -1.11 |
| A_92_P013206 | N/A | N/A | 577 similar to PRF:NP_197196.1:15237879:NP_197196 expressed protein (Arabidopsis thaliana), partial (76%) | -1.07 |
| A_92_P003021 | N/A | N/A | 915 weakly similar to UP:Q6RJY7_CAPAN (Q6RJY7) Elicitor-inducible protein EIG-J7, partial (69%) | -1.06 |
| A_92_P022186 | N/A | N/A | 1395 similar to PRF:NP_194881.2:30689062:NP_194881 expressed protein (Arabidopsis thaliana), partial (78%) | -1.05 |
| A_92_P003470 | N/A | N/A | 569 GB:AY107589.1:AY107589.1 | -1.05 |
| A_92_P023459 | N/A | N/A | 533 weakly similar to UP:Q5QIS9_HORLE (Q5QIS9) Bx2-like protein, partial (14%) | -1.03 |
| A_92_P037627 | N/A | N/A | 658 similar to UP:Q7XUF9_ORYSA (Q7XUF9) OJ991113_30.7 protein, partial (14%) | -1.03 |
| A_92_P035257 | N/A | N/A | 993 weakly similar to PRF:NP_177728.1:15222928:NP_177728 expressed protein (Arabidopsis thaliana), partial (18%) | -1.01 |
| A_92_P031095 | N/A | N/A | 1002 unknown | -1.00 |
| A_92_P002007 | N/A | N/A | 720 weakly similar to PRF:NP_849433.1:30686484:NP_849433 expressed protein (Arabidopsis thaliana), partial (13%) | -0.99 |
| A_92_P028452 | N/A | N/A | 1608 Zea mays clone EL01N0364D05.c mRNA sequence | -0.99 |
| A_92_P000668 | N/A | N/A | 675 unknown | -0.99 |
| A_92_P027849 | N/A | N/A | 967 similar to UP:Q7XUF9_ORYSA (Q7XUF9) OJ991113_30.7 protein, partial (20%) | -0.99 |
| A_92_P038061 | N/A | N/A | 1313 weakly similar to PRF:NP_193076.2:42566752:NP_193076 expressed protein (Arabidopsis thaliana), partial (83%) | -0.98 |
| A_92_P029044 | N/A | N/A | 1447 weakly similar to PRF:NP_566623.1:18402079:NP_566623 oxidoreductase, 2OG-Fe(II) oxygenase family protein (Arabidopsis thaliana), partial (71%) | -0.98 |
| A_92_P031618 | N/A | N/A | EEF45192 serine-threonine protein kinase, plant-type, putative [Ricinus communis] | -0.97 |
| A_92_P008097 | N/A | N/A | 671 similar to UP:Q5N7Q7_ORYSA (Q5N7Q7) Wall-associated kinase 4-like, partial (18%) | -0.96 |
| A_92_P009470 | N/A | N/A | 1891 Zea mays clone Contig854.F mRNA sequence | -0.96 |
| A_92_P019840 | N/A | N/A | 838 Zea mays clone Contig555.F mRNA sequence | -0.94 |
| A_92_P019482 | N/A | N/A | 1401 unknown | -0.93 |
| A_92_P021845 | N/A | N/A | 1102 weakly similar to PRF:NP_198523.1:15240103:NP_198523 expressed protein (Arabidopsis thaliana), complete | -0.93 |
| A_92_P013902 | N/A | N/A | 1509 homologue to UP:Q5U7K3_9POAL (Q5U7K3) Auxin-induced protein (Fragment), partial (88%) | -0.93 |
| A_92_P028209 | N/A | N/A | 2012 homologue to UP:Q5QMH5_ORYSA (Q5QMH5) Ankyrin-like protein, complete | -0.91 |
| A_92_P032263 | N/A | N/A | 423 unknown | -0.91 |
| A_92_P012278 | N/A | N/A | 1111 weakly similar to PRF:NP_197567.1:15242032:NP_197567 expressed protein (Arabidopsis thaliana), partial (33%) | -0.91 |
| A_92_P011352 | N/A | N/A | 1391 similar to UP:Q9XEY1_TOBAC (Q9XEY1) Nt-iaa2.3 deduced protein, partial (60%) | -0.91 |
| A_92_P039626 | N/A | N/A | 642 similar to UP:Q9AVE0_ARALY (Q9AVE0) SRKb, partial (7%) | -0.90 |
| A_92_P015147 | N/A | N/A | 1386 homologue to UP:Q6YZ10_ORYSA (Q6YZ10) 27k vesicle-associated membrane protein-associated protein-like, partial (87%) | -0.90 |
| A_92_P034241 | N/A | N/A | ACG35134 disulfide oxidoreductase/ monooxygenase/ oxidoreductase [Zea mays] | -0.90 |
| A_92_P007774 | N/A | N/A | 529 unknown | -0.89 |
| A_92_P019263 | N/A | N/A | 1771 weakly similar to UP:Q9SA72_ARATH (Q9SA72) T5I8.2 protein, partial (11%) | -0.89 |
| A_92_P021190 | N/A | N/A | 416 similar to PRF:NP_850272.1:30686956:NP_850272 expressed protein (Arabidopsis thaliana), partial (37%) | -0.88 |
| A_92_P002305 | N/A | N/A | 1354 similar to UP:Q9SCZ4_ARATH (Q9SCZ4) Receptor-protein kinase-like protein, partial (33%) | -0.88 |
| A_92_P009825 | N/A | N/A | 1407 similar to PRF:NP_194681.1:15233608:NP_194681 expressed protein (Arabidopsis thaliana), partial (51%) | -0.88 |
| A_92_P019120 | N/A | N/A | AAS87224 RNA-dependent RNA polymerase readthrough protein [Odontoglossum ringspot virus] | -0.88 |
| A_92_P015675 | N/A | N/A | 1417 UP:Q9M582_MAIZE (Q9M582) Hypersensitive-induced response protein, complete | -0.87 |
| A_92_P032583 | N/A | N/A | 1109 similar to UP:O82115_ORYSA (O82115) Zinc finger protein, partial (49%) | -0.86 |
| A_92_P040003 | N/A | N/A | 1367 similar to PRF:NP_567034.1:18410430:NP_567034 Met-10+ like family protein (Arabidopsis thaliana), partial (52%) | -0.86 |
| A_92_P035814 | N/A | N/A | 1148 unknown | -0.86 |
| A_92_P039237 | N/A | N/A | 2601 Zea mays clone EL01N0327E09.c mRNA sequence | -0.86 |
| A_92_P010023 | N/A | N/A | 1305 similar to PIR:A40505:A40505 early protein EP0 (strain Indiana-Funkhuser or Becker) (Suid herpesvirus 1), partial (5%) | -0.85 |
| A_92_P010734 | N/A | N/A | 776 similar to UP:Q9FM85_ARATH (Q9FM85) Protein kinase-like protein (At5g56460), partial (32%) | -0.85 |
| A_92_P008018 | N/A | N/A | 1513 similar to UP:Q7XQG9_ORYSA (Q7XQG9) OJ000114_01.8 protein, partial (45%) | -0.85 |
| A_92_P026172 | N/A | N/A | ACA64825 SKIP interacting protein 35 [Oryza sativa Indica Group] | -0.84 |
| A_92_P042000 | N/A | N/A | 646 homologue to PIR:G86434:G86434 protein F17F8.23 [imported] (Arabidopsis thaliana), partial (5%) | -0.84 |
| A_92_P037850 | N/A | N/A | ACG28817 protein kinase [Zea mays] | -0.83 |
| A_92_P022247 | N/A | N/A | 757 unknown | -0.83 |
| A_92_P032381 | N/A | N/A | AAA62273 ORF2 | -0.83 |
| A_92_P025905 | N/A | N/A | 1941 UP:Q94CG7_MAIZE (Q94CG7) Seven transmembrane protein Mlo8, complete | -0.83 |
| A_92_P023129 | N/A | N/A | 899 Zea mays clone EL01N0320A07.c mRNA sequence | -0.82 |
| A_92_P011667 | N/A | N/A | 2132 Zea mays clone EL01N0424A03.d mRNA sequence | -0.82 |
| A_92_P018153 | N/A | N/A | 637 similar to UP:O81098_ARATH (O81098) RNA polymerase I, II and III 24.3 kDa subunit (AT3g22320:MCB17_5) , partial (46%) | -0.81 |
| A_92_P014537 | N/A | N/A | 778 unknown | -0.81 |
| A_92_P025305 | N/A | N/A | 3033 homologue to UP:Q6V9T1_ORYSA (Q6V9T1) Glycine dehydrogenase P protein, partial (88%) | -0.81 |
| A_92_P001115 | N/A | N/A | 760 similar to PRF:NP_564277.1:18396294:NP_564277 expressed protein (Arabidopsis thaliana), partial (90%) | -0.81 |
| A_92_P021993 | N/A | N/A | 2340 Zea mays clone Contig306 mRNA sequence | -0.81 |
| A_92_P020996 | N/A | N/A | 526 similar to UP:Q5N8N6_ORYSA (Q5N8N6) SF16 protein-like, partial (11%) chaperonin, putative | -0.81 |
| A_92_P039732 | N/A | N/A | NP_176158 transcription factor-related [Arabidopsis thaliana] | -0.80 |
| A_92_P009724 | N/A | N/A | ACG30427 monooxygenase [Zea mays] | -0.80 |
| A_92_P009015 | N/A | N/A | 1150 Zea mays clone Contig304 mRNA sequence | -0.80 |
| A_92_P011078 | N/A | N/A | 788 homologue to UP:Q5N7Q7_ORYSA (Q5N7Q7) Wall-associated kinase 4-like, partial (20%) | -0.80 |
| A_92_P018195 | N/A | N/A | 523 similar to UP:Q67VH6_ORYSA (Q67VH6) S-receptor kinase PK3-like, partial (7%) | -0.80 |
| A_92_P025352 | N/A | N/A | 1880 similar to UP:Q69SH3_ORYSA (Q69SH3) PTS protein-like, partial (89%) | -0.80 |
| A_92_P033133 | N/A | N/A | 1206 similar to UP:Q4F886_ORYSA (Q4F886) DT-related protein, partial (68%) | -0.79 |
| A_92_P003225 | N/A | N/A | ABZ80144 tumor necrosis factor receptor [Vaccinia virus GLV-1h68] | -0.79 |
| A_92_P007709 | N/A | N/A | 611 similar to PRF:NP_565714.1:18402553:NP_565714 expressed protein (Arabidopsis thaliana), partial (97%) | -0.79 |
| A_92_P012432 | N/A | N/A | 679 unknown | -0.79 |
| A_92_P008542 | N/A | N/A | 217 similar to UP:Q4RSS0_TETNG (Q4RSS0) Chromosome 12 SCAF14999 whole genome shotgun sequence, partial (2%) | -0.79 |
| A_92_P015537 | N/A | N/A | 807 weakly similar to UP:Q07065_HUMAN (Q07065) P63 protein (Cytoskeleton-associated protein 4), partial (4%) | -0.78 |
| A_92_P039828 | N/A | N/A | 449 similar to UP:Q9LJH9_ARATH (Q9LJH9) Gb:AAD25781.1, partial (4%) | -0.78 |
| A_92_P008126 | N/A | N/A | 1491 weakly similar to UP:SPAST_HUMAN (Q9UBP0) Spastin, partial (4%) | -0.78 |
| A_92_P012141 | N/A | N/A | 1594 similar to PRF:NP_568988.2:30697982:NP_568988 2-nitropropane dioxygenase family : NPD family (Arabidopsis thaliana), partial (73%) | -0.78 |
| A_92_P031284 | N/A | N/A | 1787 Zea mays clone Contig967.F mRNA sequence | -0.78 |
| A_92_P030865 | N/A | N/A | 1801 weakly similar to PRF:NP_566658.1:18402564:NP_566658 expressed protein (Arabidopsis thaliana), partial (28%) | -0.78 |
| A_92_P034508 | N/A | N/A | 743 similar to UP:Q5BMC5_CYATE (Q5BMC5) Phosphomannose isomerase, partial (27%) | -0.77 |
| A_92_P023616 | N/A | N/A | 1163 similar to UP:Q9XEY6_TOBAC (Q9XEY6) Nt-iaa4.1 deduced protein, partial (53%) | -0.77 |
| A_92_P013699 | N/A | N/A | 1976 similar to PRF:NP_194432.1:15236947:NP_194432 expressed protein (Arabidopsis thaliana), partial (86%) | -0.77 |
| A_92_P034156 | N/A | N/A | 683 unknown | -0.76 |
| A_92_P039117 | N/A | N/A | 1376 weakly similar to PRF:NP_195917.1:15242576:NP_195917 hydrolase, alpha:beta fold family protein (Arabidopsis thaliana), partial (41%) | -0.76 |
| A_92_P030677 | N/A | N/A | 1569 similar to UP:Q940N6_ARATH (Q940N6) AT5g21070:T10F18_100, partial (91%) | -0.75 |
| A_92_P003366 | N/A | N/A | 726 similar to PRF:NP_568966.1:18424673:NP_568966 expressed protein (Arabidopsis thaliana), partial (19%) | -0.75 |
| A_92_P018433 | N/A | N/A | 841 similar to UP:Q84SH1_ORYSA (Q84SH1) Serine:threonine kinase receptor-like protein, partial (28%) | -0.75 |
| A_92_P002784 | N/A | N/A | 2844 homologue to UP:RL7A_ORYSA (P35685) 60S ribosomal protein L7a, complete | -0.74 |
| A_92_P015312 | N/A | N/A | 784 weakly similar to UP:FIBH_BOMMO (P05790) Fibroin heavy chain precursor (Fib-H) (H-fibroin), partial (4%) | -0.74 |
| A_92_P033895 | N/A | N/A | 823 similar to UP:Q5VQE5_ORYSA (Q5VQE5) Alpha:beta hydrolase-like, partial (31%) | -0.74 |
| A_92_P039755 | N/A | N/A | 207 unknown | -0.74 |
| A_92_P004959 | N/A | N/A | 1535 similar to UP:Q8RV10_ARATH (Q8RV10) AT5g24760:T4C12_30, partial (93%), alcohol dehydrogenase | -0.73 |
| A_92_P005332 | N/A | N/A | 683 unknown | -0.73 |
| A_92_P023201 | N/A | N/A | 817 homologue to PRF:NP_174029.1:15223426:NP_174029 glycine-rich protein (Arabidopsis thaliana), partial (6%) | -0.73 |
| A_92_P007306 | N/A | N/A | 580 unknown | -0.73 |
| A_92_P006792 | N/A | N/A | EEF43744 DNA binding protein, putative [Ricinus communis] | -0.73 |
| A_92_P003133 | N/A | N/A | 470 homologue to PRF:NP_563676.1:18379060:NP_563676 splicing factor Prp18 family protein (Arabidopsis thaliana), partial (20%) | -0.73 |
| A_92_P010273 | N/A | N/A | 1092 similar to UP:Q84QD7_TOBAC (Q84QD7) Avr9:Cf-9 rapidly elicited protein 276, partial (15%) | -0.72 |
| A_92_P001903 | N/A | N/A | 1092 weakly similar to UP:MSI1H_MOUSE (Q61474) RNA-binding protein Musashi homolog 1 (Musashi-1), partial (9%) | -0.72 |
| A_92_P033687 | N/A | N/A | ACG28836 protein kinase domain containing protein [Zea mays] | -0.72 |
| A_92_P022800 | N/A | N/A | 1313 similar to UP:Q9LN49_ARATH (Q9LN49) F18O14.21, partial (57%) | -0.72 |
| A_92_P003707 | N/A | N/A | 2016 homologue to UP:Q75GI8_ORYSA (Q75GI8) Expressed protein, partial (96%) | -0.72 |
| A_92_P022483 | N/A | N/A | 969 similar to GB:BAB92487.1:20804803:AP003303 receptor-like protein kinase-like (Oryza sativa (japonica cultivar-group)), partial (96%) | -0.71 |
| A_92_P039337 | N/A | N/A | 435 unknown | -0.71 |
| A_92_P010955 | N/A | N/A | 1322 homologue to UP:Q75HA8_ORYSA (Q75HA8) Expressed protein (With alternative splicing), partial (28%) | -0.71 |
| A_92_P011500 | N/A | N/A | 430 unknown | -0.71 |
| A_92_P011076 | N/A | N/A | 1925 similar to UP:NRTN_HUMAN (Q99748) Neurturin precursor, partial (7%) | -0.71 |
| A_92_P032944 | N/A | N/A | 1451 weakly similar to UP:Q40713_ORYSA (Q40713) Protein kinase , partial (29%) | -0.71 |
| A_92_P040721 | N/A | N/A | 501 unknown | -0.71 |
| A_92_P016896 | N/A | N/A | 1693 Zea mays clone Contig101 mRNA sequence | -0.71 |
| A_92_P025814 | N/A | N/A | 1730 Zea mays clone Contig405.F mRNA sequence | -0.71 |
| A_92_P007038 | N/A | N/A | YP_001187115 RNAse E [Pseudomonas mendocina ymp] | -0.71 |
| A_92_P026980 | N/A | N/A | 638 similar to GB:AAR27948.1:39545690:AY463691 DUR3 (Oryza sativa (japonica cultivar-group)), partial (14%) | -0.71 |
| A_92_P039847 | N/A | N/A | 1792 Zea mays clone EL01N0532D03.d mRNA sequence | -0.71 |

Supplementary Table 3. Transcripts for Rubisco, Rubisco interacting proteins and PPDK transcripts showing no significant log_2_ fold-change and maintained high average expression levels during the chilling treatment (14°C) in comparison to the control treatment (25°C).

| **Probe ID** | **Log_2_ FC** | **Avg Exp** |
| --- | --- | --- |
|  |  |  |
| **Rubisco Large Subunit (rbcL)** | | |
| TC310187 | 0.130 | 16.031 |
| CD970406 | 0.225 | 7.912 |
| DR961731 | -0.029 | 8.141 |
| CF056299 | 0.188 | 9.980 |
|  |  |  |
| **Rubisco Small Subunit (rbcS)** | | |
| TC286731 | 0.140 | 7.584 |
| TC303914 | 0.467 | 9.972 |
| TC309708 | -0.017 | 7.382 |
| TC284238 | -0.056 | 7.145 |
| TC286728 | 0.230 | 11.772 |
|  |  |  |
| **Rubisco Activase (rca1 and rca2)** | | |
| TC283863 | 0.556 | 10.152 |
| TC300568 | 0.594 | 15.606 |
| TC303023 | 0.635 | 9.909 |
| BG462567 | 0.351 | 8.204 |
|  |  |  |
| **Rubisco Subunit Binding Proteins (cpn60A and cpn60B)** | | |
| TC282522 | 0.062 | 6.879 |
| DR831192 | 0.323 | 8.332 |
| TC296864 | 0.519 | 9.628 |
| TC297378 | -0.013 | 6.926 |
| TC279597 | 0.194 | 12.015 |
| TC279255 | 0.544 | 12.570 |
|  |  |  |
| **Rubisco N-methyltransferase (rmt1)** | | |
| TC314791 | 0.052 | 7.147 |
| TC283589 | 0.213 | 11.768 |
|  |  |  |
| **Rubisco (unit unspecified)** | | |
| TC286732 | 0.030 | 6.993 |
| DR960408 | 0.067 | 7.163 |
|  |  |  |
| **PPDK (pdk1)** | |  |
| CO440422 | -0.071 | 9.958 |
| CD437832 | 0.063 | 6.985 |
| AW360516 | 0.040 | 15.986 |
| CF053666 | 0.114 | 7.049 |
| CD651784 | -0.022 | 6.946 |
| CO462044 | -0.018 | 7.316 |
| AI438490 | -0.015 | 7.290 |
| TC286559 | 0.006 | 11.615 |
| DR824583 | 0.034 | 7.022 |
| TC286518 | 0.000 | 6.899 |
| CD981873 | -0.041 | 7.169 |
| AW288518 | 0.083 | 7.133 |
